# The Genome of *Streptococcus mitis* B6 - What Is a Commensal?

**DOI:** 10.1371/journal.pone.0009426

**Published:** 2010-02-25

**Authors:** Dalia Denapaite, Reinhold Brückner, Michael Nuhn, Peter Reichmann, Bernhard Henrich, Patrick Maurer, Yvonne Schähle, Peter Selbmann, Wolfgang Zimmermann, Rolf Wambutt, Regine Hakenbeck

**Affiliations:** 1 Department of Microbiology, University of Kaiserslautern, Kaiserslautern, Germany; 2 Nano+Bio Center, University of Kaiserslautern, Kaiserslautern, Germany; 3 AGOWA Genomics, Berlin, Germany; University of Hyderabad, India

## Abstract

*Streptococcus mitis* is the closest relative of the major human pathogen *S. pneumoniae*. The 2,15 Mb sequence of the *Streptococcus mitis* B6 chromosome, an unusually high-level beta-lactam resistant and multiple antibiotic resistant strain, has now been determined to encode 2100 genes. The accessory genome is estimated to represent over 40%, including 75 mostly novel transposases and IS, the prophage φB6 and another seven phage related regions. Tetracycline resistance mediated by *Tn*5801, and an unusual and large gene cluster containing three aminoglycoside resistance determinants have not been described in other *Streptococcus* spp. Comparative genomic analyses including hybridization experiments on a *S. mitis* B6 specific microarray reveal that individual *S. mitis* strains are almost as distantly related to the B6 strain as *S. pneumoniae*. Both species share a core of over 900 genes. Most proteins described as pneumococcal virulence factors are present in *S. mitis* B6, but the three choline binding proteins PcpA, PspA and PspC, and three gene clusters containing the hyaluronidase gene, *ply* and *lytA*, and the capsular genes are absent in *S. mitis* B6 and other *S. mitis* as well and confirm their importance for the pathogenetic potential of *S. pneumoniae*. Despite the close relatedness between the two species, the *S. mitis* B6 genome reveals a striking X-alignment when compared with *S. pneumoniae*.

## Introduction

*Streptococcus mitis*, a commensal resident of the upper respiratory tract, is part of the Mitis group of Gram positive bacteria that include one of the major human pathogens *Streptococcus pneumoniae*. *S. mitis* rarely causes disease such as endocarditis [1]–[3]. In contrast, the pathogenicity potential of *S. pneumoniae* is high, leading to pneumonia, meningitis, otitis media, sepsis and bronchitis. The capsule is essential for virulence in *S. pneumoniae*, and 91 capsular types unique to the pneumococcus are known [4], [5]. Major pneumococcal virulence factors include pneumolysin, a hemolytic cytolysin (Ply), the autolysin LytA, and a variety of surface proteins implicated in host cell interaction [6], [7]. Moreover, all *S. pneumoniae* isolates possess choline containing teichoic acids which are the anchor structure of choline binding proteins (CBPs) known to express important functions in murein metabolism and host-pathogen interactions [8]. Most of these genes appear to be absent from *S. mitis* although occasional isolates containing these genes have been described [8]–[10].

*S. mitis* consists of many unrelated lineages according to comparative sequence analysis of selected genes, whereas *S. pneumoniae* strains form a tight cluster of clonal groups [11]–[13]. Each one of these lineages is as distant from a putative ancestor as is *S. pneumoniae*, suggesting that *S. pneumoniae* might be a specialized *S. mitis* clone that has evolved as a residence of the upper respiratory tract. It is a general hypothesis that pathogenic bacteria have evolved from commensal species by the acquisition of virulence genes [14], but this concept has been questioned for *S. pneumoniae* based on the finding that over 700 genes extracted from a comparative analysis of three pneumococcal genomes have no homologous counterpart in other bacteria, whereas this number appears to be marginal in *S. mitis* [12]. Therefore it has been postulated that *S. mitis* has evolved from a pathogenic population as a result of loss of virulence genes. However, only one unfinished genome of an *S. mitis* type strain NCTC12261 has been available for such analyses.

Members of the Mitis group are naturally transformable. This property is reflected by a high degree of variability between *S. pneumoniae* clones on the genomic level. Genomic comparison using microarray technology and calculations based on *in silico* data indicate that different *S. pneumoniae* clones differ from each other by over ten percent of their genes [15]–[17] and that only 46% of all genes might be conserved within the species [18]. Transformation of *S. pneumoniae* with *S. mitis* DNA can easily be achieved in the laboratory, but it is not clear as to what extent this occurs in the natural habitat.

In order to investigate the relationship between the two species, we have analyzed the genome sequence of a representative *S. mitis* in detail. *S. mitis* B6 was chosen for several reasons. It clusters within the group of *S. mitis* according to genomic hybridization data [15], and it has been verified as *S. mitis* by MLST analysis [11]. It is a high level penicillin and multiple antibiotic resistant isolate [19], [20]. The *S. mitis* B6 genome was investigated with emphasis on cell surface components and elements involved in the mobility of genomic material. A detailed comparative analysis was performed with six finished *S. pneumoniae* genomes in order to gain insights into interspecies gene transfer. Moreover, a B6-specific oligonucleotide microarray was designed for comparative hybridization analyses.

## Results and Discussion

### Genome Sequence of *S. mitis* B6: General Features

**Figure 1:**
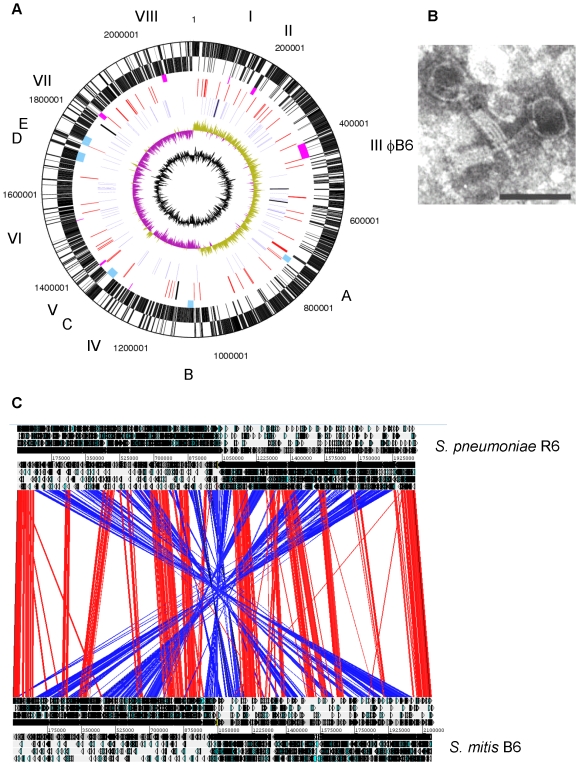
The *S. mitis* B6 genome. A. Circular display of the *S. mitis* B6 chromosome. Black dots mark clusters larger than 15 kb which are absent in most or all *S. pneumoniae* and *S. mitis*; open circles indicate phages related islands. The outer two circles show open reading frames oriented in the forward and reverse direction, respectively. The third circle marks phage related elements including φB6 (pink; roman numbers) and gene clusters >15 kb of the accessory genome which are absent in most or all *S. pneumoniae* and *S. mitis* (blue; A: *ntp* cluster; B: unknown function; C: *Tn*5801; D: *monX* cluster; E: aminoglycoside resistance). The fourth circle shows IS (red) and the two group II introns (black), the fifth circle BOX elements (blue) and RUP (black). The sixth circle shows GC skew, purple indicating negative values; the sixth circle indicates the G+C content. B. Electron micrograph of φB6. Phage particles were purified from mitomycin C-induced *S. mitis* B6 cultures (0.2 µg/ml). The bar reprents 100 nm. C. Genome alignment of *S. mitis* B6 with *S. pneumoniae* R6. In the display using ACT, red areas mark regions of the same orientation in both species, blue indicates regions implicated in the X-alignment. Only regions >1 kb are shown.

General features of the *S. mitis* genome are listed in Table 1. The sequence of the circular genome covers 2,146,611 bp with an average GC content of 39.98% (40.74% for coding sequences) which is similar to the features of finished *S. pneumoniae* genomes (between 2.04 and 2.24 Mb, and around 40% GC) (Fig. 1A). The first base of the *dnaA* gene represents the genome start point, and the putative terminus is located downstream from *xerC*. Both, XerC and XerD bare the unusual conserved sequence motifs described for *S. pneumoniae* [21] involved in an unconventional recombination machinery [22]. There are four rRNA operons. The first 16S rRNA differs from the other three genes in one nucleotide, and there are six respectively seven differences to the 16S rRNA sequences of *S. pneumoniae* R6. Two of the 23S rRNA (smi_0018 and smi_0492) differ from the other two 23S rRNA genes by one nucleotide. Out of the 2018 predicted proteins, biological functions were assigned to 1362 (67%), and 84 had no database match (4%).

**Table 1:** General features of the *S. mitis* B6 genome and comparison with *S. pneumoniae* R6.

	*S. mitis* B6	*S. pneumoniae* R6^a^
total number of bases	2,146,611^b^	2,038,615
GC %	39.98	39
genes (total)	2100	2115
density	0.98 genes per kb	
average length (nt)	908	
coding percentage	87.4	
rRNA	12	12
tRNA	61	58
RNA coding genes	8	3
CDS	2018	2042
average length (aa)	310	
hypothetical proteins	83	
conserved hypothetical proteins	570	

a Only major features are listed. Numbers that are related to the gene number and annotation (hypothetical etc.) are not included due to the early time of annotation in 2001.

b the number refers to the sequenced genome. An additional 7.8 kb are present in *monX* as confirmed by Southern hybridization data as described in the methods section.

Despite the fact that the *S. pneumoniae* R6 genome could be used in an alignment strategy for the assembly of the contigs generated after the shot gun sequencing, the overall arrangement of the *S. mitis* B6 genome reveals a striking arrangement termed X-alignment [23] when compared to *S. pneumoniae* genomes. Fifteen major regions can be recognized where the alignment between *S. pneumoniae* R6 and the *S. mitis* B6 genomes is conserved, interspersed with regions that are symmetrically inverted in respect to the position of the replication origin or terminus (Fig. 1B). At the same time, the preferred location of genes on the leading strand is maintained. Comparison with other *S. mitis* genome sequences suggests that this feature might be common among this species (unpublished results). The reason for this phenomenon is not clear. It has been discussed that the splitting of tandemly repeated sequences by inversion about the origin causes such X-alignment stabilizing the coexistence of duplicated genes [23]. Inversion events have been linked to replication, and the termination process may also contribute to the chromosome architecture [23]–[25]. In this context it should be noted that also in *S. pneumoniae* the genomic synteny is not always maintained: there is a large inversion across the terminus of *S. pneumoniae* CGSP14, where the breakpoints are located within IS elements [26]. However, no repeat sequences are apparent in the *S. mitis* B6 genome, although in several breakpoints defined according to the *S. pneumoniae* R6 genome backbone the insertion element ISSmi1 (see below) is closely associated with these positions on the corresponding sites of the *S. mitis* B6 genome, or IS elements are found in the *S. pneumoniae* genome.

### Mobile and Repeat Elements

**Figure 2:**
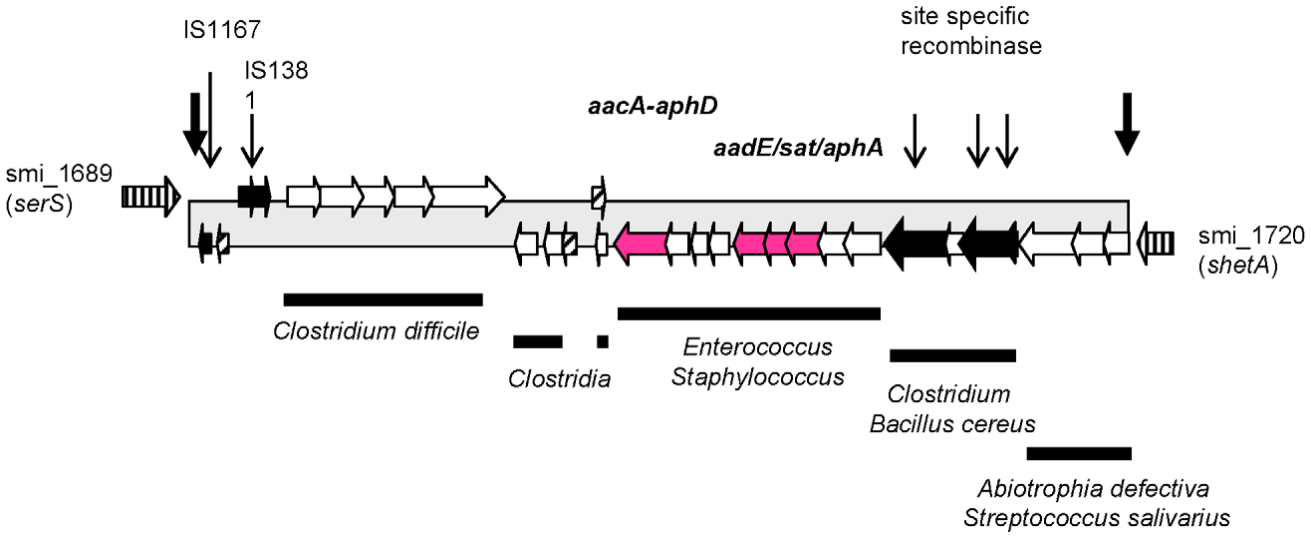
The aminoglycoside resistance gene cluster. The two genes smi_1689 and smi_1720 flanking the cluster are conserved in *S. pneumoniae* and *S. mitis*. Red: genes implicated in antibiotic resistance as indicated above the genes; black: IS and recombinases; hatched: *S. mitis* B6 specific hypothetical genes. Thick lines below mark regions with homology to genes in other species as indicated. The fat arrows left and right mark repeat sequences.

*S. mitis* harbors a large number of elements that are putatively mobile. Among these are 63 recognizable insertion sequences (IS) (Fig. 1 and 2; Table 2): five novel elements, but also some described in other streptococci. The known IS include IS1381, IS861, ISSpn2, ISSsu4 and ISSmu1 from *S. pneumoniae*, *S. suis* and *S. mutans*. The majority of IS in *S. mitis* B6 is made up of the novel element ISSmi1 which is present in 42 complete copies and in one internally deleted variant. ISSmi1 belongs to the IS30 family and is related to another new IS detected in *S. mitis* B6, ISSmi3. The transposases of these elements both consisting of 388 aa share 56% identical residues. The third novel IS found in this genome is ISSmi2, which is peculiar as its transposase gene has no stop codon. Consequently, transposase proteins of varying length are produced upon integration at different sites. It may be worth mentioning that one of the ISSmi2 copies resides within bacteriophage ΦB6 DNA. The other new IS, ISSmi4 and ISSmi5, belong to the IS66 and ISL3 family, respectively. Target duplications are found at the insertion sites of ISSmi1 and ISSmi3, while no target duplication appears to be produced upon integration of the other new IS.

**Table 2:** Mobile genetic elements in *S. mitis* B6.

designation	element	copy number	Reference
ISSmi1	insertion sequence	43	this work
ISSmi2	insertion sequence	4	this work
ISSmi3	insertion sequence	2	this work
ISSmi4	insertion sequence	1	this work
ISSmi5	insertion sequence	1	this work
IS1167	insertion sequence	4	[99]
IS1381	insertion sequence	2	[100]
ISSsu4	insertion sequence	4	[101]
ISSmu1	insertion sequence	1	[102]
ISSpn2	insertion sequence	1	http://www-is.biotoul.fr/is
Tn5801	conjugative transposon	1	[27]
S.m.I.1	group II intron	2	this work
BOX	intergenic repeat unit	103	[30]
RUP	intergenic repeat unit	3	[31], [103]

There may be three additional novel IS in the genome. However we were not able to clearly identify the boundaries of these elements. The remarkable number of IS make up close to 90 kb in the *S. mitis* B6 genome. Curiously, relics of ISSmi1 consisting mainly of the terminal inverted repeats are present in all *S. pneumoniae* genomes sequenced so far. These elements could perhaps be mobilized with a functional ISSmi1 transposase.

A large mobile element originally described in *Staphylococcus aureus* Mu50 as conjugative transposon *Tn*5801 [27] is present in *S. mitis* showing an identity of 99%. *Tn*5801 carries a *tetM* resistance gene (see below) and belongs to an increasing group of elements classified as integrative and conjugative elements (ICE) [28]. *S. mitis* B6 is the first streptococcal strain to harbor *Tn*5801. *Tn*5801 apparently integrated into a 20 bp sequence downstream of *guaA* encoding a GMP-synthase. This short sequence is not present in pneumococcal genomes sequenced so far perhaps explaining the absence of this element in these organisms.

In addition to mobile DNA elements, two copies of group II introns, self-splicing RNAs and retroelements, are contained in the *S. mitis* genome. The *S. mitis* B6 group II intron S.m.I1 belongs to the bacterial class C introns with a group II C structure according to the classification by Dai et al. [29]. S.m.I1 is nearly identical to the pneumococcal S.p.I1 except for a 62 bp deletion removing two variable stem-loops that are not considered functionally important. Like most bacterial group II introns, both S.m.I1 copies are inserted between genes.

The intergenic repeat elements BOX [30] and RUP which are frequently found in *S. pneumoniae* genomes [31] are also present in B6 (Fig. 1 and 2). Whereas BOX elements are abundant, there are only three RUP elements. None of the RUP elements is present at the equivalent position in the R6 genome and two are located at the end of a B6-specific region. This could mean that RUP elements are active elements likely to be associated with gene acquisition in agreement with the proposal that RUP could still be mobile [31]. BOX elements are often present at the same position in both genomes, but examples of extra BOX elements in B6 or *S. pneumoniae* R6, and inversion or expansion/reduction of BOX elements located at the corresponding positions can also be found. Altogether, the mobile elements described above represent over 6% of the *S. mitis* B6 genome.

### The Prophage φB6 and Phage Related Islands

*S. mitis* B6 contains one complete prophage of 44 kb which yields morphologically intact particles of the *Myoviridae* type after induction with mitomycin C (details will be described elsewhere). This phage was named φB6 by Romero *et al.* who received the *S. mitis* B6 strain for analysis of the phage-encoded amidase [32]. Remarkably, so far only one other Myovirus EJ-1 has been described in streptococci isolated from an atypical *S. pneumoniae* [33] whose sequence has been reported recently [32]. The φB6 *lytA* allele which differs from the *S. pneumoniae lytA* sequence in 20% (17% aa) is followed by one copy of ISSmi2 which is part of the prophage. One gene specifies a tyrosine-specific tRNA which may be beneficial to the translational efficiency of the bacterial host. φB6 particles contain circularly permuted, terminally redundant genomes which are likely generated from concatemers by the *pac* mechanism of DNA packaging [34]. Similar to other *pac* phages, the *pac* site is located within the gene for a small subunit of the terminase protein.

Integration of the circularized phage DNA into the host genome occurs within a 9-bp core sequence, common to the attachment sites *attP* and *attB* [35]. The core of *attP* is located 36 bp downstream of the φB6 integrase gene. Prophage integration at *attB* leads to disruption of *ssbB* near its 5′-terminal end. SsbB, a single-stranded DNA binding protein, binds to the incoming donor DNA strand during transformation [36]. Since deletion mutants of SsbB in *S. pneumoniae* have a reduced transformation efficiency, the integration of φB6 into this gene might contribute to the low transformability observed with *S. mitis* B6.

There are another seven phage related gene clusters, six of which are associated with genes encoding complete and degenerate integrases/recombinases (Table 3). Although temperate phages are common in *S. pneumoniae*, the presence of so many relics of phage origin is unusual. φB6 and the other phage remnants of *S. mitis* B6 are unrelated to pneumophages such as the temperate phage ΦMM1 present in the Spain^23F^-1 clone {Croucher, 2009 2435/id}, or other phage remnants of *S. pneumoniae* TIGR4 or Hu19A_6. However, remnants of the phage integrase associated with cluster 7 are found in TIGR4 elsewhere in the genome. Acquisition of MM1 was associated with increased adhesion to eucaryotic cells [38], and it is possible that also oral streptococci benefit from the presence of some phage products. No paralogues of apparent virulence genes are associated with the phage clusters unlike the situation in *Staphylococcus aureus* where prophages might contribute substantially to the virulence potential [39]. The phage-related sequences constitute over 4% of the *S. mitis* B6 genome, confirming that temperate bacteriophages contribute significantly to genome variability in human streptococci as previously postulated for dairy lactic acid bacteria [40].

**Table 3:** Phage related gene clusters.

No	genes	smi	size (kb)	GC %
1^a^	5	0096–0100	3.2	31
2^a^	15	0177–0191	10	32.5
3 φB6^a^	74	0407–0479	44.3	39.8
4^a^	4	1260–1263	1.6	29
5^a^	7	1366–1372	5.3	34.9
6	2	1505–1506	1.1	28
7^a^	16	1781–1795	13.8	31.3
8^a^	18	2000–2017	12.5	38.1

Phage clusters were identified by the presence of two or more coding regions specifying products with significant homology to proteins of known bacteriophages. Size was calculated according to the positions of repetitive sequences (possible integration sites), distinct shifts in the G+C content, and shifts between phage-related and host-related gene functions.

a includes phage related integrase or fragments thereof.

### Antibiotic Resistance

As mentioned above, the tetracycline resistance determinant *tetM* is located on the 25 kb *Tn*5801. The TetM gene is widespread in *S. pneumoniae* but is not associated with this element. In the multiple antibiotic resistant clone Spain^23F^-1, *tetM* is located on *Tn*916 part of which is similar to *Tn*5801, and only *tetM* and short flanking regions are identical to *S. mitis* B6 sequences.

*S. mitis* B6 harbours a remarkable number of genes associated with aminoglycoside resistance, all of which are common among Gram positive cocci. There is a ∼25 kb cluster which includes three such resistance determinants (Fig. 2). It contains genes of the bifunctional enzyme AacA-AphD (aminoglycoside acetyltransferase and phosphotransferase) present in Tn4001-like elements in the genomes or plasmids of Gram-positive cocci [41]. In the near vicinity the three genes *aphA*, *sat* and *aadE* (aminoglycoside 3′-phosphotransferase, streptothricin acetyltransferase, and aminoglycoside 6-adenylyltransferase) are clustered which are also frequently found in *Staphylococcus* and *Enterococcus* as part of *Tn*5405 and pJH1, respectively. In addition, mainly *Clostridium* and *Streptococcus* spp. homologues are located in this region (Fig. 2).

The origin of this cluster is obscure. Among 28 finished and unfinished genomes of *S. pneumoniae* listed in the NCBI data base, only strain CGSP14 contains *aphA* and *sat* together with a truncated version of *aadE*. None of the current finished microbial genomes contains these genes in the combination found in *S. mitis* B6. The three genes *aphA*, *sat* and *aadE* have been identified in viridans streptococci [42]. There are reports where *aacA-aphD* as well as *aphA* and *aadE* were found by PCR analysis within the same *S. aureus* strain [43], [44], but the genomic context is not clear.

*S. mitis* B6 is also rifampicin resistant due to a mutation in RpoB H486N which has frequently been identified in *S. pneumoniae* [45]. It should be noted that in most *S. pneumoniae*, *rpoB* has been annotated differently starting 13 codons upstream and therefore this mutation has been defined as H499N, but in the other Gram positive cocci the size of RpoB corresponds to that in *S. mitis* B6.

All five high molecular weight PBPs of *S. mitis* B6 have been implicated in beta-lactam resistance, but for *pbp1b* and *pbp2a* only partial sequences were described [20]. Surprisingly, *pbp1b* contains an authentic stop codon at position 567 within the penicillin-binding/transpeptidase domain. This explains why PBP1b could not be visualized as beta-lactam complex on SDS-gels [20]. PBP1b is dispensable under laboratory conditions [46], [47], and apparently at least its transpeptidase activity *in vivo* as well. PBP2a is also modified due to the integration of an ISSmi1 element at the very end of *pbp2a* resulting in a two amino acid extension of the C-terminus. However, no impact on protein function is to be expected by this modification.

When compared to PBP genes of penicillin sensitive *S. pneumoniae*, *S. mitis* B6 *pbp1a* and *pbp2b* show a mosaic structure diverging between 3–29%, and *pbp2x* and *pbp1b* diverge by 19–25% throughout. In contrast, flanking regions and *pbp3* differ from the *S. pneumoniae* genes by only 2–10%. The only exception is *pbp2x* where *ftsL* upstream of *pbp2x* also diverges from the *S. pneumoniae* sequence by 23%. This could mean that also in *S. mitis* B6 some PBP genes are the product of gene transfer which occurred during the acquisition of penicillin resistance. However, PBP2x genes are highly variable among penicillin sensitive *S. mitis* ([48] and unpublished results), and thus the evolutionary history of PBP alterations in *S. mitis* B6 is not clear.

### Cell Wall Associated Proteins

Prominent members of cell surface proteins that have been implicated in virulence and host cell interactions of *S. pneumoniae* belong to the families of choline-binding proteins (CBPs) [8] and cell wall bound proteins containing the characteristic LPXTG motif [49]. Members of these two protein families are abundant in *S. mitis* B6, and were therefore analyzed in detail.

*S. mitis* B6 harbors 22 choline binding proteins (CBPs) which exceeds the numbers found in *S. pneumoniae* genomes by far; e.g. there are 12 CBPs in *S. pneumoniae* R6, and 14 in *S. pneumoniae* TIGR4 (Table 4). The presence of CBPs is not surprising, since *S. mitis* B6 contains the *licD1* and *licD2* operons known to be responsible for the choline decoration of the pneumococcal teichoic acid, and a *licD3* homologue as well. This strongly indicates that also in *S. mitis* B6 choline-containing teichoic acids are present. In agreement with the formation of long chains in medium supplemented with 2% choline, *S. mitis* B6 contains homologues of all six CBPs implicated in murein hydrolysis and cell separation in *S. pneumoniae*: LytB, LytC, Pce, CbpD, and the φB6-associated LytA; also a CbpF homologue which has recently been shown to inhibit LytC in *vitro and in vivo* [50] is present. Only Cbp12/13 contain a putative endo-beta-N-acetylglucosaminidase domain, whereas the function of the other *S. mitis* B6 CBPs cannot be deduced.

**Table 4:** CBPs in *S. mitis* and *S. pneumoniae*.

*S. mitis* B6			*S. mitis* a	*S. m.* NCTC12261	*S. p* TIGR4.	*S. p.* R6
smi	gene	aa		(aa)	(aa)	(aa)
0037	*cbp1*	432	0	−	−	−
0038	*cbp2*	500	0	−	−	−
0055	*cbp3*	393	4	+	−	−
0057	*cbp4*	389	2	+	−	−
0086	*cbpI*	395	5	+	(+)	−
0402	*cbp5*	304	10	−	(+)	(+)
0478	*lytA* d	318	4	−	+	+
0725	*cbp6* b,c	352	1	−	−	−
0726	*cbp7* b	366	1	−	−	−
0933	*pce2*	330	1	−	−	−
0934	*pce1*	627	6	+	+	+
0966	*lytB*	570	6	+ (568)	+ (658)	+ (721)
1280	*cbp8*	304	3	−	−	−
1348	*cbp9*	339	5	−	−	−
1467	*cbp10* b	324	3	−	−	−
1479	*cbp11* b	190	9	+ (190)	+ (332)	+ (329)
1563	*lytC*	536	5	+	+	+ (501)
1724	*cbp12* b,d	356	5	−	−	−
1725	*cbp13* b,d	404	5	−	−	−
1748	*cbpF* e	313	6	+	+ (340)	+ (338)
1875	*cbp14* b	498	0	−	−	−
2051	*cbpD*	372	10	+ (369)	+ (448)	+ (448)

The nomenclature follows that of *S. pneumoniae* TIGR4. The number of amino acids is indicated in brackets if significantly distinct from the B6 sequence. +: present; −: absent; (+) three frames (TIGR4 *cbpI*) or remnants (*cbp5*).

a genomic hybridization with ten *S. mitis* strains using a *S. mitis* B6 specific microarray; the number refers to *S. mitis* strains giving positive signals.

b 40mer repeat.

c phage associated.

d present in *S. pneumoniae* CDC3059-06.

e position of SP0377 (*cbpC*) but more similar to SP0391 (*cbpF*).

**Figure 3:**
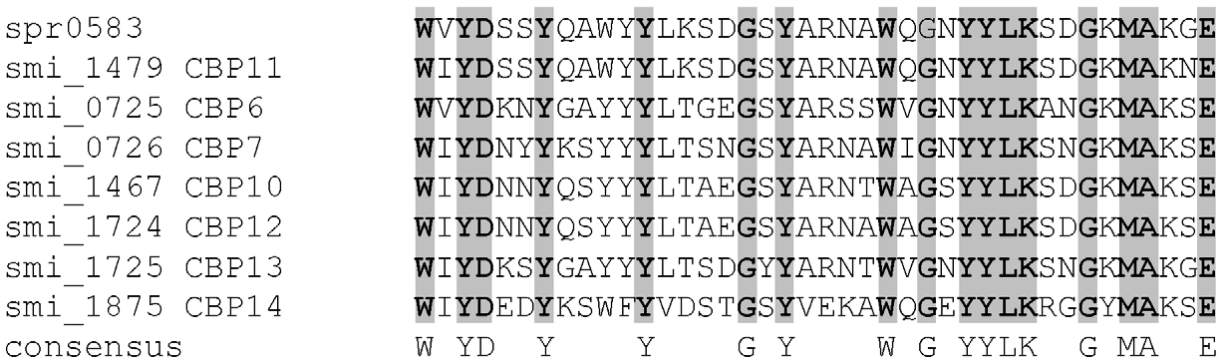
Alignment of the choline-binding modules with 40mer repeat sequences. The *S. pneumoniae* R6 Spr583 (CBP11 homologue) is included.

CBPs are a family of modular proteins with a mostly C-terminal choline binding domain (CBD) responsible for the interaction with the choline-containing teichoic acids. The CBD consists of repetitive choline binding motif (COG5263; glucan-binding domain; YG repeat) with characteristic conserved residues (Pfam accession PF01473). At least two groups of CBPs can be recognized in *S. mitis* B6 on the basis of the ‘repeat’ sequences: those which contain the ‘classical’ 20mer modules (15 CBPs), and a second group (7 CBPs) where only 40mer repeats can easily be aligned instead (Fig. 3) [8]; the ‘repeats’ of LytA, LytB and Pce are highly modified and thus cannot be aligned easily. Only one CBP (CBP11) of the 40mer repeat family is conserved in almost all *S. pneumoniae* (spr0583/SP0677 in R6 and TIGR4, respectively).

There are three examples where the *S. mitis* B6 CBPs are shorter compared to the *S. pneumoniae* homologues, due to variations in the repeat module (CBP11 and CbpD), or the SH3 domain in LytB probably representing another cell-wall interacting domain [51] (see Table 4). CBP11 contains two 40mer repeats in *S. mitis* B6, whereas in most *S. pneumoniae* there are four 40mers and an additional C-terminal extension, and in the *S*. *pneumoniae* Spain^23F^-1 this C-terminal extension is associated with a protein unrelated to CBPs. Interestingly, genes encoding these CBPs are also present in the *S. mitis* NCTC12261 sequence and they are as small as in *S. mitis* B6, suggesting that diversification of CBPs by duplication of repeat modules and recombination events has occurred in *S. pneumoniae*. There are remnants of CBP5 in several *S. pneumoniae* genomes, and its N-terminal region is highly related to PcpA of *S. pneumoniae* CGSP14.

Tandem arrangement of CBPs is common in *S. mitis* B6: there are four pairs of CBPs in *S. mitis* B6. It is tempting to speculate that tandem rearrangement is the result of gene duplication, and indeed the functional domains of Pce2 and Pce1 are appr. 26% identical to each other and not to other CBPs. However, the situation is more complicated, since the non-repeat modules of the CBPs 1/2, CBP6/7, and CBP12/13 are not related pairwise except for very short sequences. All these data document that duplication and recombination events resulting in functional diversification of CBPs are important mechanisms during the evolution of oral streptococci.

There are 18 cell surface proteins bearing the cell wall attachment motif LPXTG [52], covering almost 7% of the coding sequences. Nine of them are homologues to proteins of *S. pneumoniae* R6 which contains only 12 LPXTG proteins (Table 5). Two of the eight B6-specific LPXTG proteins have predicted functions. At the position equivalent to the *S. pneumoniae* IgA protease, a gene encoding another LPXTG-containing protein (smi_1064) is located in B6. Relics of smi_1064 can be found in *S. pneumoniae* in another genomic region (spr0346–0348). One cell surface protein NanA2 contains a central NanA domain which is also present in most *S. pneumoniae*, but in addition it contains B6-specific N- and C-terminal domains of unknown function. The Ser-rich protein MonX (“monster”) and associated genes encoding compounds involved in export and glycosylation of MonX are representatives of the *S. pneumoniae* accessory genome. The cluster differs from that in *S. pneumoniae* TIGR4 by an additional putative glycosyltransferase, and a putative acetyltransferase which however is found in other *S. pneumoniae* strains. It is common among other *Streptococcus* spp.; the *S. gordonii* protein has been described as a platelet binding protein which may be important for oral colonization [53], [54]. MonX and Smi1002 are among the largest proteins of B6, both covering over 4000 amino acid residues.

**Table 5:** Cell wall surface anchor family proteins in *S. mitis* B6 (LPXTG).

		*S. mitis* a	*S. mitis*	*S. pneumoniae*		homology/features^b^
smi (size, aa)	gene		NCTC 12261	R6 gene (aa)	TIGR4 gene (aa)	
0091 (899)		0	+	spr0075 (1171)	SP0082 (857)	hypo, 152mer repeat (4)
0345 (1757)		4	+	spr0440 (1659)	SP0498 (1659)	hypo
0601 (1907)	*nanA*	2	−	spr1536 (1035)	SP1693 (962 stop)	sialidase A domain (neuraminidase A)
0705 (2183)	*prtA*	4	−	spr0561 (2144)	SP0641 (2140)	cell wall-associated serine proteinase PrtA
0810 (979)		0	−	−	−	hypo, coiled-coil domain; KA-rich 77mer repeats (6, deg.)
0979 (1218)		1	−	−	−	hypo, coiled-coil domain; KA-rich 77mer repeats (6)
1002 (4138)		2	(+)	−	−	hypo, Pro-rich; interspersed repetitive domains (95mers)
1064 (1702)		2	−	(−)	(−)	hypo, Pro-rich; interspersed repetitive domains (95mers)
1306 (2474)		1	(+)	−	−	hypo, coiled-coil domain; KA-rich 77mer repeats (8)
1317 (779)		2	(+)	−	−	hypo, Pro-rich; 36mer repeat (4)
1398 (1699)		2	−	−	−	serine protease
1482 (1969)	*zmpB*	4	+	spr0581 (1876)	SP0664 (1881)	zinc metalloprotease
1531 (2997)		6	−	spr1403 (2551)	−	glycine rich protein (87mer repeat, 7)
1534 (2391)	*bgaA*	0	−	spr0565 (2228)	SP0648 (2233)	beta-galactosidase
1537 (2770)		0	−	−	−	N-acetyl-beta-hexosaminidase
1538 (2322)		0	−	spr0328 (1767)	SP0368 (1767 fs)	hypo
1662 (1591)^c^	*monX*	5	−	−	SP1772 (4776)	hypo, Ser-rich repeats
1848 (1298)	*pulA*	9	+	spr0247 (1256)	SP0268 (1280)	alkaline amylopullulanase

aa: amino acids; + homologue present; - absent; (+) variable sequence; (−) fragments.

a genomic hybridization with ten *S. mitis* strains using a *S. mitis* B6 specific microarray; the number refers to *S. mitis* strains giving positive signals.

b hypo: hypothetical; the presence of large coiled-coil domains is indicated; the number in brackets refers to the number of repeats; deg.: degenerate.

c the number corresponds to the sequenced region; the estimated size according to Southern hybridization is approximately 4,200 aa.

Remarkably, several of the LPXTG proteins contain novel repeat sequences of various length. Three B6-specific LPXTG proteins are predicted to be arranged in prominent coiled-coil structures [55] all of which have KA-rich repeats (smi_0810, 0979 and 1306, Fig. S1). Similar proteins are present in *S. thermophilus, S. hemolyticus*, and *Lactococcus reuteri*. Related prolin-rich degenerate repeats are present in two proteins (smi_1002 and 1064), and again homologues are found in other lactic acid bacteria.

The genes encoding B6-specific LPXTG proteins are frequently associated with transposases or IS: smi_0810 is flanked by ISSsu4, smi_1317 is adjacent to *Tn*5801, and smi_1306 and smi_1534 are next to IS1167. Curious is also the accumulation of four LPXTG protein encoding genes in one region: smi_1531, smi_1534, smi_1537 and smi_1538. Three of them are homologues to R6 proteins; however the genes are located at distinct regions of the R6 genome.

### Bacteriocins and Competence

Competence for genetic transformation plays a major role for gene acquisition in *S. pneumoniae* and oral streptococci as well. The regulation of competence and bacteriocin genes involves similar components, and curiously some bacteriocins are part of the competence regulon. In both cases, a two component systems (TCS) which responds to a specific peptide pheromone is responsible for the induction of competence genes or bacteriocins. The peptide pheromones as well as the group II bacteriocins contain a double glycine (GG) N-terminal leader peptide.

**Figure 4:**
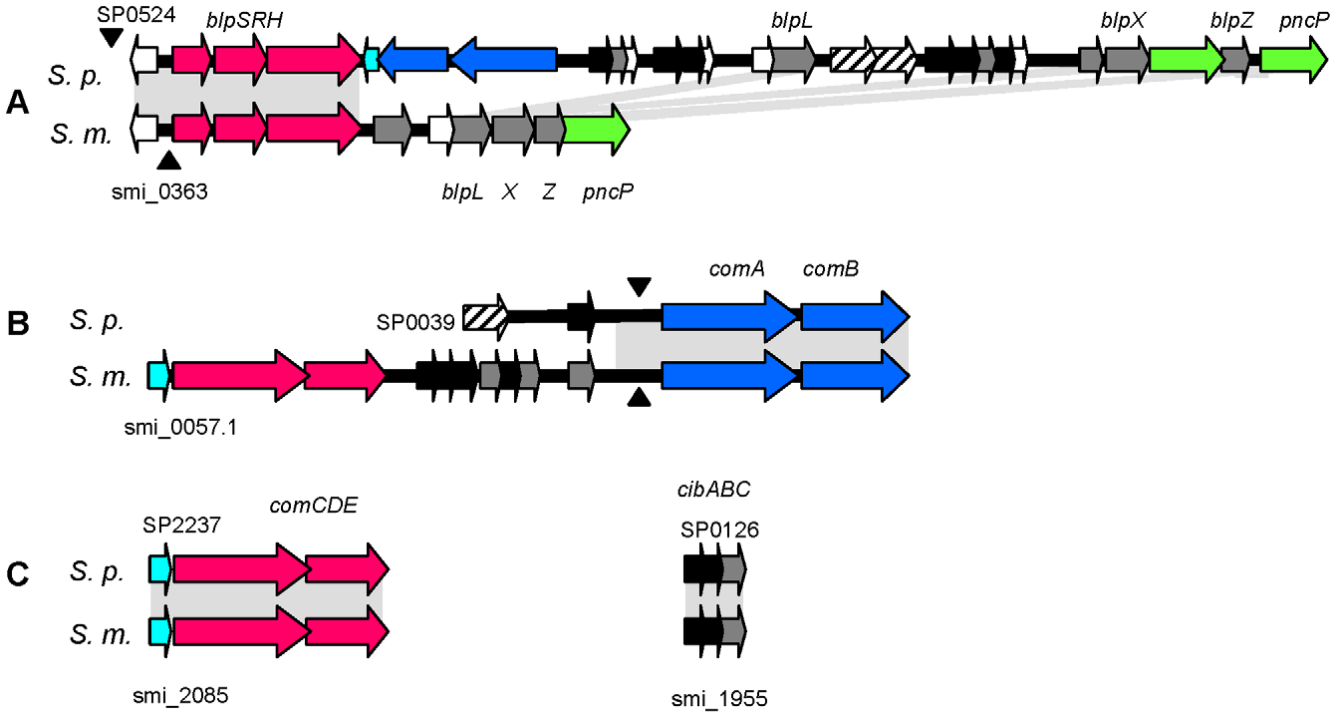
Bacteriocin clusters in *S. mitis* B6. A: the *blp/pnc* cluster; B: cluster II upstream *comAB*; C: components implicated in competence regulation. The gene designation of TIGR4 is given above, and *S. mitis* B6 gene numbers are indicated below. Grey areas indicate regions of >80% identity. Red: Response regulators and histidine kinases; dark blue: ABC peptide transporter; light blue: peptide pheromone; green: CAAX proteases; black: bacteriocins; grey: immunity proteins; striped: IS; white: hypothetical proteins. Black triangles mark the position of BOX elements.

Group II bacteriocins are common among Gram positive bacteria. A highly variable bacteriocin gene cluster has been described in *S. pneumoniae* containing a set of *blp*/*pnc* genes encoding bacteriocins and immunity proteins as well as CAAX proteases [56]–[59]. Adjacent are a regulatory system responsible for induction of the bacteriocins (TCS13; BlpRS/SpiRH), and a peptide pheromone of the GG-family as well as the dedicated ABC transporter involved in export of the pheromone; its role in bacteriocin export is not clear (Fig. 4).

There are two bacteriocin clusters in *S. mitis* which differ substantially from *S. pneumoniae* (Fig. 4). One is related to the *blp*/*pnc* cluster, but although BlpRS are conserved, the pheromone/ABC transporter region is missing, and there are only immunity proteins and a CAAX protease. The second cluster is located upstream of *comAB* encoding the transporter for the competence pheromone peptide CSP, and includes genes encoding bacteriocins/immunity proteins and a TCS with a putative pheromone peptide as well. At this position, a single unrelated bacteriocin gene *blpU* is present in *S. pneumoniae*, and a BOX element upstream of *comAB* is conserved in both species (Fig. 4). In *S. mitis* NCTC12261, variations at this locus compared to B6 concern only number and sequence of the bacteriocins/immunity proteins [51].

Competence is induced via the two component system ComDE in response to high concentrations of CSP, the *comC* product. ComE controls early competence genes including the alternative sigma-factor ComX which in turn regulates late competence genes. Among the ComX regulated proteins are the bacteriocins CibAB, and the immunity protein CibC [60]. CibAB and the ComX responsive cell wall hydrolysase CbpD [61] allow lysis (allolysis) and thus DNA release of non-competent cells of the same strain [61], [62], a process termed fractricide. Since *cibABC* are absent in the *S. mitis* NCTC12261 sequence it has been suggested that fractricide in *S. pneumoniae* has evolved independently from *S. mitis* [62]. In *S. mitis* B6, however, the full repertoire of genes implicated in competence induced lysis including *cibABC*, and other relevant competence genes *comM* and *comW*. All genes are located at the position equivalent to that of the *S. pneumoniae* genome, documenting that this system has evolved prior to the separation of the two species.

It is curious that *S. mitis* B6 contains only one ABC transporter of the peptide-processing family, ComAB, suggesting that it might be involved in the export not only of peptide pheromones but for bacteriocins as well. In fact, *S. mitis* B6 shows bacteriocin activity against several strains of *S. pneumoniae*, *S. mitis* and *S. oralis*, and is competent for genetic transformation albeit only to low transformation efficiency (see below). Alternatively, the CAAX proteases might be involved in bacteriocin/immunity protein processing. A CAAX protease is important to express bacteriocin activity in *S. pneumoniae* [58], and the *Enterococcus faecalis* CAAX protease Eep is required for the processing of a pheromone precursor cAD1 [63]. In this context it should be noted that there are another two bacteriocin genes and one putative immunity protein gene in the *S. mitis* B6 genome, and all three are flanked by a CAAX protease gene. Moreover, B6 contains a third TCS of the Agr family with homology to SarRK involved in lantibiotic production in *S. salivarius*, but no bacteriocin related genes are associated with it. Experimental evidence will be required to understand export and processing of pheromone and bacteriocin precursors.

Unfortunately, we were unable to genetically manipulate *S. mitis* B6 so far in order to investigate gene function via the isolation of non-functional mutants by gene disruption. Due the multiple-antibiotic resistant phenotype, spectinomycin resistance would represent an ideal marker. However, attempts to integrate the spectinomycin resistance gene *aad9* into several loci have failed completely. Only by using DNA of a spontaneous spectinomycin resistant mutant of *S. mitis* B6 containing a mutation in *rpsE* (C70A resulting in Thr21Pro), spectinomycin resistant transformants were obtained with a frequency of ∼10^−4^. The development of suitable genetic tools is subject of current investigations.

### Genomic Comparison with *S. mitis* and *S. pneumoniae*

**Figure 5:**
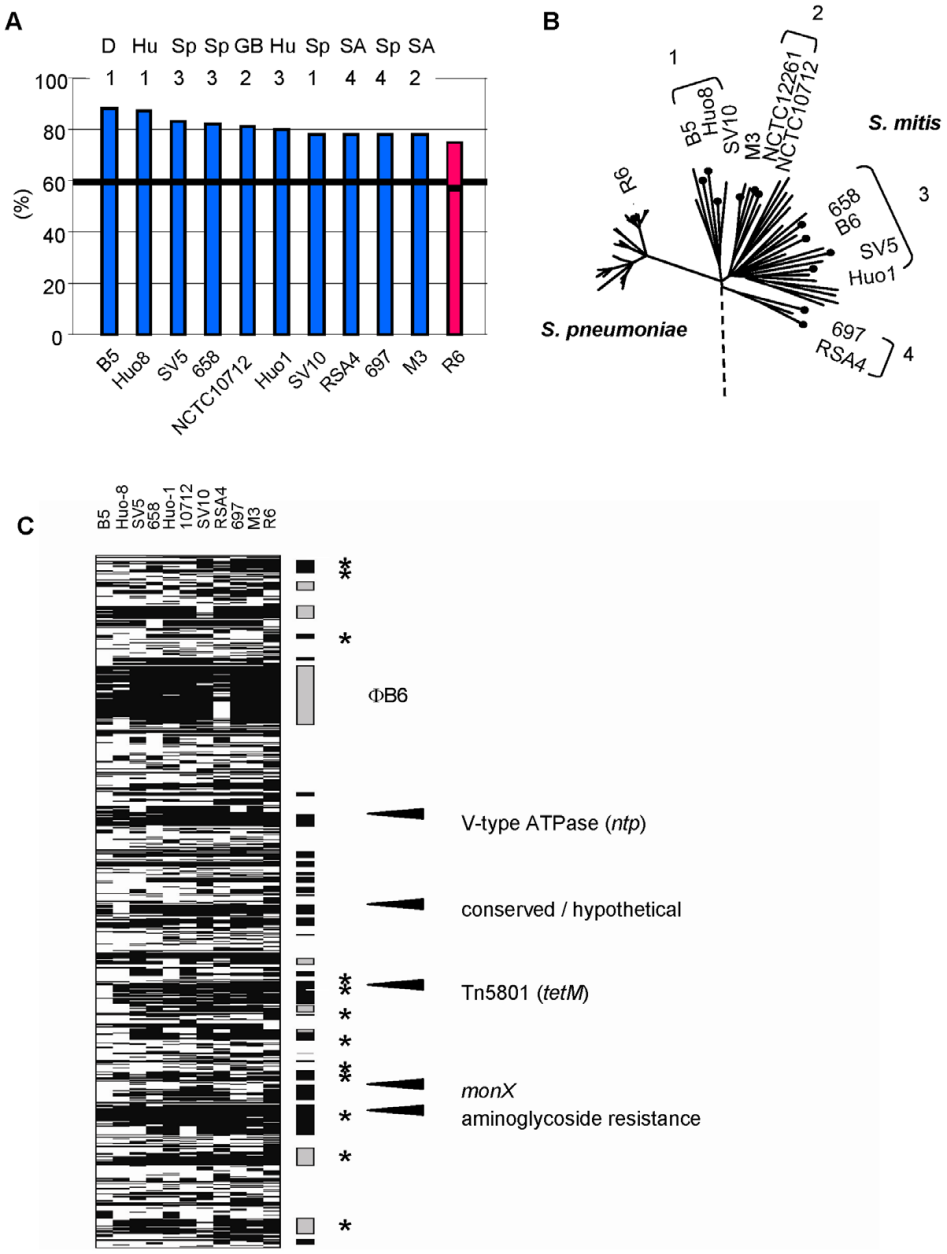
Genomic comparison of *S. mitis* B6. A. Genomic hybridization analysis of *S. mitis* strains using a *S. mitis* B6 specific microarray. Mobile elements and phage related gene clusters, and ambiguous signals were not considered. The percentage of positive hybridization signals is indicated on the left. The vertical fat black line indicates positive genes common to all *S. mitis* strains. The values for *S. pneumoniae* R6 are shown in red. The roman numbers specify the *S. mitis* groups as shown in (B) based on the MLST-derived tree [11]. B. Genetic relationship of *S. mitis*. Geographic origin of the strains: D, Germany; GB: Great Britain; Hu: Hungary; SA: South Africa; Sp: Spain. C. Gene clusters of *S. mitis* B6 as detected by genomic hybridization of *S. mitis* on the B6-specific oligonucleotide microarray. The genes are arranged according to the annotated genome with the replication start on top. Low hybridization signals are indicated by black lines; genes that hybridized with DNA of all strains are not shown. Clusters that are not contained in the B6 strain are marked by boxes on the right, grey boxes indicate phage related gene clusters. * mark the presence of transposases/recombinases. Arrows indicate gene clusters >15 kb.

A comparative genomic hybridization analysis (CGH) of *S. mitis* B6 with other *S. mitis* strains was performed using a B6-specific oligonucleotide microarray. Ten genetically diverse *S. mitis* strains were chosen representing four deep routed branches of the *S. mitis* group [11], [15], and *S. pneumoniae* strain R6 was included as a reference *S. pneumoniae* genome. (Fig. 5A and B). The *S. mitis* strains originated from different geographic areas including East and West European countries and South Africa in order to ensure a broad diversity of the genomes investigated.

Most of the IS elements, recombinases and transposases gave signals in at least one strain. Exceptions were ISSmi1 which hybridized with no *S. mitis*, and large parts of *Tn*5801 were present only in *S. mitis* B5. Also all of the phage related gene clusters including φB6 hybridized with at least one *S. mitis*. Whereas BOX sequences appeared to be present in all *S. mitis*, some strains failed to hybridize with RUP features (Table S2).

Mobile elements and phage related gene clusters were excluded in the following quantitative analysis, leaving 1760 microarray features to be considered. Altogether, 95% of these B6 features hybridized with at least one of the ten *S. mitis*. The presence of several CBPs including CbpD and the *lic1* operon in all *S. mitis* strongly suggested that a choline decorated cell wall and associated proteins are widespread in this species [8], [12]. Individual *S. mitis* hybridized with 88–78% of these features (Fig. 5A). 59% (1039 features) were recognized by all *S. mitis* thus representing the core genome of this strain collection (Fig. 5A; and Table S3), i.e. between 17.5 and 22% of the B6 genes were variably present in individual *S. mitis*. The variation between individual *S. mitis* appears to be not necessarily due to the genetic distance from *S. mitis* B6 or the geographic region of the isolate (Fig. 5B), and indicates that frequent gene transfer results in a highly variable accessory genome among *S. mitis*. Most of the genes that failed to hybridize with the B6 genes are arranged in clusters/regions >4 kb in *S. mitis* (Fig. 5C; and Table S2 and S3).

93 (5%) B6 specific genes remained, including three CBPs (CBP1, 2 and 14), two LPXTG-proteins (smi_1537 and smi_1538) and a bacteriocin gene with associated CAAX protease (Table S2); also the aminoglycoside resistance gene cluster was absent in all other *S. mitis*.

The results concerning *S. pneumoniae* R6 confirm a very close relatedness between the two species: it hybridized with almost all *S. mitis* core genes (973 features), and the same clusters that are variably absent in *S. mitis* can be recognized in *S. pneumoniae* R6 (Fig. 5A and C). The number of features not hybridizing with the mitis core genes is as high as in the two South African *S. mitis* strains (17.5%; Fig. 5A).

**Figure 6:**
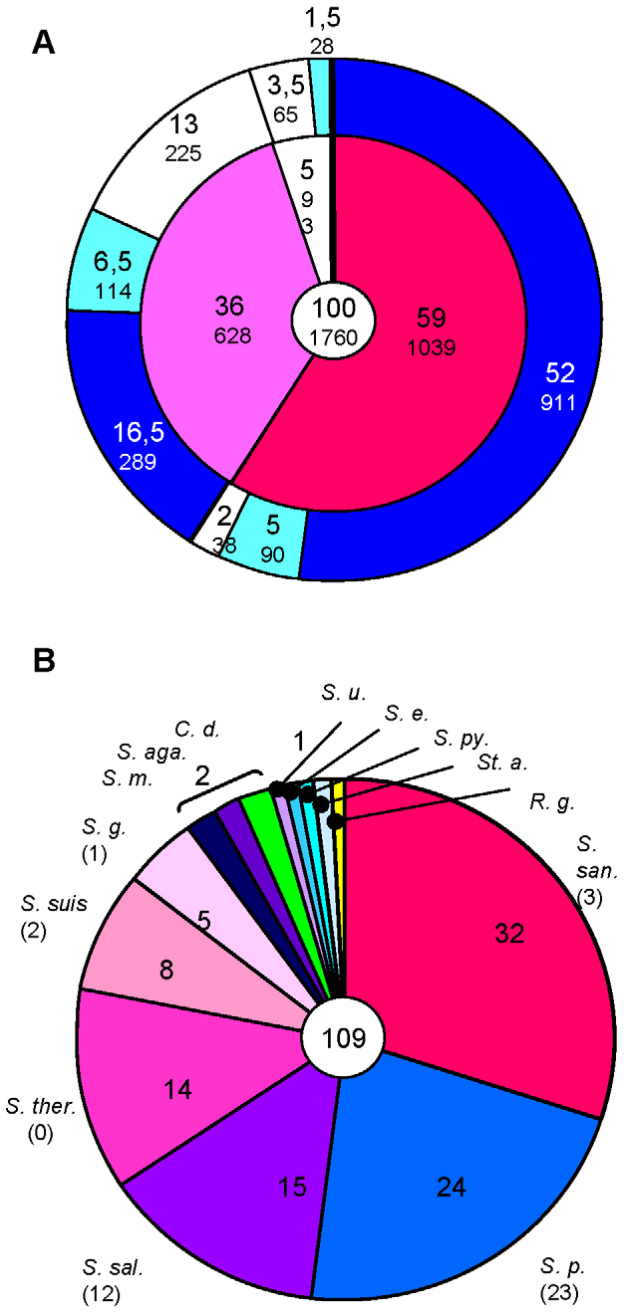
Genomic comparison of *S. mitis* and *S. pneumoniae*. Genes represented on the oligonucleotide microarray (1760 features) excluding mobile elements and phage related gene clusters were used in this calculation. A. Comparison of 1760 *S. mitis* gene products with those annotated in six *S. pneumoniae* genomes. *S. pneumoniae* genomes: see text for details. Inner circle: deep red, percentage of features hybridizing with all ten *S. mitis* strains (*S. mitis* core); light red, with at least one *S. mitis* (*S. mitis* accessory genome); white: no hybridization with any *S. mitis* (B6 specific); outer circle: proteins present in six *S. pneumoniae* genomes according to *in silico* analysis of the annotated gene products, using 70% identity as cut off value and a 60% minimum coverage. Dark blue: genes present in all six *S. pneumoniae* genomes; light blue: genes present in at least one *S. pneumoniae*; white: absent in *S. pneumoniae*. Large numbers indicate the percentage of the 1760 genes represented on the microarray; the number of genes is given in small letters below. B. Homologues of the 109 *S. mitis* B6 genes not present in the six *S. pneumoniae* genomes listed in (A). Only >80% identity values were used, and only species with the best hit are listed. The number in brackets below the species name indicates genes exclusively found in this species. S. aga.: *S. agalactiae*; S. e.: *S. equi*; S. g.: *S. gordonii*; S. m.: *S. mutans*; S. p.: *S. pneumoniae*; S. py.: *S. pyogenes*; S. san.: *S. sanguinis*; S. therm: *S. thermophilus*; S. u.: *Streptococcus uberis*; C.d.: *Clostridium difficile*; R. g.: *Ruminococcus gnavus*; St.a.: *Staphylococcus aureus*.

The data obtained from the CGH were complemented by a comparative *in silico* analysis on the protein level using six finished *S. pneumoniae* genomes of different serotype and MLST sequence type: R6, TIGR4, ATCC700699, G54, CGSP14 and U19A_6 (Fig. 6), in order to see which of the mitis core genes are shared by *S. pneumoniae*, and which genes remain *S. mitis* specific. Altogether, 1432 (81%) out of 1760 *S. mitis* B6 gene products had homologues in *S. pneumoniae*, including 911 (52%) of the *S. mitis* core. On the other hand, over 16% of the accessory *S. mitis* genome was present in all six *S. pneumoniae* strains. These numbers should be taken as a minimum, since the protein homology search used in the *in silico* analysis is less stringent compared to the hybridization results (Table S3).

38 gene products (2%) of the *S. mitis* core had no homologues in *S. pneumoniae* and thus appear to be *S. mitis* specific. This set of genes might be of particular interest in respect of the evolution of *S. pneumoniae*. They include the TCS smi_1072/3 and an adjacent ABC transporter, another two ABC transporters and CBP5 of unknown function, confirming the results obtained with *S. pneumoniae* R6 in the CGH. Close homologues of the regulatory system and the two adjacent genes exist in other oral streptococci such as *S. mutans* and *S. sanguinis*. Curiously, relics of the CBP5 gene are found in the *S. pneumoniae* strains analyzed here. Recently, a full-length homologue of CBP5 (50% identities in the non-repeat module) was reported in a highly virulent serotype 14 *S. pneumoniae* [26] and annotated as PcpA. However, only the repeat domain is very similar to that of PcpA whereas the non-repeat module is distinct, representing another example of the versatility of CBPs. In summary, a minority of the *S. mitis* core genes identified by genomic hybridization distinguishes this species from *S. pneumoniae*.

All genes required for metabolic pathways in *S. pneumoniae* as described in TIGR4 [64] are also present in *S. mitis* B6 with few remarkable exceptions. There are no genes in *S. mitis* B6 for riboflavin biosynthesis (SP0175–0178) and none for thiamine biosynthesis (SP0717-8 and SP0721-5), both of which are arranged in clusters in *S. pneumoniae* except for SP0881 *thiI* which is present in *S. mitis* B6. Whereas all *S. pneumoniae* contain these genes, they are absent in most other streptococci except in *S. agalactiae*, suggesting that they have been imported into an ancient *S. pneumoniae*. On the other hand, *S. mitis* B6 contains an intact gene cluster for L-leucine biosynthesis which is highly fragmented in *S. pneumoniae*, indicating decay in *S. pneumoniae*.

### Comparison with Other Organisms

In order investigate the extent of potential gene transfer events involving other species, a BLAST search of all *S. mitis* B6 gene products for which no homologues in any of the six *S. pneumoniae* genomes were found was performed against all other bacterial genomes including many *S. pneumoniae* genomes not considered in the above analysis. Only identity values >80% over the entire length of the predicted protein were considered. Out of these 338 proteins, 109 fulfilled these criteria (Fig. 6B). 60 were highly related to proteins from at least two streptococcal species, and 24 were found in the other *S. pneumoniae* genomes. Homologues to *S. sanguinis* and *S. salivarius* proteins were predominant and included seven ABC transporters (13 genes). Two proteins related to mercury resistance were *S. gordonii* homologues. The only genes with no homologues in streptococci were four genes located in the aminoglycoside resistance cluster (see Fig. 2), confirming that oral streptococci represent the main source of DNA for expansion of the accessory genome.

### *S. mitis* B6 and Pneumococcal Virulence Factors

Numerous virulence factors have been described in *S. pneumoniae* (for reviews, see [65], [66]). Since especially surface proteins are highly variable and thus might escape the detection as homologous in the above analyses, their presence or absence in *S. mitis* B6 was verified in genomic alignments with *S. pneumoniae* genomes visualized using the ACT programme in addition to in silico genomic comparisons. Surprisingly, *S. mitis* B6 contained the majority of virulence factors involved in colonization and adherence, suggesting that they are important for the interaction with host cells also for commensal bacteria. These include the cell surface wall anchor proteins ZmpB, HtrA and NanA and other surface proteins PavA, Enolase and GAPDH, one pair of the recently described histidine-triad proteins, the two hemolysins HlyX and and HlyIII, the CBPs CbpF, LytB, LytC and Pce, the two peptidyl-prolyl isomerases and lipoproteins PpmA and SlrA, oligopeptide transporters AmiA, AliA and AliB, the manganese transporter PsaA, and as mentioned above the repertoire of genes required or phosphoryl-choline decoration of the cell wall. The IgA protease which is absent in *S. mitis* B6 cannot be regarded as a *S. pneumoniae* specific component, since IgA activity has been found in over 50% of *S. mitis* and the presence of an IgA1 genes has been confirmed in this species [12].

Regulatory proteins are also associated with virulence in *S. pneumoniae*. *S. mitis* B6 contains all but two of the *S. pneumoniae* 13 TCS (TCS04/PhoRP and TCS06 are absent). PhoRP has been implicated in phosphate uptake, but there is another phosphate transport system in *S. pneumoniae* (SP1395–SP1400) which is well conserved in *S. mitis* B6. The strain-to-strain variation observed in the role of TCS04 in virulence indicates that this might contribute to a modulation in pathogenicity potential but is not required for pneumococcal virulence. In addition, 16 out of 25 listed single regulators associated with virulence in *S. pneumoniae* [6] are also present in *S. mitis* B6, and according to theCGH analysis are widespread among *S. mitis*. Nine of these regulators were detected in all ten *S. mitis* strains and the other seven in at least four strains. Most of the regulators absent in B6 are located on *S. pneumoniae* islands coding for PTS systems or ABC transporters and are not part of the pneumococcal core genome such as the *rlrA* islet regulator SP0461 [67]. No homologues of this cluster nor of the second pilus cluster described recently [68] are found in *S. mitis* B6.

*S. mitis* B6 contains also a curious collection of putative virulence genes which are part of the accessory genome of *S. pneumoniae*: clusters encoding a V-type ATPase, and the Ser-rich LPXTG protein MonX. The genomic hybridizations documented that the *monX* cluster is present also in other *S. mitis*. Thus, a repertoire of so-called virulence genes is common to commensal streptococci, probably facilitating and modulating the potential to interact with the host.

*S. mitis* B6 is lacking the iron uptake system Piu/Pia. However, it contains a siderophore-Fe uptake system *tatA/C* which belong to the twin-arginine transport (TAT) system [69] also present in several of the *S. mitis* strains tested on the microarray. A TAT-translocation pathway has been found among streptococci only in the genomes of *S. thermophilus* and *S. sanguinis* [70], [71]. In general, TAT excreted proteins are known to be important virulence determinants in *Pseudomonas and Yersinia* [72], [73]. Interestingly truncated genes of the TAT secretion pathway are found in the genome of *S. pneumoniae*, indicating that also the loss of potential virulence determinants during the divergent evolution of streptococcal species has occurred.

Only a few components are absent in *S. mitis* B6 that are crucial for *S. pneumoniae* pathogenesis: pneumolysin *ply*, the CBPs *pspA*, *pspC*, *pcpA*, and the hyaluronidase *hlyA* in addition to the polysaccharide capsule. No *S. mitis* B6 gene cluster shows signatures related to capsular biosynthesis, also the colony morphology of B6 does not resemble smooth colonies described for encapsulated *S. pneumoniae*, strongly suggesting that it does not carry a complex polysaccharide capsule. The capsule cluster *cps* of *S. pneumoniae* is located between transposase fragments, and is flanked by the conserved genes *dexB* and *aliA*. Most strains of the Mitis group were reported to have large inserts up to 30 kb which could be amplified with primers matching *dexB* and *aliA*, suggesting the presence of a *cps* locus [12]. In agreement with these data, all ten *S. mitis* used in the CGH hybridized with *dexB* specific oligonucleotides (not shown). In contrast, B6 carries only two genes between *dexB* and *aliA*: *glf* encoding a UDP-galactopyranose mutase which can be found in the *cps* cluster of a variety of pneumococci, and a non-functional putative *aliB*-like oligopeptide transporter.

**Figure 7:**
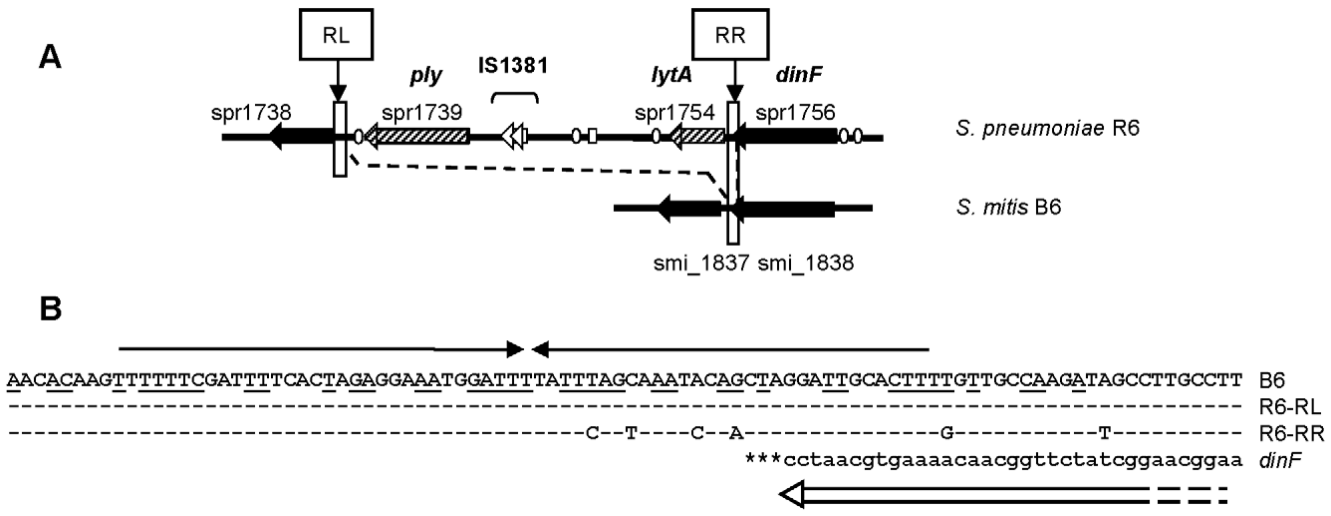
Comparison of the *ply/lytA* island and flanking regions of *S. pneumoniae* R6 and the *dinF* region in *S. mitis* B6. A: Black: conserved genes in *S. mitis* B6 and *S. pneumoniae*; hatched: *S. pneumoniae ply* and *lytA*; ovals: BOX elements; small rectangle: RUP elements; RL and RR designate the left and right direct repeats flanking the *ply/lytA* region (long rectangles). B: Sequences of the left (RL) and right (RR) direct repeat. Arrows above mark the inverted repeat within the direct repeat sequence, and matching nucleotides are underlined; non conserved nucleotides of the *S. pneumoniae* R6 sequences compared to *S. mitis* B6 are shown. The DinF gene is indicated below in small letters and as open arrow.

The gene encoding the pneumolysin Ply, a potent cytolysin, is located close to the autolysin *lytA* on an island which is absent in *S. mitis* B6. Whereas the LytA gene it is under the control of prophage φB6 in *S. mitis* B6, it is part of the competence regulon in *S. pneumoniae*, representing an example that recombination of a partial genetic element can result in its integration into a core regulatory system. The competence regulated induction of *lytA* mediates the release of Ply, a feature related to the virulence of *S. pneumoniae* [61], [74]. Close inspection of the *ply/lytA* region revealed that it is flanked by a 94 bp direct repeat which in itself has an inverted repeat structure (Fig. 7). The repeat is present once in the *S. mitis* B6 genome and covers the 3′-end of the DinF gene plus downstream sequences. This suggests that these repeats represent the integration site of the *ply*/*lytA* region, and the presence of truncated IS elements within that region might be related to this event. In some *S. mitis* a *ply* homologue named mitolysin has been described [9]. Occasional isolates of Mitis cluster have been shown to contain both, *ply* and *lytA* [12], [75], [76], representing valuable material to elucidate the evolution of these genes in *Streptococcus* spp. Also, some of the *S. mitis* strains used in the present study hybridized with *ply* and/or *lytA* specific oligonucleotides (Table S2 and S4). Whereas it has been discussed that *lytA* is a derivative of phages [3], [77], the origin of *ply* is still not known. The presence of the *ply/lytA* island, i.e. the combined activity of the autolysin and the cytolysin, has major impact on the clinical manifestation of pneumococcal disease. Pneumolysin has been shown to affect the integrity of brain endothelial cells and thus is important for damaging the blood brain barrier resulting in pneumococcal meningitis [78]. Pneumococcal meningitis is a life threatening disease, but only very few cases of *S. mitis* meningitis have been reported which occurred primarily after surgical manipulation [79].

Absent in *S. mitis* B6 are also the CBPs *pspA*, *pspC*, *pcpA*, and the hyaluronidase *hlyA*. In order to see whether this is restricted to *S. mitis* B6, the ten *S. mitis* strains described above were used for hybridization analysis with *S. pneumoniae* specific oligonucleotides for these genes. None of the strains hybridized with any of these features (Table S4). The *S. pneumoniae* hyaluronidase gene *hysA* (spr0286/SP0314) is located on a large island adjacent to an IS200-like gene including many components involved in sugar metabolism. The entire island is missing in *S. mitis* B6 in agreement with the finding that *S. mitis* strains do not express hyaluronidase activity [12]. A role in pathogenesis of HysA has been established, but its precise role in pathogenesis is not yet understood [80]. The three CBPs PspA, PcpA, PspC and the PcpC variant Hic interact with host cells and components of the immune system [66]. PspC is regulated by the adjacent TCS06 [81], and again the entire island is missing in *S. mitis* B6. The C-terminal CBDs of both, PcpA and PspA, have significant similarity to the CBD of *S. mitis* B6 Cbp1, suggesting an evolutionary link between these CPBs.

### Concluding Remarks

*S. mitis* B6 represents a striking example for genome modification by the acquisition of genes and gene clusters from other sources. The size of the *S. mitis* B6 genome with over 2.14 MB is far above the average size of 1.8 Mb of *S. mitis* genomes as estimated by PFGE [12] and larger *S. pneumoniae* R6 (2.04 Mb; [82]), suggesting that *S. mitis* B6 has been unusually successful in gene acquisition. Based on hybridization data on the B6-specific oligonucleotide microarray, the accessory genes constitute over 40% of the B6 genome. The large number of phage related gene clusters, mobile elements including *Tn*5801, and the presence of all genes involved in competence and transformation described in *S. pneumoniae* suggests multiple potential routes of gene transfer. Moreover, *S. mitis* B6 appears to be well equipped with bacteriocins facilitating access to foreign DNA by killing and lysing other bacteria.

Among the special features of B6 is the presence of a large number of the same ISSmi1. None of the ten *S. mitis* strains hybridized with ISSmi1, and we found only one copy of ISSmi1 in *S. mitis* NCTC12261, suggesting that expansion of this element has occurred during the evolution of the B6 strain.

We found at least 32 examples of *S. mitis* B6 homologues as remnants in the genomes of *S. pneumoniae* R6 and TIGR4. There is little evidence for gene decay in *S. mitis* B6 (20 truncated genes excluding mobile elements) in comparison to *S. pneumoniae* R6 and TIGR4 which contain 63 respectively 48 truncated genes. This finding is in agreement with the assumption that *S. pneumoniae* originated from an ancient *S. mitis* clone.

*S. pneumoniae* is particularly rich in sugar-related transport systems. *S. pneumoniae* TIGR4 contains 21 PTS systems [64], whereas only 10 PTS systems were found in *S. mitis* B6, five of which are homologues to the *S. pneumoniae* genes. This confirms that *S. pneumoniae* is unusually versatile in sugar uptake, probably related to its distinct habitat, the nasopharynx. The combined properties of a comprehensive sugar metabolism, an efficient immunological protection due to the polysaccharide capsule together with the cytolytic activity due to the ply/lytA island could also be related to its capability to survive well and cause damage in the lung and in the middle ear. The general view of the evolution of pathogens is based on the successive import of virulence genes from sources other than the gene pool provided by related commensals [14]. This could be true in case of *S. pneumoniae* for the hyaluronidase which has not been detected among *S. mitis*, and the non-repeat modules of the CBPs PcpA, PspC and PspA. On the other hand, loss of genes from the *S. mitis* core as defined in the present study might also be important for pathogen evolution, even signifying a ‘route of no return’ to a true commensal life style. These features combined with the expansion of sugar uptake and utilizing systems due to the conquest of its specific ecological niche are the recognizable features distinguishing *S. pneumoniae* from *S. mitis*.

## Materials and Methods

### Bacterial Strains

Bacterial strains are listed in Table S1. *S. mitis* B6 has been described. *S. mitis* and *S. oralis* strains used for comparative genomic hybridization have been characterized by MLST analysis [11].

### Construction of the Shotgun Libraries

For shotgun sequencing three plasmid libraries with small, medium and large inserts, respectively, have been constructed. The small (1.8–2.2 kb) and medium size (4–5 kb) inserts were generated by ultrasonic treatment. After end repair with T4 polymerase (Roche) 10 µg DNA was loaded on an agarose gel (0.9%) and the appropriate size range was cut from the gel. The extracted DNA was cloned into pUC19 cleaved with *Sma*I (Roche).

For the large insert plasmid library the bacterial DNA was partially cleaved with the enzyme *Sau3*AI (Roche). The ends of the fragments were partially filled in and were cloned into the *Sal*I (Roche) cleaved and partially filled low copy vector pMCL210.

### Sequencing and Assembly of the Genome

DNA sequencing reactions were set up using Applied Biosystems BigDye Terminator v3.1 Cycle Sequencing Kit. The shotgun clones were sequenced from both sides using an ABI 3730 XL sequencer up to a 6-fold genome coverage. 60% of the data have been generated from small size insert clones, 30% from medium size and 10% from large insert size plasmid clones. The data assembly was performed using the Staden Package software version 4.6 (Roger Staden, Cambridge, UK). Gap closure was performed by combinatorial PCR followed by sequencing of the generated PCR fragments, using results obtained from alignment of the contigs with the *S. pneumoniae* R6 genome sequence. The sequence includes the MonX gene encoding a highly repetitive 1591 Ser-rich protein. The more precize size of this gene was determined by Southern blot analysis. Chromosomal DNA was digested with restriction endonucleases which cut outside of the repeat regions, and transferred to Hybond N membrane (Amersham) using standard protocols [83]. Two probes unique regions of *monX* were amplified by PCR using the primers MonX-1f and MonX-1r (GAACACTTCTGCGACAGCAACTGAC and CCGCAGAGTTGACCTTAGTGATAGC; 317 bp) and MonX-2f and MonX-2r (CATCAACTGGATCTGTGTTA and ACCGGTACAATGACCGTTAT; 273 bp). Nonradioactive labelling of the amplicon probe, hybridization to the blots, and signal detection were performed according to the instructions provided by the manufacturer (digoxigenin labelling kit, Boehringer, Mannheim). The size of *monX* was estimated at 12.600 bp, i.e. over 7.8 kb longer than the sequenced region, corresponding to approximately 4.200 aa.

### Bioinformatics Analysis

The finished *S. mitis* B6 MG1363 sequence was annotated using Glimmer [84], and tRNA genes were identified with tRNAscan-SE [85]. The initial automatic functional annotation was followed by a manual review of the predicted CDSs, and alterations were made on the basis of the presence of potential ribosomal binding sites, predicted transcriptional terminators [86] and sequence alignments; for RNAs, Rfam was used [87]. All ORFs were searched against the nonredundant nucleotide and peptide sequence databases provided by the National Center for Biotechnology Information using BLAST software [88]. A special search for bacteriocins containing a double glycine leader sequence was performed and small ORFs in their vicinity was examined manually to identify putative bacteriocin and immunity protein genes.

Type I signal peptides were predicted using SignalP 3.0 neural networks and hidden Markov model implementation [89]. For the prediction of transmembrane helices in membrane proteins, TMHMM 2.0 [90] and TMpred [91] were used. Sometimes conflicting results are obtained for the prediction of signal peptides and amino-terminal transmembrane helices in proteins containing a single TM-helix. Therefore these proteins were re-analysed manually. Lipoproteins were identified with the stringent motif used by Sutcliffe and Errington [92]. Proteins using a non-classical secretion pathway were predicted using SecretomeP [93] and PSORTb version 2.0.4 [94] for bacterial protein subcellular localization prediction. A SecP score above 0.5 was considered to be significant [93]. From the output of SecretomeP all known ribosomal proteins, DNA-binding proteins (restriction enzymes, integrases, transcriptional regulators) and phage proteins were removed. Type II lipoprotein signal peptides were identified using PROSITE [95] and the searching motif <[MV]-X(0,13)-[RK]-{DERKQ}(6,20)-[LIVMFESTAG]-[LVIAM]-[IVMSTAG]-[AG]-C as defined by Sutcliffe [96]. PECACE domain harbouring, putative cell wall hydrolases were identified using the motif E-[ST]-X-G-X(1,16)-D-X-M-Q-[SA]-[SA]-E-[SG] [97].

### Comparative Genome Analysis

For comparative analysis with *S. mitis* B6, annotated sequences of *S. pneumoniae* strains R6 [82], TIGR4 [64], ATCC700699 {Croucher, 2009 2435/id}, G54, CGSP14 [26] and U19_6 (http://www.ncbi.nlm.nih.gov/genomes/lproks.cgi) were used. The *S. mitis* NCTC12261 sequence (http://www.jcvi.org/) was annotated automatically; individual ORFs were investigated manually and compared with *S. mitis* B6 using the ACT visualization tool [98] and BLAST analyses.

### Comparative Genome Hybridizations (CGH)

A 70-mer oligonucleotide microarray was designed based on the initial annotation of *S. mitis* B6. The oligonucleotides which were synthesized by OPERON (Huntsville, USA) represent 1978 genes, 461 intergenic regions and 171 controls. Oligonucleotides (30 pmol/µl) were spotted on Nexterion HiSens Slides E (SCHOTT Jenaer Glas GmbH) using the SpotArray TM24 Microarray Spotting System (PerkinElmer) with 32 SMP3-Pins (Telechem). *S. mitis* strains used for CGH have been described [11].

### DNA Labelling and Hybridization

Chromosomal DNA was isolated as previously described [48]. 5 µg of heat denatured genomic DNA was used as a template for direct incorporation of alternate fluorescent analogs Cy5- and Cy3-dCTP (Perkin Elmer) by randomly primed polymerization reaction. Ethanol precipitated labeled DNA was resuspended in hybridization buffer (Nexterion Hyb, Formamid 1:1) and denatured twice at 95°C for 5 min. Hybridization was performed following the manufacturers' recommendations using a hybridization temperature of 40°C for 16 h. Labeled chromosomal DNA of *S. mitis* B6 was used as reference.

### Data Processing

Microarrays were scanned on a laser scanner (ScanArray 4000 Microarray Analysis System, PerkinElmer Life Sciences) with a low resolution of 50 µm using ScanArray Express Software, Version 2.1. Photomultiplier Tube (PMT) was adjusted to balance the two fluorescence channels and biochips were scanned with a 10 µm resolution. Replicate spots that had only background values as estimated from the negative controls included on the microarray were discarded. For each experiment, the fluorescence intensity of the test strain was normalized to that obtained for the B6 reference. A histogram was produced for each data set, resulting in positive (+1) and negative (−1) hybridization signals separated by the diagonal; ambiguous spots (0) which were manually adjusted were not considered in the final analysis. The raw data of *S. mitis* B6 genomic comparison have been deposited in a MIAME compliant database (ArrayExpress accession number E-MEXP-2497).

### Accession Numbers

*Streptococcus mitis* B6 microarray: ArrayDesign B6 ArrayExpress accession A-MEXP-1755. Fully annotated microarray data: ArrayExpress accession number E-MEXP-2497. *Streptococcus mitis* B6 genome sequence and annotation: EMBL FN568063.

## Supporting Information

Figure S1 Repeat sequences of *S. mitis* LPXTG proteins with coiled-coil domains. The sequences strongly predicted to be involved in coiled-coil domain structure of three LPXTG proteins are underlined; in addition Smi_1306 contains C-terminal repeats of low predicted coiled-coil value. The numbers in brackets designate the position of the first amino acid which is shown in the alignment. Amino acids conserved in the majority of the repeats are shown in grey.
(0.07 MB PDF)

Table S1 *S. mitis* strains used for comparative genomic hybridization. Name and references of *S. mitis* strains used in the study.
(0.04 MB DOC)

Table S2 Comparative genomic hybridization on a *S. mitis* B6 specific oligonucleotide microarray using *S. mitis* DNA. There are 2023 features (70mers) included in the analysis, and evaluated as described in the Materials and Methods section. Hybridization signals are indicated by +1 (positive), -1 (negative), or ambigious (0).
(4.52 MB DOC)

Table S3 Genomic hybridization on *S. mitis* B6-specific oligonucleotide microarray data. Only CDS are listed, and mobile elements and phage related gene clusters are not included. Hybridization signals are indicated by +1 (positive, blue), −1 (negative, pink), or ambigious (0). The gene products of six *S. pneumoniae* finished genomes as indicated above were used for an in silico comparative analysis, using 70% identity as cut off value and a 60% minimum coverage. The presence of the gene products is indicated as (x). *S. mitis* A: B5; B: Huo8; C: SV5; D: 658; E: Huo1; F: NCTC10712; G: SV10; H: RSA4; I: 697; K: M3; L: *S. pneumoniae* R6. In silico comparison with *S. pneumoniae* genomes: I: CGSP14; II: R6; III: TIGR4; IV: U19_6; V: G54; VI: ATCC700699. Using the annotated protein sequences, 60% identity and 70% coverage were defined as presence of the respective gene.
(4.36 MB DOC)

Table S4 A. Hybridization of *S. mitis* with oligonucleotides corresponding to *S. pneumoniae* virulence factors. B. Oligonucleotides. Oligonucleotides specific for *S. pneumoniae* R6 (spr) or TIGR4 (SP) were used in comparative hybridization using *S. mitis* DNA. +1: positive signals; negative signals: −1; 0 corresponds to ambiguous signals.
(0.05 MB DOC)
