# Supplementary material for: The Genome of Streptococcus mitis B6 - What Is a Commensal?
Source: PLoS One. 2010 Feb 25;5(2):e9426. doi: 10.1371/journal.pone.0009426 (PMC2828477; doi:10.1371/journal.pone.0009426)
Supplement: Figure S1 — Repeat sequences of S. mitis LPXTG proteins with coiled-coil domains. The sequences strongly predicted to be involved in coiled-coil domain structure of three LPXTG proteins are underlined; in addition Smi_1306 contains C-terminal repeats of low predicted coiled-coil value. The numbers in brackets designate the position of the first amino acid which is shown in the alignment. Amino acids conserved in the majority of the repeats are shown in grey. (0.07 MB PDF) [file pone.0009426.s001.pdf]

smi_0091

(154)

```
VKDSIDVPAGYLEQAKATGPFLAGVNKPLNYELFAGDGMLTRLLLKASDKAPWSDNGVAQNEKIPPV.KNL.PDGKYYYQVSLNGNTTGKQGQDLLDTLRTNGTNTYEATLTVYEAAGDKADLTKVVKERKVNITLNGLVTRSDVNSAVENN
VKDSIDVPAAYLEKAEGKGPFTAGVNQVIPYELFAGDGMLTRLLLKASDKAPWSDNGVAKNPALSPLGENVKTKGQYFYEVDLNGNTVGKQGQDLIDQLRANGTQTYKATVKVYGNKDGKADLTNLVATKNVDININGLVAKETVEKAVKDN
VKDSIDVPAAYLEKAKGEGPFTAGVNQVIPYELFAGDGMLTRLLLKASDKAPWSDNGVAKNPALSPLGENVKTKGQYFYEVDLNGNTVGKQGQDLIDQLRANGTQTYKATVKVYGNKDGKADLTNLVATKNVDININGLVAKETVEKAVKDN
VKDSIDVPAAYLEKAEGKGPFTAGVNQVIPYELFAGDGMLTRLLLKASDKAPWSDNGVAKNPALSPLGENVKTKGQYFYEVDLNGNTVGKQGQALIDQLRANGTQTYKATVKVYGNKDGKADLTNIVATKQVTIKIN
```

smi_0345

(1524)

```
PKLEVEETSIAFERQERPNPNLTVGQRQLVQTGVEGQIRRLIEVDSQGNRTLRATEVLKEASPEIVEVGTGLIPAN.PAP.QNTTLPKPTNQPAYDQQKA
PKLEVQEEKVAFDRQEHENADMLVGEQRIIIQGRDGLLRHVFEVDENGQRRLRSTEVIQEAIPEIVEIGTKVKTEPAVAPTQEKTAQNTAAK
```

smi_0810

(488)

```
                                                                                    TAAKQAITNAANM....KKSAIDSNTGLT
AEEKQTARNAVDAAASQAHQNIDNAIDKTGIDTAKNNGLSAIQNVPTTATYKNTAKQEITAAANA....KKSAIDGQAGLT
AEEKQTAKNAVDAAATQANRNIDNATNQAGVDTAKNNGKQAINAVPANG
        TAKAAVDTEAEKAKAAIDKATDQAGVDSAKAAGKQAIENVPTTATSKDAAKQEITNAAEA....KKSAIDGQAGLT
SEEKAAAKAAVDTEAEKAKAAIDKATDQAGVDSAKAAGKQAIENVPTNGTNATTAKEAAKKAIIDAANKKKAEIDANPALT
KEEKEAAKKAVDAEAEKAKQEIDKATDQAGIDKAKNDGLTAIENVPTNGTNATTAKEAAKKAITDAANKKKAATD
                                     KATDQAGVDSAKVAGKKVIENVPTTATSKDAAKQEITNAAEA....KKSAIDGQAGLT
```

smi_0979

(661)

```
LQKAQAKLEVDKAKKAKLTALEGTPNATKEEKDAAKQAAQDAATKANQAIDAATDNAGVATAQTDGIAAIEAVTPTV
AVKAAAKAEVAKKLAEKLTALEGTPNATKEEKDAAKQAAQDAATKANQAIDAATDNAGVATAQTDGIAAIEAVTPTV
AVKAAAKAEVAKKLAEKLTALEGTPNATKEEKDAAKQAAQDAATKANQAIDAATDNAGVATAQTDGIAAIEAVTPTV
AVKAAAKAEVAKKLAEKLTALEGTPNATKEEKDAAKQAAQDAATKANQAIDAATDNAGVATAQTDGIAAIEAVTPTV
AVKAAAKAEVAKKLAEKLTALEGTPNATKEEKDAAKQAAQDAATKANQAIDAATDNAGVATAQTDGIAAIEAVTPTV
AVKAAAKAEVAKKLAEKLTALEGTPNATKEEKDAAKQAAQDAATKANQAIDAATDNAGVATAQTDGIAAIEAVTPTV
```

smi_1002

(2512)

```
IYRKLGSYVPVVPEGITPPADADLNPKPYPNATPADPTKPGTPTEKPVV.PYIPGTTPVGPNGKPLTPKDPNDPTKGYEVPDLPTDPTENTTITYV
IFRKLGSYVPVIPEGVTPPTGTDLTPKPYENPTNEDPTRPGTPTETPVV.PYIPGTTPVGPSGKPLTPKDPNDPTKGYEVPKVPEDPTQNTTITYV
DSVVNVVYTPLGKYVPKVPEGFE.VPKVEKPQYPNDPTDPTKPGTPTTVIPYVPGTTPKDPNGNPLKPVDPNDPSKGYVPPT.PENPTEDTQITYEKDTQKAKVTYVVEGTGTVLHTDNLEGKSG
EPTDTPYENVPGEPGKVVPPSPTKPQDPQDPNSPKVPVIPHIPGTTPVVPKDPTKPISPDNPLVPLTPVDPNDPTKGYEVPPVPTDPSTDTPITYV
```

ETIKDINETVIYDKLGSWIPNIP.GTPTNPITYPNDPKDPTQPGTDKPKVPYVPGFIPVDPEGQPLKPVDPNDPTKGYEVPEVPGDPTKDTPINYIPKDPTPN

smi_1064

(1160)
VVYTKLGSWVPKVPGVETPTPLPYPNHPTDPTKPGDPTDPNTPNVPVIPYVPGYTPKIGETPLEPVDPNNPENGYKVPPIPNTPGEDTPIEYSKDPQKAVVKVFNTTTGTEVELPEEKVELSGKTGEPIPADSVTAKIAD
VVYKKLGSWIPKVPGVETPTPLPYPNHPTDPTKPGDPMYPNTPNVPVIPYVPGYTPKIGETLLQPKVPNDPTQGYI.PPVPTEPGKDTPIEYVKVPNTPSPEEPVKPTPESNHITIWVDENGKPLKPEKPGTHEPGNIPGY

smi_1306

(920)
KQPAIDAVNTAATEKIAAINSTPNATDEEKAAAIAKVNADKEKALTAI.........NDANVTTKAELDKAKEAGTTAIAGDNPVVAK
KAAAKADVEAARQAKADAINADKNLTQAEKDAALAKVNKAAEDATKAI...........DAATTDAAVDTAKGTGVTEIGKVNPVA
KQNAKDAIGNALTAKNQEIDDRKDLTQAEKDAAKAEAKKLADAELTKVNAQPDNAETAEAAAAAQKLVNDAEDKGVADVTSVYPIA
KEEAKKAVADELAKKEKELDDRKDLTTEEKAAAKKEAKDLAKKATDAINAQPAIADTSDKATAAQQAVDTAKTTGVAEVKAVNPEAVK
KNVAKKAIEDALTAKNNAIDARDDLTPEQKTAAKEAAKAKADAAKDAI...........DKATTDADVDQAKTDGETAVANVTPVA
KKPAKQAIAKALEDKNAEIDKRTDLTEEEKATAKKEAKDKADAQLAEIAKQPDVADTPEAAQTAQTAVDAAKKTGVDEVTAVNPTAVT
KPEAKKAIDAKLAEQLKTIESTPDATDDEKKVAADAAKALAAKAKAEI...........DKAGTDADVKALEDEAKAEIEKSLPLVED
KPNARKAIDEEATAKKAAIDSRTDLTP

(1601)
DADGNITVTTPDGHQETITPEQVVKTSDTANEPKAGNDVVKPANKVVVADPAKLTDAEKEKIKAAVEAVNPNSVVTV
DEKGNAKVSTPDGQTQVIPVDELVRTAEDTKKPNAGNDINTPSDKTVVANPEALTPEEKKAIEDKVKAVNPGATVVV
DDKGNATVTTPSGKTAVIPAADLTKTAEAAAKPNAGDDVVKPASKTVVANPEALTPEEKKAIEDKVKAVNPGATVVV
DDKGNATVTTPSGKTAVIPAADLTKTAEAATKPNAGDDVVKPASKTVVANPEALTPEEKKAIEDKVKAVNPGATVVV
DDKGNATVTTPSGKTAVIPAADLTKTAEAASKPNAGDDVITPASKTVVANPEALTPEEKKAIEDKVKAVNPGATVVV
DDKGNATVTTPSGKTAVIPAADLTKTAEAATKPNAGDDVVKPASKTVVANPEALTPEEKKAIEDKVKAVNPGATVVV
DDKGNATVTTPSGKTAVIPAADLTKTAEAVAKPNAGNDINTPAVKTAVAKKDALTDSEKEAVKTAVEAVNPGAKVVV
DDKGNATVTTKDGKTAVIPASQLVIPTEKLADLSEQNGVNIPATRTLVADKNNLTPEEIAKIKVAVEAVNPGSTVVV
DEKGNATVTTVDGKTATISAEQLVKDEADVATKNNGENLNLDFEKQLVADLDNLTDADKAGAKAKIMAANPDVAEVI

smi_1317

(692)
PKVEA
PKVEA
PKVEA
PKVEA
PKVEV

smi_1398

(1457)
KVEIPTKPVAPVKPVDPTKAVAPVKPVIPAKPVVTI
KPVTPTKPVVTIKPVTPTKPVASIKPVTPAKPVVTV
KPVTPAKPVVTIKPVTPTKPVVTVKPVTPAKPVVVI
KPVTPTKPVVTIKPVIPAKPVVTLNPVIPTKPVSQV

>smi_1531

(1301)
KGDKGDKGEQGLQGRDGQDGAQGLPGRDGRDGAAGRDGRDGRDGKDVLDGKVNPEANQGKDGDKYVNTETGDVFVKNNGNWDKEGNI
KGPKGDKGDQGLQGRDGQDGAQGLPGRDGRDGAAGRDGRDGRDGKDVLNGKVNPEANQGKDGDKYVNTETGDVFVKNNGNWDKEGNI
KGPKGDKGAEGAQGPKGADGAQGLPGRDGRDGAAGRDGRDGRDGKDVLNGKVNPEANQGKDGDKYVNTETGDVFVKNNGNWDKEGNI
KGPKGDKGDQGLQGRDGQDGAQGLPGRDGRDGAAGRDGRDGRDGKDVLNGKVNPEANQGKDGDKYVNTETGDVFVKNNGNWDKEGNI
KGPKGDKGDQGLQGRDGQDGAQGLPGRDGRDGAEGRDGRDGRDGKDVLNGKVNPEANQGKDGDKYVNTETGDVFVKNNGNWDKEGNI
KGPKGDKGDQGLQGRDGQDGAQGLPGRDGRDGAAGRDGRDGRDGKDVLNGKVNPEANQGKDGDKYVNTETGDVFVKNNGNWDKEGNI
KGPKGDKGDQGLQGRDGQDGAQGLPGRDGRDGAEGRDGRDGRDGKDVLNGKVNPEANQGKDGDKYVNTETGDVFVKNNGNWDKEGNI

>smi_1662
(999)
SASTSASKSASTSASQ (>18)

SASTSASESASTSASA (>14)
