# Supplementary material for: The Genome of Streptococcus mitis B6 - What Is a Commensal?
Source: PLoS One. 2010 Feb 25;5(2):e9426. doi: 10.1371/journal.pone.0009426 (PMC2828477; doi:10.1371/journal.pone.0009426)
Supplement: Table S1 — S. mitis strains used for comparative genomic hybridization. Name and references of S. mitis strains used in the study. (0.04 MB DOC) [file pone.0009426.s002.doc]

Table S1. *S. mitis* strains used for comparative genomic hybridization

| Organism and Strain | Country | Year |
| --- | --- | --- |
| *Streptococcus mitis* |  |  |
| M3 | South Africa | 1986-1987 |
| RSA4 | South Africa | 1986-1987 |
| SV5 | Spain | 1992 |
| SV10 | Spain | 1992 |
| 658 | Spain | 1992 |
| 697 | Spain | 1993 |
| Huo1 | Hungary | 1990-1992 |
| Huo8 | Hungary | 1990-1992 |
| B5 | Germany | 1994 |
| B6 | Germany | 1994 |
| NCTC10712 | Great Britain | 1967 |

All strains have been characterized by MLST analysis [11]
