# Supplementary material for: The Genome of Streptococcus mitis B6 - What Is a Commensal?
Source: PLoS One. 2010 Feb 25;5(2):e9426. doi: 10.1371/journal.pone.0009426 (PMC2828477; doi:10.1371/journal.pone.0009426)
Supplement: Table S2 — Comparative genomic hybridization on a S. mitis B6 specific oligonucleotide microarray using S. mitis DNA. There are 2023 features (70mers) included in the analysis, and evaluated as described in the Materials and Methods section. Hybridization signals are indicated by +1 (positive), -1 (negative), or ambigious (0). (4.52 MB DOC) [file pone.0009426.s003.doc]

Table S2. Comparative genomic hybridization on a *S. mitis* B6 specific oligonucleotide microarray using *S. mitis* DNA.

*S. mitis S. pneumoniae*

| **reference B6** | **B5** | **Huo8** | **SV5** | **658** | **Huo1** | **NCTC**  **10712** | **SV10** | **RSA4** | **697** | **M3** | **R6** |
| --- | --- | --- | --- | --- | --- | --- | --- | --- | --- | --- | --- |
| BOXAB | 1 | 1 | 1 | 1 | 1 | 1 | 1 | 1 | 1 | 1 | 1 |
| BOXBC | 1 | 1 | 1 | 1 | 1 | 1 | 1 | 1 | 1 | 1 | 1 |
| ribonuclease P - RNA component | 1 | 1 | 1 | 1 | 1 | 1 | 1 | 1 | 1 | 1 | 1 |
| rup1 | 0 | 1 | -1 | 1 | -1 | -1 | -1 | 1 | -1 | -1 | -1 |
| rup2 | 0 | -1 | -1 | 1 | 1 | 1 | -1 | 0 | -1 | -1 | 0 |
| rup3 | 1 | 0 | -1 | 0 | 0 | 0 | -1 | 0 | -1 | -1 | -1 |
| tmRNA | 1 | 1 | 1 | 1 | 1 | 1 | 1 | 1 | 1 | 1 | 1 |
| scRNA, 4.5S RNA signal recognition particle | 1 | 1 | 1 | 1 | 1 | 1 | 1 | 1 | 1 | 1 | 1 |
| smi_0001 | 1 | 1 | 1 | 1 | 1 | 1 | 1 | 1 | 1 | 1 | 1 |
| smi_0002 | 1 | 1 | 1 | 1 | 1 | 1 | 1 | 1 | 1 | 1 | 1 |
| smi_0003 | 1 | 1 | 1 | 1 | 1 | 1 | 1 | 1 | 1 | 1 | 1 |
| smi_0004 | 1 | 1 | 1 | 1 | 1 | 1 | 1 | 1 | 1 | 1 | 1 |
| smi_0005 | 1 | 1 | 1 | 1 | 1 | 1 | 1 | 1 | 1 | 1 | 1 |
| smi_0006 | 1 | 1 | 1 | 1 | 1 | 1 | 1 | 1 | 1 | 1 | 1 |
| smi_0007 | 1 | 1 | 1 | 1 | 1 | 1 | 1 | 1 | 1 | 1 | 1 |
| smi_0008 | 1 | 1 | 1 | 1 | 1 | 1 | 1 | 1 | 1 | 1 | 1 |
| smi_0009 | 1 | 1 | 1 | 1 | 1 | 1 | 1 | 1 | 1 | 1 | 1 |
| smi_0010 | 1 | 1 | 1 | 1 | 1 | 1 | 1 | 1 | 1 | 1 | 1 |
| smi_0011 | 1 | 1 | 1 | 1 | 1 | 1 | 1 | 1 | 1 | 1 | 1 |
| smi_0012 | 1 | 1 | 1 | 1 | 1 | 1 | 1 | 1 | 1 | 1 | 1 |
| smi_0013 | 1 | 1 | 0 | 1 | 1 | 1 | 1 | 1 | 1 | 1 | 1 |
| smi_0015 | 1 | 1 | 1 | 1 | 1 | 1 | 1 | 1 | 1 | 1 | 1 |
| smi_0020 | 1 | 1 | 1 | 1 | 1 | 1 | 1 | 1 | 1 | 1 | 1 |
| smi_0021 | 1 | 1 | 0 | -1 | 1 | 1 | 1 | 1 | 1 | 1 | 1 |
| smi_0022 | 1 | 1 | 1 | 1 | 1 | 1 | 1 | 1 | 1 | 1 | 1 |
| smi_0024 | 1 | 1 | 1 | 1 | 1 | 1 | 1 | 1 | 1 | 1 | 1 |
| smi_0026 | 1 | 1 | 1 | 1 | 1 | 1 | 1 | 1 | 1 | 1 | 1 |
| smi_0027 | 1 | 1 | 1 | 1 | -1 | -1 | -1 | -1 | -1 | -1 | -1 |
| smi_0028 | 1 | 1 | 1 | 1 | 1 | 1 | 1 | 1 | 1 | 1 | 1 |
| smi_0029 | -1 | -1 | -1 | -1 | -1 | -1 | -1 | -1 | 1 | -1 | -1 |
| smi_0030 | 1 | 1 | 1 | 1 | 1 | -1 | 1 | 1 | 1 | 0 | 0 |
| smi_0031 | 1 | 1 | 1 | 1 | 1 | 1 | 1 | 1 | 1 | 1 | 1 |
| smi_0032 | -1 | -1 | -1 | -1 | -1 | -1 | -1 | -1 | -1 | -1 | -1 |
| smi_0033 | 1 | 1 | 1 | 1 | 1 | 1 | 1 | 1 | 1 | 1 | 1 |
| smi_0034 | 1 | 1 | 1 | 1 | 1 | 1 | -1 | 1 | -1 | -1 | -1 |
| smi_0035 | 1 | 1 | 1 | 0 | 1 | 0 | -1 | 1 | -1 | -1 | -1 |
| smi_0036 | 1 | 1 | 1 | 1 | 1 | 1 | -1 | 1 | -1 | -1 | -1 |
| smi_0037 | -1 | -1 | -1 | -1 | -1 | -1 | -1 | -1 | -1 | -1 | -1 |
| smi_0038 | 0 | -1 | 0 | -1 | -1 | -1 | -1 | -1 | -1 | -1 | -1 |
| smi_0039 | -1 | -1 | -1 | -1 | 1 | -1 | -1 | -1 | -1 | -1 | 1 |
| smi_0040 | -1 | -1 | -1 | -1 | -1 | -1 | -1 | -1 | -1 | -1 | 1 |
| smi_0041 | -1 | -1 | -1 | -1 | -1 | -1 | -1 | -1 | -1 | -1 | 1 |
| smi_0043 | -1 | -1 | -1 | -1 | -1 | -1 | -1 | -1 | -1 | -1 | -1 |
| smi_0044 | -1 | -1 | -1 | -1 | -1 | -1 | -1 | -1 | -1 | -1 | -1 |
| smi_0045 | -1 | -1 | -1 | -1 | -1 | -1 | -1 | -1 | -1 | -1 | -1 |
| smi_0046 | -1 | -1 | -1 | -1 | -1 | -1 | -1 | -1 | -1 | -1 | -1 |
| smi_0047 | 1 | 0 | 1 | 1 | 1 | 1 | -1 | 1 | -1 | -1 | -1 |
| smi_0048 | 1 | 1 | 1 | 1 | 1 | 1 | 1 | 1 | 1 | 1 | 1 |
| smi_0049 | 1 | 1 | 1 | 1 | 1 | 1 | 1 | 1 | 1 | 1 | 1 |
| smi_0050 | 1 | 1 | 1 | 1 | 1 | 1 | 1 | 1 | 1 | 1 | 1 |
| smi_0051 | 1 | 1 | 1 | 1 | 1 | 1 | 1 | 1 | 1 | 1 | 1 |
| smi_0052 | 1 | 1 | 1 | 1 | 1 | 1 | 1 | 1 | 1 | 1 | 1 |
| smi_0053 | 1 | 1 | 1 | 1 | 1 | 1 | 1 | 1 | 1 | 1 | 1 |
| smi_0054 | 1 | 1 | 1 | 1 | 1 | 1 | 1 | 1 | 1 | 1 | 1 |
| smi_0055 | 1 | -1 | 1 | -1 | 1 | 1 | -1 | -1 | -1 | -1 | -1 |
| smi_0056 | 1 | -1 | 0 | -1 | -1 | 1 | -1 | -1 | -1 | -1 | -1 |
| smi_0057 | 1 | 1 | 1 | -1 | 1 | 1 | -1 | 1 | -1 | -1 | -1 |
| smi_0058 | 1 | 1 | 1 | -1 | 1 | 1 | -1 | -1 | -1 | -1 | -1 |
| smi_0059 | 1 | 1 | 1 | -1 | 1 | 1 | 0 | -1 | -1 | 0 | -1 |
| smi_0060 | 1 | 1 | -1 | -1 | -1 | -1 | -1 | -1 | -1 | -1 | -1 |
| smi_0061 | -1 | 1 | -1 | -1 | -1 | -1 | -1 | -1 | -1 | -1 | -1 |
| smi_0062 | -1 | 1 | -1 | -1 | -1 | -1 | -1 | 1 | -1 | -1 | -1 |
| smi_0063 | 1 | -1 | -1 | -1 | -1 | -1 | -1 | 1 | -1 | -1 | -1 |
| smi_0064 | 1 | 1 | 1 | 1 | 1 | 1 | 1 | 1 | 1 | 1 | 1 |
| smi_0065 | 1 | 1 | 1 | 1 | 1 | 1 | 1 | 1 | 1 | 1 | 1 |
| smi_0066 | 1 | 1 | 1 | 1 | 1 | 1 | 1 | 1 | 1 | 1 | 1 |
| smi_0067 | 1 | 1 | 1 | 1 | 1 | 1 | 1 | 1 | 1 | 1 | 1 |
| smi_0068 | 1 | 1 | 1 | 1 | 1 | 1 | 1 | 1 | 1 | 1 | 1 |
| smi_0069 | 1 | 1 | 1 | 1 | 1 | 1 | 1 | 1 | 1 | 1 | 1 |
| smi_0070 | 1 | 1 | 1 | 0 | -1 | 1 | 1 | 1 | 0 | 0 | 1 |
| smi_0071 | -1 | -1 | 1 | 1 | 1 | -1 | -1 | -1 | -1 | -1 | -1 |
| smi_0072 | 1 | 1 | 1 | 1 | 1 | 1 | 1 | 1 | 1 | 1 | 1 |
| smi_0073 | 1 | 1 | 1 | 1 | 1 | 1 | 1 | 1 | 1 | 0 | 1 |
| smi_0074 | 1 | 1 | 0 | 1 | 1 | 1 | 1 | 1 | 1 | 1 | 1 |
| smi_0075 | 1 | 1 | 1 | 1 | 1 | -1 | 1 | 1 | 1 | -1 | 0 |
| smi_0076 | 1 | 1 | 1 | 1 | 1 | 1 | 1 | 1 | 1 | 1 | 1 |
| smi_0077 | -1 | 0 | 1 | 1 | 1 | 1 | 1 | 0 | 1 | 1 | 1 |
| smi_0078 | 0 | 1 | 1 | 0 | 1 | 1 | 1 | -1 | 1 | 1 | -1 |
| smi_0079 | 1 | 1 | 1 | 1 | 1 | 1 | 1 | 1 | 1 | 1 | 1 |
| smi_0080 | 1 | 1 | 1 | 1 | 1 | 1 | 1 | 1 | 1 | 1 | 1 |
| smi_0081 | 1 | 1 | 1 | 1 | 1 | 1 | 1 | 1 | 1 | 1 | 1 |
| smi_0082 | 1 | 1 | 1 | 1 | 1 | 1 | 1 | 1 | 1 | 1 | 1 |
| smi_0083 | 1 | 1 | 1 | 1 | 1 | 1 | 1 | 1 | 1 | 1 | 1 |
| smi_0084 | 1 | 1 | 1 | 1 | 1 | 1 | 1 | 1 | 1 | 1 | 1 |
| smi_0085 | 1 | 1 | 1 | 1 | 1 | 1 | 1 | 1 | 1 | 1 | 1 |
| smi_0086 | 1 | 1 | 1 | -1 | 1 | 1 | -1 | -1 | -1 | -1 | -1 |
| smi_0087 | 1 | 1 | 1 | 1 | 1 | 0 | 1 | 1 | 1 | 1 | 1 |
| smi_0088 | 1 | 1 | 1 | 1 | -1 | 1 | 1 | -1 | 1 | 1 | -1 |
| smi_0089 | 1 | 1 | 1 | 1 | -1 | 1 | 1 | -1 | 1 | 1 | -1 |
| smi_0090 | 1 | 1 | -1 | 1 | 1 | 1 | 1 | 1 | -1 | -1 | 1 |
| smi_0091 | -1 | -1 | -1 | 0 | -1 | -1 | 0 | -1 | -1 | -1 | 1 |
| smi_0092 | 1 | 1 | 1 | 1 | 1 | 1 | 1 | -1 | 1 | 1 | 1 |
| smi_0093 | 1 | 1 | 1 | 1 | 1 | 1 | 1 | -1 | 1 | 1 | 1 |
| smi_0094 | 1 | 1 | 1 | 1 | 1 | 1 | 1 | 1 | 1 | 1 | 1 |
| smi_0095 | -1 | -1 | 1 | -1 | 1 | -1 | 1 | -1 | -1 | 1 | -1 |
| smi_0096 | -1 | -1 | -1 | -1 | 1 | -1 | -1 | 1 | -1 | -1 | -1 |
| smi_0097 | -1 | 1 | 1 | -1 | 1 | 1 | -1 | 1 | -1 | -1 | -1 |
| smi_0098 | -1 | -1 | 1 | -1 | 1 | 1 | -1 | 1 | -1 | -1 | -1 |
| smi_0099 | -1 | -1 | -1 | 1 | -1 | -1 | -1 | 1 | 1 | -1 | -1 |
| smi_0100 | -1 | -1 | -1 | 1 | -1 | -1 | -1 | 1 | 1 | -1 | -1 |
| smi_0101 | 1 | 1 | -1 | 1 | -1 | 1 | 1 | -1 | -1 | 1 | -1 |
| smi_0102 | 1 | 1 | -1 | 1 | -1 | 1 | 1 | -1 | -1 | 1 | -1 |
| smi_0103 | 1 | 1 | 1 | 1 | -1 | 1 | -1 | -1 | 1 | -1 | -1 |
| smi_0104 | 1 | 1 | 1 | 1 | -1 | 1 | -1 | -1 | 1 | -1 | -1 |
| smi_0105 | 1 | 1 | 1 | 1 | 1 | 1 | 1 | 1 | 1 | 1 | 1 |
| smi_0106 | 1 | 1 | 1 | 1 | 1 | 1 | 1 | 1 | 1 | 1 | 1 |
| smi_0107 | -1 | -1 | -1 | -1 | -1 | -1 | -1 | -1 | -1 | -1 | -1 |
| smi_0108 | 1 | -1 | -1 | -1 | 1 | -1 | -1 | -1 | -1 | -1 | 1 |
| smi_0109 | 1 | -1 | -1 | -1 | 1 | -1 | -1 | -1 | -1 | -1 | 1 |
| smi_0110 | 1 | -1 | -1 | -1 | 1 | -1 | -1 | -1 | -1 | -1 | 1 |
| smi_0111 | 1 | -1 | -1 | -1 | 1 | -1 | -1 | -1 | -1 | -1 | 1 |
| smi_0112 | 1 | -1 | -1 | -1 | 1 | -1 | -1 | -1 | -1 | -1 | 1 |
| smi_0113 | 1 | 1 | 1 | 1 | 1 | 1 | 1 | 1 | 1 | 1 | 1 |
| smi_0114 | 1 | 1 | 1 | 1 | 1 | 1 | 1 | 1 | 1 | 1 | 1 |
| smi_0115 | 1 | 1 | 1 | 1 | 1 | 1 | 1 | 1 | 1 | 1 | 1 |
| smi_0116 | 1 | 1 | 1 | 1 | 1 | 1 | -1 | 1 | -1 | -1 | 1 |
| smi_0117 | 1 | 1 | 1 | 1 | 1 | 1 | -1 | 1 | -1 | -1 | 1 |
| smi_0118 | 1 | 1 | 1 | 1 | 1 | 1 | 1 | 1 | 1 | 1 | 1 |
| smi_0119 | -1 | 1 | -1 | 1 | -1 | -1 | -1 | -1 | -1 | -1 | -1 |
| smi_0120 | 1 | 1 | 1 | 1 | 1 | 1 | 1 | 1 | 1 | 1 | 1 |
| smi_0121 | 1 | 1 | 1 | 1 | 1 | 1 | 1 | 1 | -1 | 1 | 1 |
| smi_0122 | 1 | 1 | -1 | 1 | 1 | -1 | 1 | -1 | 1 | 1 | 0 |
| smi_0123 | 1 | 1 | 1 | 1 | 1 | 1 | 1 | -1 | -1 | 1 | -1 |
| smi_0124 | 1 | 1 | 1 | 1 | 1 | 1 | 1 | -1 | -1 | 1 | -1 |
| smi_0125 | 1 | 1 | 1 | 1 | 1 | 1 | 1 | -1 | -1 | 1 | 1 |
| smi_0126 | 1 | 1 | 1 | 1 | 1 | 1 | 1 | 1 | 1 | 1 | 1 |
| smi_0127 | 1 | 1 | 1 | 1 | 1 | 1 | 1 | 1 | 1 | 1 | 1 |
| smi_0128 | 1 | 1 | 1 | 1 | 1 | 1 | 1 | 1 | 1 | 1 | 1 |
| smi_0129 | 1 | 1 | 1 | 1 | -1 | 1 | 1 | 0 | 1 | 1 | 0 |
| smi_0130 | 1 | 1 | 1 | 1 | 1 | 1 | 1 | 1 | 1 | 1 | 1 |
| smi_0131 | 1 | 1 | 1 | 1 | 1 | 1 | 1 | 1 | 1 | 1 | 1 |
| smi_0132 | 1 | 1 | 1 | 1 | 1 | 1 | 1 | 1 | 1 | 1 | 1 |
| smi_0133 | 1 | 1 | 1 | 1 | 1 | 1 | 1 | 1 | 1 | 1 | 1 |
| smi_0134 | 1 | 1 | 1 | 1 | 1 | 1 | 1 | 1 | 1 | 1 | 1 |
| smi_0135 | 1 | 1 | 1 | 1 | 1 | 1 | 1 | 1 | 1 | 1 | 1 |
| smi_0136 | 1 | 1 | 1 | 1 | 1 | 1 | 1 | 1 | 1 | 1 | 1 |
| smi_0137 | 1 | 1 | 1 | 1 | 1 | 1 | -1 | 1 | -1 | 1 | 1 |
| smi_0138 | 1 | 1 | 1 | 1 | 1 | 1 | -1 | 1 | -1 | 1 | 1 |
| smi_0139 | 1 | 1 | 1 | 1 | 1 | 1 | 1 | 1 | 1 | 1 | 1 |
| smi_0140 | -1 | -1 | -1 | -1 | -1 | -1 | -1 | -1 | -1 | -1 | -1 |
| smi_0141 | 1 | 1 | 1 | -1 | -1 | 1 | 1 | -1 | 1 | 1 | -1 |
| smi_0142 | 1 | 1 | 1 | 1 | 1 | 1 | 1 | 1 | 1 | 1 | 1 |
| smi_0143 | 1 | 1 | 1 | 1 | 1 | 1 | 1 | 1 | 1 | 1 | 1 |
| smi_0144 | 1 | 1 | 1 | 1 | 1 | 1 | 1 | 1 | 1 | 1 | 1 |
| smi_0145 | 1 | 1 | 1 | 1 | 1 | 1 | 1 | 1 | 1 | 1 | 1 |
| smi_0146 | 1 | 1 | 1 | 1 | 1 | 1 | 1 | 1 | 1 | 1 | 1 |
| smi_0147 | 1 | 1 | 1 | 1 | 1 | 1 | 1 | 1 | 1 | 1 | -1 |
| smi_0148 | 0 | 1 | 1 | 0 | 1 | 1 | 1 | 1 | 1 | 1 | 1 |
| smi_0149 | 1 | 1 | 1 | 1 | 1 | 1 | 1 | 1 | 1 | 1 | -1 |
| smi_0150 | 1 | 1 | 1 | 1 | 1 | 1 | 1 | 1 | 1 | 1 | 1 |
| smi_0151 | 1 | 1 | 1 | 1 | 1 | 1 | 1 | 1 | 1 | 1 | 1 |
| smi_0152 | 1 | 1 | 1 | 1 | 1 | 1 | 1 | 1 | 1 | 1 | 1 |
| smi_0153 | 1 | 1 | 1 | 1 | 1 | 1 | 1 | 1 | 1 | 1 | 1 |
| smi_0154 | 1 | 1 | 1 | 1 | 1 | 1 | 1 | 1 | 1 | 1 | 1 |
| smi_0155 | 1 | 1 | 1 | 1 | 1 | 1 | 1 | 1 | 1 | 1 | 1 |
| smi_0156 | -1 | -1 | 1 | -1 | -1 | -1 | 0 | 0 | -1 | -1 | 0 |
| smi_0157 | 1 | 1 | 1 | 0 | 1 | 0 | 1 | 1 | 1 | 1 | 0 |
| smi_0158 | 1 | 1 | 1 | 1 | 1 | 1 | 1 | 1 | 1 | 1 | 1 |
| smi_0159 | 1 | 1 | 1 | 1 | 1 | 1 | 1 | 1 | 1 | 1 | 1 |
| smi_0162 | 1 | 1 | 1 | 1 | 1 | 1 | 1 | 1 | 1 | 1 | 1 |
| smi_0165 | 1 | 1 | 1 | 1 | 1 | 1 | 1 | 1 | 1 | 1 | 1 |
| smi_0167 | 1 | 1 | 1 | 1 | 1 | 1 | 1 | 1 | 1 | 1 | 1 |
| smi_0169 | 1 | 1 | 1 | 1 | 1 | 1 | 1 | 1 | 1 | 1 | 1 |
| smi_0172 | 1 | 1 | 1 | 1 | 1 | 1 | 1 | 1 | 1 | 1 | 1 |
| smi_0173 | 1 | 1 | 1 | 1 | 1 | 1 | 1 | 1 | 1 | 1 | 1 |
| smi_0174 | 1 | 1 | 1 | 1 | 1 | 1 | 1 | 1 | 1 | 1 | 1 |
| smi_0175 | 1 | 1 | 1 | 1 | 1 | 1 | 1 | 1 | 1 | 1 | 1 |
| smi_0176 | 1 | 1 | 1 | 1 | 1 | 1 | 1 | 1 | 1 | 1 | 1 |
| smi_0177 | -1 | -1 | -1 | -1 | -1 | -1 | 1 | -1 | -1 | -1 | -1 |
| smi_0178 | -1 | -1 | -1 | -1 | -1 | -1 | 1 | -1 | -1 | -1 | -1 |
| smi_0179 | -1 | -1 | -1 | -1 | -1 | -1 | 1 | -1 | -1 | -1 | -1 |
| smi_0180 | -1 | -1 | -1 | -1 | -1 | -1 | 1 | -1 | -1 | -1 | -1 |
| smi_0181 | -1 | -1 | -1 | -1 | -1 | -1 | 1 | -1 | -1 | -1 | -1 |
| smi_0182 | 0 | -1 | -1 | -1 | -1 | -1 | 1 | -1 | -1 | -1 | -1 |
| smi_0183 | -1 | -1 | -1 | -1 | -1 | -1 | 1 | -1 | -1 | -1 | -1 |
| smi_0184 | -1 | -1 | -1 | -1 | -1 | -1 | 1 | -1 | -1 | -1 | -1 |
| smi_0185 | -1 | -1 | -1 | -1 | -1 | -1 | -1 | -1 | -1 | -1 | -1 |
| smi_0186 | 0 | -1 | -1 | -1 | -1 | -1 | 1 | -1 | -1 | -1 | -1 |
| smi_0187 | -1 | -1 | -1 | -1 | -1 | -1 | -1 | -1 | -1 | -1 | -1 |
| smi_0188 | -1 | -1 | -1 | -1 | -1 | -1 | -1 | -1 | -1 | -1 | -1 |
| smi_0189 | -1 | -1 | -1 | -1 | -1 | -1 | 1 | -1 | -1 | -1 | -1 |
| smi_0190 | -1 | -1 | -1 | -1 | -1 | -1 | 1 | -1 | -1 | -1 | -1 |
| smi_0191 | -1 | -1 | -1 | -1 | -1 | -1 | -1 | -1 | -1 | -1 | -1 |
| smi_0192 | 1 | 1 | 1 | 1 | 1 | 1 | 1 | 1 | 1 | 1 | 1 |
| smi_0193 | 1 | 1 | 1 | 1 | 1 | 1 | 1 | 1 | 1 | 1 | 1 |
| smi_0194 | 1 | 1 | 1 | 1 | 1 | 1 | 1 | 1 | 1 | 1 | 0 |
| smi_0195 | 1 | 1 | 1 | 1 | 1 | 1 | 1 | 1 | 1 | 1 | 1 |
| smi_0196 | 1 | 1 | 1 | 1 | 1 | 1 | 1 | 1 | 1 | 1 | 1 |
| smi_0197 | 1 | 1 | 1 | 1 | 1 | 1 | 1 | 1 | 1 | 1 | 1 |
| smi_0198 | 1 | -1 | -1 | -1 | -1 | -1 | -1 | -1 | -1 | -1 | 1 |
| smi_0199 | 1 | 1 | 1 | 1 | 1 | 1 | 1 | 1 | 1 | 1 | 1 |
| smi_0200 | 1 | 1 | 1 | 1 | 1 | 1 | 1 | 1 | 1 | 1 | 1 |
| smi_0202 | 1 | 1 | 1 | 1 | 1 | 1 | 1 | 1 | 1 | 1 | 1 |
| smi_0203 | 1 | 1 | 1 | 1 | 1 | 1 | 1 | 1 | 1 | 1 | 1 |
| smi_0204 | 1 | 1 | 1 | 1 | 1 | 1 | 1 | 1 | 1 | 1 | 1 |
| smi_0205 | 1 | 1 | 1 | 1 | 1 | 1 | 1 | 1 | 1 | 1 | 1 |
| smi_0206 | 1 | 1 | 1 | 1 | 1 | 1 | 1 | 1 | 1 | 1 | 1 |
| smi_0207 | 1 | 1 | 1 | 1 | 1 | 1 | 1 | 1 | 1 | 1 | 1 |
| smi_0208 | 1 | 1 | 1 | 1 | 1 | 1 | 1 | 1 | 1 | 1 | 1 |
| smi_0209 | 1 | 1 | 1 | 1 | 1 | 1 | 1 | 1 | 1 | 1 | 1 |
| smi_0210 | 1 | 1 | 1 | 1 | 1 | 1 | 1 | 1 | 1 | 1 | 1 |
| smi_0211 | 1 | 1 | 0 | 1 | 1 | 1 | 1 | 1 | 1 | 1 | 1 |
| smi_0212 | 1 | 1 | 1 | 1 | 1 | 1 | 1 | 1 | 1 | 1 | 1 |
| smi_0213 | 1 | 1 | 1 | 1 | 1 | 1 | 1 | 1 | 1 | 1 | 1 |
| smi_0214 | 1 | 1 | 1 | 1 | 1 | 1 | 1 | 1 | 1 | 1 | 1 |
| smi_0215 | 1 | 1 | 1 | 1 | 1 | 1 | 1 | 1 | 1 | 1 | 1 |
| smi_0216 | 1 | 1 | 1 | 1 | 1 | 1 | 1 | 1 | 1 | 1 | 1 |
| smi_0217 | 1 | 1 | 1 | 1 | 1 | 1 | 1 | 1 | 1 | 1 | -1 |
| smi_0218 | 1 | 0 | 1 | 0 | 1 | 1 | 1 | 1 | 0 | 1 | 1 |
| smi_0219 | -1 | -1 | -1 | 1 | -1 | -1 | -1 | -1 | -1 | -1 | 1 |
| smi_0220 | -1 | -1 | -1 | 1 | -1 | -1 | -1 | -1 | -1 | -1 | 1 |
| smi_0221 | 0 | 0 | -1 | 1 | -1 | -1 | -1 | -1 | -1 | -1 | 1 |
| smi_0222 | -1 | -1 | -1 | 1 | -1 | -1 | -1 | -1 | -1 | -1 | 1 |
| smi_0223 | -1 | -1 | -1 | 1 | -1 | -1 | -1 | -1 | -1 | -1 | 1 |
| smi_0224 | -1 | -1 | -1 | 1 | -1 | -1 | -1 | -1 | -1 | -1 | 1 |
| smi_0225 | -1 | -1 | -1 | 1 | -1 | -1 | -1 | -1 | -1 | -1 | 1 |
| smi_0226 | -1 | -1 | -1 | 1 | -1 | -1 | -1 | -1 | -1 | -1 | 1 |
| smi_0227 | 1 | 1 | 1 | 1 | 1 | 1 | 1 | 1 | 1 | 1 | 1 |
| smi_0228 | 1 | 1 | 1 | 1 | 1 | 1 | 1 | 1 | 1 | 1 | 1 |
| smi_0229 | 1 | 1 | 1 | 1 | 1 | 1 | 1 | 1 | 1 | 1 | 1 |
| smi_0230 | 1 | 1 | 1 | 1 | 1 | 1 | 1 | 1 | 1 | 1 | 1 |
| smi_0231 | 1 | 1 | 1 | 1 | 1 | 1 | 1 | 1 | 1 | 1 | 1 |
| smi_0232 | 1 | 1 | 1 | 1 | 1 | 1 | 1 | 1 | 1 | 1 | 1 |
| smi_0233 | 1 | 1 | 1 | 1 | 1 | 1 | 1 | 1 | 1 | 1 | 0 |
| smi_0234 | 1 | 1 | 1 | 1 | 1 | 1 | 1 | 1 | 1 | 1 | 1 |
| smi_0236 | 1 | 1 | 1 | 1 | 1 | 1 | 1 | 1 | 1 | 1 | 1 |
| smi_0237 | 1 | 1 | 1 | 1 | 1 | 1 | 1 | 1 | 1 | 1 | 1 |
| smi_0238 | 1 | 1 | 1 | 1 | 1 | 1 | 1 | 1 | 1 | 0 | 1 |
| smi_0239 | 1 | 1 | 0 | 1 | 1 | 1 | 1 | 1 | 1 | 1 | 0 |
| smi_0242 | 1 | 1 | 1 | 1 | 1 | 1 | 1 | 1 | 1 | 1 | 1 |
| smi_0243 | 1 | 1 | 1 | 1 | 1 | 1 | 1 | 1 | 1 | 1 | 1 |
| smi_0244 | 1 | 1 | 1 | 1 | 1 | 1 | 1 | 1 | 1 | 1 | 1 |
| smi_0246 | 1 | 1 | 1 | 1 | 1 | 1 | 1 | 1 | 1 | 1 | 1 |
| smi_0251 | 1 | 1 | 1 | 1 | 1 | 1 | 1 | 1 | 1 | 1 | 1 |
| smi_0252 | 1 | 1 | 1 | 1 | 1 | 1 | 1 | 1 | 1 | 1 | 1 |
| smi_0254 | 1 | 1 | 1 | 1 | 1 | 1 | 1 | 1 | 1 | 1 | 1 |
| smi_0255 | 1 | 1 | 1 | 1 | 1 | 1 | 1 | 1 | 1 | 1 | -1 |
| smi_0256 | 1 | 1 | 1 | 1 | 1 | 1 | 1 | 1 | 1 | 1 | 1 |
| smi_0257 | 1 | 1 | 1 | 1 | 1 | 1 | 1 | 1 | 1 | 1 | 1 |
| smi_0258 | 1 | 1 | 1 | 1 | 1 | 1 | 1 | 1 | 1 | 1 | 1 |
| smi_0259 | 1 | 1 | 1 | 1 | 1 | 1 | 1 | 1 | 1 | 1 | 1 |
| smi_0261 | 1 | 1 | 1 | 1 | 1 | 1 | 1 | 1 | 1 | 1 | 1 |
| smi_0262 | 1 | 1 | 1 | 1 | 1 | 1 | 1 | 1 | 1 | 1 | 1 |
| smi_0263 | 1 | 1 | 1 | 1 | 1 | 1 | 1 | 1 | 1 | 1 | 1 |
| smi_0264 | 1 | 1 | 1 | 1 | 1 | 1 | 1 | 1 | 1 | 1 | 1 |
| smi_0265 | -1 | -1 | -1 | 0 | -1 | -1 | -1 | -1 | -1 | -1 | 1 |
| smi_0266 | 1 | 1 | 1 | 1 | 1 | 1 | 1 | 1 | 1 | 1 | 1 |
| smi_0267 | 1 | 1 | 1 | 1 | 1 | 1 | 1 | 1 | 1 | 1 | 1 |
| smi_0268 | 1 | 1 | 1 | 1 | 1 | 1 | 1 | 1 | 1 | 1 | 1 |
| smi_0269 | 1 | 1 | 1 | -1 | -1 | -1 | -1 | -1 | -1 | -1 | -1 |
| smi_0270 | 1 | 1 | 1 | -1 | -1 | -1 | -1 | 0 | -1 | -1 | 0 |
| smi_0271 | 1 | 1 | 1 | -1 | -1 | 1 | -1 | -1 | -1 | -1 | -1 |
| smi_0272 | 1 | 1 | 1 | -1 | -1 | 1 | 1 | -1 | 1 | -1 | -1 |
| smi_0273 | 1 | 1 | 1 | 1 | 1 | 1 | 1 | 1 | 1 | 1 | 0 |
| smi_0274 | 1 | 1 | 1 | 1 | 1 | 1 | 1 | 1 | 1 | 1 | 1 |
| smi_0275 | 1 | 1 | 1 | 1 | 1 | 1 | 1 | 1 | 1 | 1 | 1 |
| smi_0276 | 1 | 1 | 1 | 1 | 1 | 1 | 1 | 1 | 1 | 1 | 1 |
| smi_0277 | 1 | 1 | 1 | 1 | 1 | 1 | 1 | 1 | 1 | 1 | 1 |
| smi_0278 | 1 | 1 | 1 | 1 | 1 | -1 | 1 | -1 | 1 | 1 | 1 |
| smi_0279 | 1 | 1 | 1 | 1 | 1 | 1 | 1 | 1 | 1 | 1 | 1 |
| smi_0280 | 1 | 1 | 1 | 1 | 1 | 1 | 1 | 1 | 1 | 1 | 1 |
| smi_0281 | 1 | 1 | 1 | 1 | 1 | 1 | 1 | 1 | 1 | 1 | 1 |
| smi_0282 | 1 | 1 | 1 | 1 | 1 | 1 | 1 | 1 | 1 | 1 | 1 |
| smi_0283 | 1 | 1 | 1 | 1 | 1 | 1 | 1 | 1 | 1 | 1 | 1 |
| smi_0284 | 1 | 1 | 1 | 1 | 0 | 1 | 1 | 1 | 1 | 1 | 0 |
| smi_0285 | 0 | 1 | 1 | 1 | 1 | 1 | 1 | 1 | 1 | 1 | 1 |
| smi_0286 | 1 | 1 | 1 | 1 | 1 | 1 | 1 | 1 | 1 | 1 | 1 |
| smi_0287 | 1 | 1 | 1 | 1 | 1 | 1 | 1 | 1 | 1 | 1 | 1 |
| smi_0288 | 1 | 1 | 1 | 1 | 1 | 1 | 1 | 1 | 1 | 1 | 1 |
| smi_0289 | 1 | 1 | 1 | 1 | 1 | 1 | 1 | 1 | 1 | 1 | 1 |
| smi_0290 | 1 | 1 | 1 | 1 | 1 | 1 | 1 | 1 | 1 | 1 | 1 |
| smi_0291 | 1 | 1 | 1 | 1 | 1 | 1 | 1 | -1 | 1 | 1 | -1 |
| smi_0292 | 1 | 1 | 1 | 1 | -1 | 1 | 1 | -1 | 1 | 1 | -1 |
| smi_0293 | 1 | 1 | 1 | 1 | 1 | 1 | 1 | 1 | 1 | 1 | 1 |
| smi_0294 | 1 | 1 | 1 | 1 | 1 | 1 | 1 | 1 | 1 | 1 | 1 |
| smi_0295 | 1 | -1 | 1 | 1 | 1 | 1 | 1 | 1 | 1 | 1 | 1 |
| smi_0296 | 1 | 1 | 1 | 1 | 1 | 1 | 1 | 1 | 1 | 1 | 1 |
| smi_0297 | 1 | 1 | 1 | 1 | 1 | 1 | 1 | 1 | 1 | 1 | 1 |
| smi_0298 | 1 | 1 | 1 | 1 | 1 | 0 | 1 | 1 | 1 | 1 | 1 |
| smi_0299 | 1 | 1 | 1 | 1 | 1 | 1 | 1 | 1 | 1 | 1 | 1 |
| smi_0300 | 1 | 1 | 1 | 1 | 1 | 1 | 1 | 1 | 1 | 1 | 1 |
| smi_0302 | 1 | 1 | 1 | 1 | 1 | 1 | 1 | 1 | 1 | 1 | 1 |
| smi_0303 | 1 | 1 | 1 | 1 | 1 | 1 | 1 | 1 | 1 | 1 | 1 |
| smi_0304 | 1 | 1 | 1 | 1 | 1 | 1 | 1 | 1 | 1 | 1 | 1 |
| smi_0305 | 0 | 1 | 1 | -1 | -1 | -1 | -1 | 1 | 1 | 1 | -1 |
| smi_0306 | -1 | 1 | 1 | -1 | -1 | -1 | -1 | 1 | 1 | 1 | -1 |
| smi_0307 | -1 | 1 | 1 | -1 | -1 | -1 | -1 | 1 | 1 | 1 | -1 |
| smi_0308 | -1 | 1 | 1 | -1 | -1 | -1 | -1 | 1 | 1 | 1 | -1 |
| smi_0309 | -1 | 1 | 1 | -1 | -1 | -1 | -1 | 1 | 1 | 1 | -1 |
| smi_0310 | 1 | 1 | 1 | 1 | 1 | 1 | 1 | 1 | 1 | 1 | 1 |
| smi_0311 | 1 | 1 | 1 | 1 | 1 | 1 | 1 | 1 | 1 | 1 | 1 |
| smi_0312 | 1 | 1 | 1 | 1 | 1 | 1 | 1 | 1 | 1 | 1 | 1 |
| smi_0313 | 1 | 1 | 1 | 1 | 1 | 1 | 1 | 1 | 1 | 1 | 1 |
| smi_0314 | 1 | 1 | 1 | 1 | 1 | 1 | 1 | 1 | 1 | 1 | 1 |
| smi_0315 | 1 | 1 | 1 | 1 | 1 | 1 | 1 | 1 | 1 | 1 | 1 |
| smi_0316 | 1 | 1 | 1 | 1 | 1 | 1 | 1 | 1 | 1 | 1 | 1 |
| smi_0317 | 1 | 1 | 1 | 1 | 1 | 1 | 1 | 1 | 1 | 1 | 1 |
| smi_0318 | 1 | 1 | 1 | 1 | 1 | 1 | 1 | 1 | 1 | 1 | 1 |
| smi_0319 | 1 | 0 | 1 | 1 | 1 | 1 | 1 | 1 | 1 | 1 | 1 |
| smi_0320 | 1 | 1 | 1 | 1 | 1 | 1 | 1 | -1 | 1 | 1 | -1 |
| smi_0321 | 1 | 1 | 1 | 1 | 1 | 1 | 1 | 1 | 1 | 1 | 1 |
| smi_0322 | -1 | 1 | -1 | -1 | -1 | -1 | -1 | -1 | -1 | -1 | -1 |
| smi_0323 | 1 | 1 | 1 | 1 | 1 | 1 | 1 | 1 | 1 | 1 | 1 |
| smi_0324 | 1 | 1 | 1 | 1 | 1 | 1 | 1 | 1 | 1 | 1 | 1 |
| smi_0325 | 1 | 1 | 1 | 1 | 1 | 1 | 1 | 1 | 1 | 1 | 1 |
| smi_0326 | 1 | 1 | 1 | 1 | 1 | 1 | 1 | 1 | 1 | 1 | 1 |
| smi_0327 | 1 | 1 | 1 | 1 | 1 | 1 | 1 | 1 | 1 | 1 | -1 |
| smi_0328 | 1 | 1 | 1 | 1 | 1 | 1 | 1 | 1 | 1 | 1 | -1 |
| smi_0329 | 1 | 1 | 1 | 1 | 1 | 1 | 1 | 1 | 1 | 1 | -1 |
| smi_0330 | 1 | 1 | -1 | -1 | -1 | 1 | -1 | 1 | -1 | -1 | 1 |
| smi_0331 | 1 | 1 | 1 | 1 | 1 | 1 | 1 | 1 | 1 | 1 | 1 |
| smi_0332 | 1 | 1 | 1 | 1 | 1 | 1 | 1 | 1 | 1 | 1 | 1 |
| smi_0333 | 1 | 1 | 1 | 1 | 1 | 1 | 1 | 1 | 1 | 1 | 1 |
| smi_0334 | 1 | 1 | 1 | 1 | 1 | 1 | 1 | 1 | 1 | 1 | 1 |
| smi_0335 | 1 | 1 | 1 | 1 | 1 | 1 | 1 | 1 | 1 | 1 | 1 |
| smi_0336 | 1 | 1 | 1 | 1 | 1 | 1 | 1 | 1 | 1 | 1 | 1 |
| smi_0337 | 1 | 1 | 1 | 1 | 1 | 1 | 1 | 1 | 1 | 1 | 1 |
| smi_0338 | 1 | 1 | 1 | 1 | 1 | 1 | 1 | 1 | 1 | 1 | 1 |
| smi_0339 | 1 | 1 | 1 | 1 | 1 | 1 | 1 | 1 | 1 | 1 | 1 |
| smi_0340 | 1 | 1 | 1 | 1 | 1 | 1 | 1 | 1 | 1 | 1 | 1 |
| smi_0341 | 1 | 1 | 1 | 1 | 1 | 1 | 1 | 1 | -1 | -1 | 1 |
| smi_0342 | 1 | 1 | 1 | 1 | 1 | 1 | 1 | 1 | 1 | 1 | 1 |
| smi_0343 | 1 | 1 | 1 | 1 | 1 | 1 | 1 | 1 | 1 | 1 | 1 |
| smi_0344 | 1 | -1 | 1 | 1 | 1 | 1 | 1 | -1 | 1 | 1 | 0 |
| smi_0345 | 1 | 1 | 1 | -1 | -1 | 1 | -1 | -1 | -1 | -1 | 1 |
| smi_0346 | 1 | 1 | 1 | 1 | 1 | 1 | 1 | 1 | 1 | 1 | 1 |
| smi_0347 | 1 | 1 | 1 | 1 | 1 | 1 | 1 | 1 | 1 | 1 | 1 |
| smi_0348 | 1 | 1 | 1 | 1 | 1 | 1 | 1 | 1 | 1 | 1 | 1 |
| smi_0349 | 1 | 1 | 1 | 1 | 1 | 1 | 1 | 1 | 1 | 1 | 1 |
| smi_0350 | 1 | 1 | 1 | 1 | 1 | 1 | 1 | 1 | 1 | 1 | -1 |
| smi_0351 | 1 | 1 | 1 | 1 | -1 | 1 | 1 | -1 | -1 | 1 | -1 |
| smi_0352 | 1 | 1 | 1 | 1 | 1 | 1 | 1 | 1 | 1 | 1 | 1 |
| smi_0353 | 1 | 1 | 1 | 1 | 1 | 1 | 1 | 1 | 1 | 1 | 1 |
| smi_0354 | 0 | 1 | 1 | 0 | 1 | 1 | 1 | 1 | 0 | 1 | 0 |
| smi_0355 | 1 | 1 | 1 | 1 | 1 | 1 | 1 | 1 | 1 | 1 | 1 |
| smi_0356 | 1 | 1 | 1 | -1 | 1 | -1 | 1 | 1 | -1 | -1 | -1 |
| smi_0357 | 1 | 1 | 1 | 1 | 1 | 1 | 1 | 1 | 1 | 1 | 1 |
| smi_0358 | 1 | 0 | 0 | -1 | -1 | -1 | 0 | -1 | -1 | -1 | -1 |
| smi_0359 | 1 | 1 | 1 | 1 | 1 | 1 | 1 | 1 | 1 | 1 | 1 |
| smi_0360 | 1 | 1 | 1 | 1 | 1 | 1 | 1 | 1 | 1 | 1 | 1 |
| smi_0361 | 1 | 1 | 1 | 1 | 1 | 0 | 1 | 1 | 1 | 0 | 0 |
| smi_0362 | 1 | 1 | 1 | 1 | 1 | 1 | 1 | 1 | 1 | 1 | 1 |
| smi_0363 | 1 | 1 | 1 | 1 | 1 | 1 | 1 | 1 | 1 | 1 | 1 |
| smi_0364 | -1 | -1 | 1 | -1 | 1 | 1 | 1 | -1 | -1 | -1 | 1 |
| smi_0365 | 1 | 1 | 1 | 1 | 1 | -1 | 1 | 1 | 1 | 1 | 1 |
| smi_0366 | 1 | 1 | 1 | 1 | 1 | 1 | 1 | 1 | 1 | 1 | 1 |
| smi_0367 | 1 | 1 | 1 | 1 | -1 | 1 | 1 | 1 | 1 | 1 | -1 |
| smi_0368 | 1 | 1 | 1 | 1 | -1 | -1 | -1 | 1 | 1 | -1 | -1 |
| smi_0369 | 1 | 1 | 1 | 1 | 0 | 1 | 1 | 1 | 1 | 1 | -1 |
| smi_0370 | 1 | 1 | -1 | 1 | -1 | -1 | -1 | 1 | -1 | -1 | -1 |
| smi_0371 | 1 | 1 | 1 | 0 | -1 | 0 | 1 | 0 | 1 | 1 | 1 |
| smi_0372 | 1 | 1 | 1 | 1 | 1 | 1 | 1 | 1 | 1 | 1 | 1 |
| smi_0373 | 1 | 1 | 1 | 1 | 1 | 1 | 1 | 1 | 1 | 1 | 1 |
| smi_0374 | 1 | 1 | 1 | 1 | 1 | 1 | 1 | 1 | 1 | 1 | 1 |
| smi_0375 | 1 | 1 | 1 | 1 | 1 | 1 | 1 | 1 | 1 | 1 | 1 |
| smi_0376 | 1 | 1 | 1 | 1 | 1 | 1 | 1 | 1 | 1 | 1 | 1 |
| smi_0377 | 1 | 1 | 1 | 1 | 1 | 1 | 1 | 1 | 1 | 1 | 1 |
| smi_0378 | 1 | 1 | 1 | 1 | 1 | 1 | 1 | 1 | 1 | 1 | 1 |
| smi_0379 | 1 | 1 | 1 | 1 | 1 | 1 | 1 | 1 | 1 | 1 | 1 |
| smi_0380 | 1 | 1 | 1 | 1 | 1 | 1 | 1 | 1 | 1 | 1 | 1 |
| smi_0381 | 1 | 1 | 1 | 1 | 1 | 1 | 1 | 1 | 1 | 1 | 1 |
| smi_0382 | 1 | 1 | 1 | 1 | 1 | 0 | 1 | 1 | 1 | 1 | 1 |
| smi_0383 | 1 | 1 | 1 | 1 | 1 | 1 | 1 | 1 | 1 | 1 | 1 |
| smi_0384 | 1 | 1 | 1 | 1 | 1 | 1 | 1 | 1 | 1 | 1 | 1 |
| smi_0385 | 1 | 1 | 1 | 1 | 1 | 1 | 1 | 1 | 1 | 1 | 1 |
| smi_0386 | 1 | 0 | 1 | 0 | -1 | -1 | 0 | 0 | 1 | -1 | -1 |
| smi_0387 | 1 | 1 | 1 | 1 | 1 | -1 | 1 | 1 | -1 | -1 | 1 |
| smi_0388 | 1 | -1 | 1 | 1 | 1 | 1 | 1 | -1 | 1 | 1 | -1 |
| smi_0389 | 1 | 1 | 1 | 1 | 1 | 1 | 1 | 1 | 1 | 1 | -1 |
| smi_0390 | 1 | -1 | -1 | -1 | -1 | -1 | -1 | -1 | 1 | -1 | -1 |
| smi_0391 | 1 | -1 | -1 | -1 | -1 | -1 | -1 | -1 | -1 | -1 | -1 |
| smi_0392 | 1 | -1 | -1 | -1 | -1 | -1 | -1 | -1 | -1 | -1 | -1 |
| smi_0393 | 1 | -1 | -1 | -1 | -1 | -1 | -1 | -1 | -1 | -1 | 1 |
| smi_0394 | 1 | -1 | -1 | -1 | -1 | -1 | -1 | 1 | -1 | -1 | 1 |
| smi_0395 | 1 | 1 | 1 | -1 | -1 | -1 | -1 | 1 | 1 | -1 | 1 |
| smi_0396 | 1 | -1 | -1 | -1 | -1 | -1 | -1 | -1 | -1 | -1 | -1 |
| smi_0397 | 1 | -1 | -1 | -1 | -1 | -1 | -1 | -1 | -1 | -1 | -1 |
| smi_0398 | 1 | -1 | -1 | -1 | -1 | -1 | -1 | -1 | -1 | -1 | -1 |
| smi_0399 | -1 | -1 | -1 | -1 | -1 | -1 | -1 | -1 | -1 | -1 | -1 |
| smi_0400 | 1 | 1 | 1 | 1 | 1 | 1 | 1 | 1 | 1 | 1 | 1 |
| smi_0401 | 1 | 1 | 1 | 1 | 1 | 1 | 1 | 1 | 1 | 1 | 1 |
| smi_0402 | 1 | 1 | 1 | 1 | 1 | 1 | 1 | 1 | 1 | 1 | -1 |
| smi_0403 | 1 | 1 | 1 | 1 | 1 | 1 | 1 | 1 | 1 | 1 | 1 |
| smi_0404 | 1 | 1 | 1 | 1 | 1 | 1 | 1 | 1 | 1 | 1 | 1 |
| smi_0405 | 1 | 1 | 1 | 1 | 1 | 1 | 1 | 1 | 1 | 1 | 1 |
| smi_0406 | 1 | 0 | 1 | 1 | 1 | 1 | 1 | 1 | 1 | 1 | 1 |
| smi_0407 | -1 | -1 | -1 | -1 | -1 | -1 | -1 | -1 | -1 | -1 | -1 |
| smi_0408 | -1 | -1 | -1 | -1 | -1 | -1 | -1 | -1 | -1 | -1 | -1 |
| smi_0409 | -1 | -1 | -1 | -1 | -1 | -1 | -1 | -1 | -1 | -1 | -1 |
| smi_0410 | -1 | -1 | -1 | -1 | -1 | -1 | -1 | -1 | -1 | -1 | -1 |
| smi_0411 | -1 | -1 | -1 | -1 | -1 | -1 | -1 | -1 | -1 | -1 | -1 |
| smi_0412 | -1 | 0 | -1 | -1 | -1 | -1 | -1 | -1 | -1 | -1 | -1 |
| smi_0413 | -1 | -1 | -1 | -1 | -1 | -1 | -1 | -1 | -1 | -1 | -1 |
| smi_0414 | -1 | -1 | -1 | -1 | -1 | -1 | -1 | -1 | -1 | -1 | -1 |
| smi_0415 | -1 | -1 | -1 | -1 | -1 | -1 | -1 | -1 | -1 | -1 | -1 |
| smi_0416 | -1 | -1 | -1 | -1 | -1 | -1 | -1 | -1 | -1 | -1 | -1 |
| smi_0417 | 0 | 1 | -1 | -1 | -1 | -1 | -1 | 1 | -1 | -1 | -1 |
| smi_0418 | -1 | -1 | -1 | -1 | -1 | -1 | -1 | -1 | -1 | -1 | -1 |
| smi_0419 | -1 | -1 | -1 | -1 | -1 | -1 | -1 | -1 | -1 | -1 | -1 |
| smi_0420 | -1 | -1 | -1 | -1 | -1 | -1 | -1 | -1 | -1 | -1 | -1 |
| smi_0421 | -1 | -1 | -1 | -1 | -1 | -1 | -1 | -1 | -1 | -1 | -1 |
| smi_0422 | -1 | -1 | -1 | -1 | -1 | -1 | -1 | -1 | -1 | -1 | -1 |
| smi_0423 | -1 | -1 | -1 | -1 | -1 | -1 | -1 | -1 | -1 | -1 | -1 |
| smi_0424 | -1 | -1 | -1 | -1 | -1 | -1 | -1 | -1 | -1 | -1 | -1 |
| smi_0425 | -1 | -1 | -1 | -1 | -1 | -1 | -1 | -1 | -1 | -1 | -1 |
| smi_0426 | -1 | -1 | -1 | -1 | -1 | -1 | -1 | -1 | -1 | -1 | -1 |
| smi_0427 | -1 | 1 | -1 | -1 | -1 | -1 | -1 | 1 | -1 | -1 | -1 |
| smi_0428 | 0 | 1 | -1 | -1 | -1 | -1 | 0 | -1 | -1 | -1 | -1 |
| smi_0429 | -1 | -1 | -1 | -1 | -1 | -1 | 1 | -1 | -1 | -1 | -1 |
| smi_0430 | -1 | -1 | -1 | -1 | -1 | -1 | -1 | -1 | -1 | -1 | -1 |
| smi_0431 | -1 | -1 | -1 | -1 | -1 | -1 | -1 | -1 | -1 | -1 | -1 |
| smi_0432 | -1 | -1 | -1 | -1 | -1 | -1 | -1 | -1 | -1 | -1 | -1 |
| smi_0433 | -1 | 1 | -1 | -1 | -1 | -1 | -1 | 0 | -1 | -1 | -1 |
| smi_0434 | -1 | 1 | -1 | -1 | -1 | -1 | -1 | -1 | -1 | -1 | -1 |
| smi_0435 | -1 | 1 | -1 | -1 | -1 | -1 | -1 | -1 | -1 | -1 | -1 |
| smi_0436 | -1 | -1 | -1 | -1 | -1 | -1 | -1 | -1 | -1 | -1 | -1 |
| smi_0437 | 0 | -1 | -1 | -1 | -1 | -1 | -1 | -1 | -1 | -1 | -1 |
| smi_0438 | 0 | -1 | -1 | -1 | -1 | -1 | -1 | 1 | -1 | -1 | -1 |
| smi_0439 | -1 | -1 | -1 | -1 | -1 | -1 | -1 | 1 | -1 | -1 | -1 |
| smi_0440 | -1 | -1 | -1 | -1 | -1 | -1 | -1 | -1 | -1 | -1 | -1 |
| smi_0441 | 1 | -1 | 0 | 1 | -1 | -1 | -1 | -1 | -1 | -1 | -1 |
| smi_0442 | -1 | -1 | -1 | -1 | -1 | -1 | -1 | 1 | -1 | -1 | -1 |
| smi_0443 | -1 | -1 | -1 | -1 | 0 | -1 | -1 | 1 | -1 | -1 | -1 |
| smi_0444 | -1 | -1 | -1 | -1 | -1 | -1 | -1 | 1 | -1 | -1 | -1 |
| smi_0445 | -1 | -1 | -1 | -1 | -1 | -1 | -1 | -1 | -1 | -1 | -1 |
| smi_0446 | -1 | -1 | -1 | -1 | -1 | -1 | -1 | -1 | -1 | -1 | -1 |
| smi_0447 | 0 | -1 | -1 | -1 | -1 | -1 | -1 | -1 | -1 | -1 | -1 |
| smi_0448 | -1 | -1 | -1 | -1 | -1 | -1 | -1 | -1 | -1 | -1 | -1 |
| smi_0449 | -1 | -1 | -1 | -1 | -1 | -1 | -1 | -1 | -1 | -1 | -1 |
| smi_0450 | -1 | -1 | -1 | -1 | -1 | -1 | -1 | -1 | -1 | -1 | -1 |
| smi_0451 | -1 | -1 | -1 | -1 | -1 | -1 | -1 | 1 | -1 | -1 | -1 |
| smi_0452 | -1 | -1 | -1 | -1 | -1 | -1 | -1 | 1 | -1 | -1 | -1 |
| smi_0453 | -1 | -1 | -1 | -1 | -1 | -1 | -1 | 1 | -1 | -1 | -1 |
| smi_0454 | -1 | -1 | -1 | -1 | -1 | -1 | -1 | 1 | -1 | -1 | -1 |
| smi_0455 | -1 | -1 | -1 | -1 | -1 | -1 | -1 | 1 | -1 | -1 | -1 |
| smi_0456 | -1 | -1 | -1 | -1 | -1 | -1 | -1 | 1 | -1 | -1 | -1 |
| smi_0457 | -1 | -1 | -1 | -1 | -1 | -1 | -1 | 1 | -1 | -1 | -1 |
| smi_0458 | -1 | -1 | -1 | -1 | -1 | -1 | -1 | 1 | -1 | -1 | -1 |
| smi_0459 | -1 | -1 | -1 | -1 | -1 | -1 | -1 | 1 | -1 | -1 | -1 |
| smi_0460 | -1 | -1 | -1 | -1 | -1 | -1 | -1 | 1 | -1 | -1 | -1 |
| smi_0461 | -1 | -1 | -1 | -1 | -1 | -1 | -1 | 1 | -1 | -1 | -1 |
| smi_0462 | -1 | -1 | -1 | -1 | -1 | -1 | -1 | 1 | -1 | -1 | -1 |
| smi_0463 | -1 | -1 | -1 | -1 | -1 | -1 | -1 | 1 | -1 | -1 | -1 |
| smi_0464 | -1 | -1 | -1 | -1 | -1 | -1 | -1 | 1 | -1 | -1 | -1 |
| smi_0465 | -1 | -1 | -1 | -1 | -1 | -1 | -1 | 1 | -1 | -1 | -1 |
| smi_0466 | -1 | -1 | -1 | -1 | -1 | -1 | -1 | 1 | -1 | -1 | -1 |
| smi_0467 | -1 | -1 | -1 | -1 | -1 | -1 | -1 | 1 | -1 | -1 | -1 |
| smi_0468 | -1 | -1 | -1 | -1 | -1 | -1 | -1 | 1 | -1 | -1 | -1 |
| smi_0469 | -1 | -1 | -1 | -1 | -1 | -1 | -1 | 1 | -1 | -1 | -1 |
| smi_0470 | -1 | 1 | -1 | -1 | -1 | -1 | -1 | 1 | -1 | -1 | -1 |
| smi_0471 | -1 | -1 | -1 | -1 | -1 | -1 | -1 | -1 | -1 | -1 | -1 |
| smi_0472 | 0 | 1 | -1 | -1 | -1 | -1 | -1 | 1 | -1 | -1 | -1 |
| smi_0473 | -1 | 0 | -1 | -1 | -1 | -1 | -1 | -1 | -1 | -1 | -1 |
| smi_0474 | 0 | 1 | 0 | -1 | -1 | -1 | 0 | -1 | -1 | -1 | -1 |
| smi_0475 | -1 | 1 | -1 | -1 | -1 | -1 | -1 | 1 | -1 | -1 | -1 |
| smi_0476 | -1 | 0 | -1 | -1 | -1 | -1 | -1 | -1 | -1 | -1 | -1 |
| smi_0477 | -1 | 1 | -1 | -1 | -1 | -1 | -1 | 1 | -1 | -1 | -1 |
| smi_0478 | -1 | 1 | -1 | -1 | 1 | -1 | -1 | 1 | -1 | 1 | 1 |
| smi_0479 | 1 | 1 | 0 | -1 | -1 | -1 | 0 | 0 | -1 | -1 | -1 |
| smi_0480 | 1 | 1 | 1 | 1 | 1 | 1 | 1 | 1 | 1 | 1 | 1 |
| smi_0481 | 1 | 1 | 1 | 1 | 1 | 1 | 1 | 1 | 1 | 1 | 1 |
| smi_0482 | 1 | 1 | 1 | 1 | 1 | 1 | 1 | 1 | 1 | 1 | 1 |
| smi_0483 | 1 | 1 | 1 | 1 | 1 | 1 | 1 | 1 | 1 | 1 | -1 |
| smi_0484 | 1 | 1 | -1 | 1 | 1 | -1 | -1 | 1 | -1 | -1 | -1 |
| smi_0485 | 1 | 1 | -1 | -1 | 1 | -1 | -1 | -1 | -1 | -1 | -1 |
| smi_0486 | 1 | 1 | 1 | 1 | 1 | 1 | 1 | 1 | 1 | 1 | 1 |
| smi_0487 | 1 | 1 | 1 | 1 | 1 | 1 | 1 | 1 | 1 | 1 | 1 |
| smi_0488 | 1 | 1 | 1 | 1 | 1 | 1 | 1 | 1 | 1 | 1 | 1 |
| smi_0490 | 1 | 1 | 1 | 1 | 0 | 1 | 1 | 1 | 1 | 0 | 1 |
| smi_0491 | 1 | 1 | 1 | 1 | 1 | 1 | 1 | 1 | 1 | 1 | 1 |
| smi_0496 | 1 | 1 | 1 | 1 | 1 | 1 | 1 | 1 | 1 | 1 | 1 |
| smi_0497 | 1 | 1 | 1 | 1 | 1 | 1 | 1 | 1 | 1 | 1 | 1 |
| smi_0498 | 1 | 1 | 1 | 1 | 1 | 1 | 1 | 1 | 1 | 1 | 1 |
| smi_0499 | 1 | 1 | 1 | 1 | 1 | 1 | 1 | 1 | 1 | 1 | 1 |
| smi_0502 | 1 | 1 | 1 | 1 | 1 | 1 | 1 | 1 | 1 | 1 | 1 |
| smi_0503 | 1 | 1 | 1 | 1 | 1 | 1 | 1 | 1 | 1 | 1 | 1 |
| smi_0504 | 1 | 1 | 1 | 1 | 1 | 1 | 1 | 1 | 1 | 1 | 1 |
| smi_0506 | 1 | 1 | 1 | 1 | 1 | 1 | 1 | 1 | 1 | 1 | 1 |
| smi_0507 | 1 | 1 | 1 | 1 | 1 | 1 | 1 | 1 | 1 | 1 | 1 |
| smi_0508 | 1 | 1 | 1 | 1 | 1 | 1 | 1 | 1 | 1 | 1 | 1 |
| smi_0510 | 1 | 1 | 1 | 1 | 1 | 1 | 1 | 1 | 1 | 1 | 1 |
| smi_0511 | 1 | 1 | 1 | 1 | 1 | -1 | 1 | 1 | 1 | 1 | 1 |
| smi_0512 | -1 | -1 | -1 | -1 | -1 | -1 | -1 | -1 | -1 | -1 | -1 |
| smi_0513 | 1 | 1 | 1 | 1 | 1 | 1 | 1 | 1 | 1 | 1 | 1 |
| smi_0514 | 1 | 1 | 1 | 1 | 1 | 1 | 1 | 1 | 1 | 1 | 1 |
| smi_0515 | 1 | 1 | 1 | 1 | 1 | 1 | 1 | 1 | 1 | 1 | 1 |
| smi_0516 | 1 | 1 | 1 | 1 | 1 | 1 | 1 | 1 | 1 | 1 | 1 |
| smi_0517 | 0 | 1 | -1 | -1 | -1 | -1 | -1 | -1 | -1 | -1 | -1 |
| smi_0518 | 1 | 1 | 1 | 1 | 1 | 1 | -1 | 1 | -1 | 1 | 1 |
| smi_0519 | 1 | 1 | 1 | 1 | 1 | 1 | 1 | 1 | 1 | 1 | 1 |
| smi_0520 | 1 | 1 | 1 | 1 | 1 | 1 | 1 | 1 | 1 | 1 | 1 |
| smi_0521 | 1 | 1 | 1 | 1 | 1 | 1 | 1 | 1 | 1 | 1 | 1 |
| smi_0522 | 1 | 1 | 1 | 1 | 1 | 1 | 1 | 1 | 1 | 1 | 1 |
| smi_0523 | -1 | -1 | -1 | -1 | -1 | -1 | -1 | -1 | -1 | -1 | -1 |
| smi_0524 | -1 | -1 | -1 | -1 | -1 | -1 | -1 | -1 | -1 | -1 | -1 |
| smi_0525 | -1 | -1 | -1 | -1 | -1 | -1 | -1 | -1 | -1 | -1 | -1 |
| smi_0526 | -1 | -1 | -1 | -1 | -1 | -1 | -1 | -1 | -1 | -1 | -1 |
| smi_0527 | 1 | 1 | 1 | 1 | 1 | 1 | 1 | 1 | 1 | 1 | 1 |
| smi_0528 | 1 | 1 | 1 | 1 | 1 | 1 | 1 | 1 | 1 | 1 | 1 |
| smi_0529 | -1 | -1 | -1 | 1 | 1 | 1 | 1 | 1 | 1 | 1 | -1 |
| smi_0530 | 1 | 1 | 1 | 1 | 1 | 1 | 1 | 1 | 1 | 1 | 1 |
| smi_0531 | -1 | 1 | 1 | 1 | -1 | -1 | -1 | -1 | -1 | -1 | -1 |
| smi_0532 | -1 | 1 | 1 | 1 | -1 | -1 | -1 | -1 | -1 | -1 | -1 |
| smi_0533 | -1 | 1 | 1 | 1 | -1 | -1 | -1 | -1 | -1 | -1 | -1 |
| smi_0534 | 1 | 1 | 1 | 1 | 1 | 1 | 1 | 1 | 1 | 1 | -1 |
| smi_0535 | 1 | 1 | 1 | 1 | 1 | 1 | 1 | 1 | 1 | 1 | 1 |
| smi_0536 | 1 | 1 | 1 | 1 | 1 | 1 | 1 | 1 | 1 | 1 | 1 |
| smi_0537 | 1 | 1 | 1 | 1 | 1 | 1 | 1 | 1 | 1 | 1 | 1 |
| smi_0538 | 1 | 1 | 1 | 1 | 1 | 1 | 1 | 1 | 1 | 1 | 1 |
| smi_0539 | 1 | 1 | 1 | 1 | 1 | 1 | 1 | 1 | 1 | 1 | 1 |
| smi_0540 | 1 | 1 | 1 | 1 | 1 | 1 | 1 | 1 | 1 | 1 | 1 |
| smi_0541 | 1 | 1 | 1 | 1 | 1 | 1 | 1 | 1 | 1 | 1 | 1 |
| smi_0542 | 1 | 1 | 1 | 1 | 1 | 1 | 1 | 1 | 1 | 1 | 1 |
| smi_0543 | 1 | 1 | 1 | 1 | 1 | 1 | 1 | 1 | 1 | 1 | 1 |
| smi_0544 | 1 | 1 | 1 | 1 | 1 | 1 | 1 | 1 | 1 | 1 | 1 |
| smi_0545 | 1 | 1 | 1 | 1 | 1 | 1 | 1 | 1 | 1 | 1 | 1 |
| smi_0546 | 1 | 1 | 1 | 1 | 1 | 1 | 1 | 1 | 1 | 1 | 1 |
| smi_0547 | 1 | 1 | 1 | 1 | 1 | 1 | 1 | 1 | 1 | 1 | 1 |
| smi_0548 | 1 | 1 | 1 | 1 | 1 | 1 | 1 | 1 | 1 | 1 | 1 |
| smi_0549 | 1 | 1 | 1 | 1 | 1 | 0 | 1 | 0 | 1 | 1 | 1 |
| smi_0550 | 1 | 1 | -1 | 1 | -1 | 1 | 1 | -1 | 1 | 1 | 1 |
| smi_0551 | 1 | 1 | -1 | 1 | -1 | 1 | 1 | -1 | 1 | 1 | 1 |
| smi_0552 | 1 | 1 | -1 | 1 | -1 | -1 | 1 | -1 | 1 | 1 | 1 |
| smi_0553 | 1 | 1 | -1 | 1 | -1 | 1 | 1 | -1 | 1 | 1 | 1 |
| smi_0554 | 1 | 1 | -1 | 1 | -1 | 1 | 1 | -1 | 1 | 1 | 1 |
| smi_0555 | 1 | 1 | -1 | 1 | -1 | 1 | 1 | -1 | 1 | 1 | 1 |
| smi_0556 | -1 | 1 | -1 | 1 | -1 | -1 | 1 | -1 | -1 | -1 | -1 |
| smi_0557 | 1 | 1 | 0 | 1 | -1 | 0 | 1 | 1 | 1 | 1 | 0 |
| smi_0558 | 1 | 1 | -1 | 1 | 0 | -1 | 1 | 1 | 1 | 1 | -1 |
| smi_0559 | 1 | 1 | 1 | 1 | 1 | 1 | 1 | 1 | 1 | 1 | 1 |
| smi_0560 | 1 | 1 | 0 | 1 | 1 | 1 | 1 | 1 | 1 | 1 | 1 |
| smi_0561 | 1 | 1 | 1 | 1 | 1 | 1 | 1 | 1 | 1 | 1 | 1 |
| smi_0562 | 1 | 1 | 1 | 1 | -1 | -1 | -1 | -1 | -1 | -1 | 1 |
| smi_0563 | 1 | 1 | 1 | 1 | 1 | 1 | 1 | 1 | 1 | 1 | 1 |
| smi_0564 | 0 | 1 | 1 | 1 | 1 | 1 | 1 | 1 | 1 | 1 | 1 |
| smi_0565 | 1 | 1 | 1 | 1 | 1 | 1 | 1 | 1 | 1 | 1 | 1 |
| smi_0566 | 1 | 1 | 1 | 1 | 1 | 1 | -1 | 1 | -1 | -1 | 1 |
| smi_0567 | 1 | 1 | 1 | 1 | -1 | 1 | -1 | -1 | -1 | -1 | -1 |
| smi_0568 | 1 | 1 | 1 | 1 | 1 | 1 | 1 | 1 | 1 | 1 | 1 |
| smi_0569 | 1 | 1 | 1 | 1 | 1 | 1 | 1 | 1 | 1 | 1 | 1 |
| smi_0570 | 1 | 1 | 1 | 1 | 1 | 1 | 1 | 1 | 1 | 1 | -1 |
| smi_0571 | 1 | 1 | 1 | 1 | 1 | 1 | 1 | 1 | 1 | 1 | -1 |
| smi_0572 | 1 | 1 | 1 | 1 | 1 | 1 | 1 | 1 | 1 | 1 | -1 |
| smi_0573 | 1 | 1 | 1 | 1 | 1 | 1 | 1 | 1 | 1 | 1 | 1 |
| smi_0574 | 1 | 1 | 1 | 1 | 1 | 1 | 1 | 1 | 1 | 1 | 1 |
| smi_0575 | 1 | 1 | 1 | 1 | 1 | 1 | 1 | 1 | 1 | 1 | 1 |
| smi_0576 | 1 | 1 | 1 | 1 | 1 | 1 | 1 | 1 | 1 | 1 | -1 |
| smi_0577 | 1 | 1 | 1 | 1 | 1 | 1 | 1 | 1 | 1 | 1 | -1 |
| smi_0578 | 1 | 1 | 1 | 1 | 1 | -1 | -1 | 1 | -1 | -1 | -1 |
| smi_0579 | 1 | 1 | 1 | 1 | 1 | 1 | 1 | 1 | 1 | 1 | 1 |
| smi_0580 | 1 | 1 | 1 | 1 | 1 | 1 | 1 | 1 | 1 | 1 | 1 |
| smi_0581 | 1 | 1 | 1 | 1 | 1 | 1 | 1 | 1 | 1 | 1 | 1 |
| smi_0582 | 0 | 1 | 1 | 1 | 1 | 1 | 1 | 1 | 1 | 1 | 1 |
| smi_0583 | 1 | 1 | 1 | 1 | 1 | 1 | 1 | 1 | 1 | 1 | 1 |
| smi_0584 | 1 | 1 | 1 | 1 | 1 | 1 | 1 | 1 | 1 | 1 | 1 |
| smi_0585 | 1 | 1 | 1 | 1 | -1 | 0 | 1 | 1 | 1 | 1 | 1 |
| smi_0586 | 1 | 1 | 1 | 1 | 1 | 1 | 1 | 1 | 1 | 1 | 1 |
| smi_0587 | 1 | 1 | -1 | 1 | 1 | -1 | 1 | 1 | 1 | 1 | 1 |
| smi_0588 | 1 | 1 | 1 | 1 | 1 | 1 | 1 | 1 | 1 | 1 | 1 |
| smi_0589 | 1 | 1 | 1 | 1 | 1 | 1 | 1 | 1 | 1 | 1 | 1 |
| smi_0590 | 1 | 1 | 1 | 1 | 1 | 1 | 1 | 1 | 1 | 1 | 1 |
| smi_0591 | 1 | 1 | 1 | 1 | 1 | 1 | 1 | 1 | 1 | 1 | 1 |
| smi_0592 | 1 | 1 | 1 | 1 | 1 | 1 | 1 | 1 | 1 | 1 | 1 |
| smi_0593 | 1 | 1 | 1 | 1 | 1 | 1 | 1 | 1 | 1 | 1 | 1 |
| smi_0594 | 1 | 1 | 1 | 1 | 1 | 1 | 1 | 1 | 1 | 1 | 1 |
| smi_0595 | 1 | 1 | 1 | 1 | 1 | 1 | 1 | 1 | 1 | 1 | 1 |
| smi_0596 | -1 | -1 | -1 | -1 | 0 | -1 | -1 | -1 | -1 | -1 | -1 |
| smi_0597 | 0 | -1 | -1 | 1 | -1 | -1 | -1 | -1 | -1 | -1 | 0 |
| smi_0598 | 1 | 1 | 0 | 1 | 1 | 1 | -1 | 1 | -1 | -1 | -1 |
| smi_0599 | 1 | 1 | -1 | 1 | 1 | -1 | -1 | -1 | -1 | 1 | -1 |
| smi_0600 | 1 | 1 | 1 | 1 | 1 | 1 | 1 | 1 | 1 | 1 | 1 |
| smi_0601 | 1 | -1 | 1 | -1 | -1 | 0 | -1 | -1 | -1 | -1 | -1 |
| smi_0602 | 1 | 1 | 1 | 1 | 1 | 1 | 1 | 1 | 1 | 1 | 1 |
| smi_0603 | 1 | -1 | 1 | -1 | 1 | 1 | 1 | 1 | 1 | 1 | 1 |
| smi_0604 | 1 | -1 | 1 | -1 | 0 | 1 | 1 | 1 | -1 | 1 | -1 |
| smi_0605 | 1 | -1 | 1 | -1 | 1 | 1 | 1 | 1 | 1 | 1 | 1 |
| smi_0606 | 1 | -1 | 1 | -1 | 1 | 1 | 1 | 1 | 1 | 1 | 1 |
| smi_0607 | 1 | -1 | -1 | -1 | -1 | 1 | 1 | -1 | 1 | 1 | -1 |
| smi_0608 | 1 | 1 | 1 | 1 | 1 | 1 | 1 | 1 | 1 | 1 | 1 |
| smi_0609 | 0 | -1 | 1 | 0 | 0 | -1 | 0 | -1 | 0 | -1 | 0 |
| smi_0610 | -1 | 1 | -1 | 1 | 1 | -1 | 1 | 1 | -1 | -1 | -1 |
| smi_0611 | -1 | 1 | 0 | 1 | 1 | -1 | 1 | 1 | -1 | -1 | -1 |
| smi_0612 | 1 | 1 | 1 | 1 | 1 | 1 | 1 | 1 | 1 | 1 | 1 |
| smi_0613 | 1 | 1 | -1 | -1 | 1 | -1 | -1 | -1 | 1 | -1 | -1 |
| smi_0614 | 1 | 1 | 1 | 1 | 1 | 1 | 1 | 1 | 1 | 1 | 1 |
| smi_0615 | 1 | 1 | 1 | 1 | 1 | 1 | 1 | 1 | 1 | 1 | -1 |
| smi_0616 | 1 | 1 | 1 | 1 | 1 | 1 | 1 | 1 | 1 | 1 | 1 |
| smi_0617 | 1 | 1 | 1 | 1 | 1 | 1 | 1 | 1 | 1 | 1 | 1 |
| smi_0619 | -1 | -1 | -1 | -1 | -1 | -1 | -1 | -1 | -1 | -1 | -1 |
| smi_0620 | 1 | 1 | 1 | 1 | 1 | 0 | 1 | 1 | 1 | 1 | 1 |
| smi_0621 | 1 | 1 | 1 | -1 | -1 | 1 | 1 | -1 | 1 | 1 | -1 |
| smi_0622 | 1 | 1 | 1 | 0 | 0 | 1 | 1 | 0 | 1 | 1 | 0 |
| smi_0623 | 1 | 1 | 1 | 1 | -1 | 1 | 1 | -1 | 1 | 1 | -1 |
| smi_0624 | 1 | 1 | 1 | -1 | -1 | 1 | 1 | -1 | 1 | 1 | -1 |
| smi_0625 | 1 | 1 | 1 | -1 | -1 | 1 | 1 | -1 | 1 | 1 | -1 |
| smi_0626 | 1 | 1 | 1 | -1 | 0 | 1 | 1 | 1 | 1 | 1 | 0 |
| smi_0627 | 1 | 1 | 1 | 1 | 1 | 1 | 1 | 1 | 1 | 1 | 1 |
| smi_0628 | -1 | -1 | -1 | -1 | -1 | 1 | -1 | -1 | -1 | -1 | -1 |
| smi_0629 | 1 | 1 | 1 | 1 | 1 | 1 | 1 | 1 | 1 | 1 | 1 |
| smi_0630 | 1 | 1 | 1 | 1 | -1 | 1 | -1 | -1 | -1 | -1 | 1 |
| smi_0631 | 1 | 0 | 1 | 1 | 1 | 1 | -1 | 1 | 1 | 1 | 1 |
| smi_0632 | 1 | 1 | 1 | 1 | 1 | 1 | -1 | 1 | -1 | -1 | 1 |
| smi_0633 | 1 | 1 | 1 | 1 | 1 | 1 | 1 | 1 | 1 | 1 | 1 |
| smi_0634 | 1 | 1 | 1 | 1 | 1 | 1 | 1 | 1 | 1 | 1 | 1 |
| smi_0635 | 1 | 1 | 1 | 1 | 1 | 1 | 1 | 1 | 1 | 1 | 1 |
| smi_0636 | 1 | 1 | 1 | 1 | 1 | 1 | 1 | 1 | 1 | 1 | 1 |
| smi_0637 | -1 | -1 | -1 | -1 | -1 | -1 | -1 | -1 | -1 | -1 | -1 |
| smi_0638 | -1 | -1 | -1 | -1 | -1 | -1 | -1 | -1 | -1 | -1 | -1 |
| smi_0639 | 1 | 1 | 1 | 1 | 1 | 1 | 1 | 1 | 1 | 1 | -1 |
| smi_0640 | 1 | 1 | 1 | 1 | 1 | 1 | 1 | 1 | 1 | 1 | 1 |
| smi_0641 | -1 | -1 | -1 | 1 | 1 | -1 | -1 | 1 | -1 | -1 | 1 |
| smi_0642 | 1 | 1 | 1 | 1 | 1 | 1 | 1 | 1 | 1 | 1 | 1 |
| smi_0643 | 1 | 1 | 1 | 1 | 1 | 1 | 1 | 1 | 1 | 1 | 1 |
| smi_0644 | 1 | 1 | 1 | 1 | 1 | -1 | 1 | 1 | 1 | 1 | 1 |
| smi_0645 | 1 | 1 | 1 | 1 | 1 | 1 | 1 | 1 | 1 | 1 | 1 |
| smi_0646 | 1 | 1 | 1 | 1 | 1 | 1 | 1 | 1 | 1 | 1 | 1 |
| smi_0647 | 1 | 1 | 1 | 1 | 1 | 1 | 1 | 1 | 1 | 1 | 1 |
| smi_0648 | 1 | 1 | 1 | 1 | 1 | 1 | 1 | 1 | 1 | 1 | 1 |
| smi_0649 | 1 | 1 | 1 | 1 | 1 | 1 | 1 | 1 | 1 | 1 | 1 |
| smi_0650 | 1 | 1 | 1 | 1 | 1 | 1 | 1 | 1 | 1 | 1 | 1 |
| smi_0651 | -1 | 1 | 1 | 1 | 1 | 1 | 1 | -1 | 1 | 1 | -1 |
| smi_0652 | 1 | 1 | 1 | 1 | 1 | 1 | 1 | -1 | 1 | 1 | -1 |
| smi_0653 | 1 | 1 | 1 | 1 | 1 | 1 | 1 | 1 | 1 | 1 | 1 |
| smi_0654 | 1 | 1 | -1 | 1 | 1 | 1 | -1 | 1 | -1 | -1 | 1 |
| smi_0655 | 1 | 1 | -1 | 1 | 1 | 1 | -1 | 1 | -1 | -1 | 1 |
| smi_0656 | 1 | 1 | 1 | 1 | 1 | 1 | 1 | 1 | 1 | 1 | 1 |
| smi_0657 | 1 | 1 | 1 | 1 | 1 | 1 | 1 | -1 | 1 | 1 | -1 |
| smi_0658 | 1 | 1 | 1 | 1 | 1 | 1 | 1 | -1 | 1 | 1 | -1 |
| smi_0659 | 1 | 1 | 1 | 1 | 1 | 1 | 1 | -1 | 1 | 1 | -1 |
| smi_0660 | 1 | 1 | 1 | 1 | 1 | 1 | 1 | -1 | 1 | 1 | -1 |
| smi_0661 | 1 | 1 | 1 | 1 | 1 | 1 | 1 | 1 | 1 | 1 | 1 |
| smi_0663 | 1 | 1 | 1 | 1 | 1 | 0 | 1 | 1 | 1 | 1 | 1 |
| smi_0664 | 1 | 1 | 1 | 1 | 1 | 0 | 1 | 1 | 1 | 1 | 1 |
| smi_0665 | 1 | 1 | 1 | 1 | 1 | 1 | 1 | 1 | 1 | 1 | 1 |
| smi_0666 | 1 | 1 | 1 | 1 | 1 | 1 | 1 | 1 | 1 | 1 | 1 |
| smi_0667 | 1 | 1 | 1 | 1 | 1 | 1 | 1 | 1 | 1 | 1 | 1 |
| smi_0668 | 1 | 1 | 1 | 1 | 1 | 1 | 1 | 1 | 1 | 1 | 1 |
| smi_0669 | 1 | 1 | 1 | 1 | 1 | 1 | 1 | 1 | 1 | 1 | 1 |
| smi_0670 | 1 | 1 | 1 | 1 | 1 | 1 | 1 | 1 | 1 | 1 | 1 |
| smi_0671 | 1 | 1 | 1 | 1 | -1 | 1 | -1 | -1 | -1 | -1 | -1 |
| smi_0672 | 1 | 1 | 1 | 1 | 1 | 1 | 1 | 1 | 1 | 1 | 1 |
| smi_0673 | 1 | 1 | 1 | 1 | 1 | 1 | 1 | 1 | 1 | 1 | 1 |
| smi_0674 | 1 | 1 | 1 | 1 | 1 | 1 | 1 | 1 | 1 | 1 | 1 |
| smi_0675 | 1 | 1 | 1 | 1 | 1 | 1 | 1 | 1 | 1 | 1 | 1 |
| smi_0676 | 1 | 1 | 1 | 1 | 1 | 1 | 1 | 1 | 1 | 1 | 1 |
| smi_0677 | 1 | 1 | 1 | 1 | 1 | 1 | 1 | 1 | 1 | 1 | 1 |
| smi_0678 | 1 | 1 | 1 | 1 | 1 | 1 | 1 | 1 | 1 | 1 | 1 |
| smi_0679 | 1 | 1 | 1 | 1 | 1 | 1 | 1 | 1 | 1 | 1 | 1 |
| smi_0680 | 1 | 1 | 1 | 1 | 1 | 1 | 1 | 1 | 1 | 1 | 1 |
| smi_0681 | 1 | 1 | 1 | 1 | 0 | 1 | 1 | 0 | 1 | 1 | 1 |
| smi_0682 | 1 | 1 | 1 | 1 | 1 | 1 | 1 | 1 | 1 | 1 | 1 |
| smi_0683 | 1 | 1 | -1 | -1 | -1 | -1 | -1 | -1 | -1 | -1 | -1 |
| smi_0684 | 1 | 1 | -1 | -1 | -1 | -1 | -1 | -1 | -1 | -1 | -1 |
| smi_0685 | 1 | 1 | 1 | 1 | 1 | 1 | 1 | 1 | 1 | 1 | 1 |
| smi_0686 | -1 | -1 | -1 | -1 | -1 | -1 | -1 | -1 | -1 | -1 | -1 |
| smi_0687 | 1 | 1 | 1 | 1 | 1 | 1 | 1 | 0 | 1 | 1 | 1 |
| smi_0688 | 1 | 1 | 1 | 1 | 1 | 1 | 1 | 1 | 1 | 1 | 1 |
| smi_0689 | 1 | 1 | 1 | 1 | 1 | 1 | 1 | 1 | 1 | 1 | 1 |
| smi_0690 | 1 | 1 | 1 | 1 | 1 | 1 | 1 | 1 | 1 | 1 | 1 |
| smi_0691 | 1 | 1 | 1 | 1 | 1 | 1 | 1 | 1 | 1 | 1 | 1 |
| smi_0692 | 1 | 1 | 1 | 1 | 1 | 1 | 1 | 1 | 1 | 1 | 1 |
| smi_0693 | 1 | 1 | 1 | 1 | 1 | 1 | 1 | 1 | 1 | 1 | 1 |
| smi_0694 | 1 | 1 | 1 | 1 | 0 | 1 | 1 | 1 | 1 | 1 | 1 |
| smi_0695 | 1 | 1 | 1 | 1 | 1 | 1 | 1 | 1 | 1 | 1 | 1 |
| smi_0696 | -1 | 1 | -1 | -1 | -1 | -1 | -1 | -1 | -1 | -1 | -1 |
| smi_0697 | 1 | 1 | -1 | -1 | -1 | -1 | -1 | -1 | -1 | -1 | -1 |
| smi_0698 | 1 | 1 | -1 | -1 | -1 | -1 | -1 | -1 | -1 | -1 | -1 |
| smi_0699 | -1 | -1 | -1 | -1 | -1 | 0 | 0 | -1 | -1 | -1 | -1 |
| smi_0700 | -1 | -1 | -1 | -1 | -1 | -1 | -1 | -1 | -1 | -1 | -1 |
| smi_0701 | 1 | 1 | 1 | 1 | 1 | 1 | 1 | 1 | 1 | 1 | 1 |
| smi_0702 | 1 | 1 | 1 | 1 | 0 | 1 | 1 | 1 | 0 | 1 | 1 |
| smi_0703 | 1 | 1 | 1 | 1 | 1 | 1 | 1 | 1 | 1 | 1 | 1 |
| smi_0704 | 1 | 1 | 1 | 1 | 1 | 1 | 1 | 1 | 1 | 1 | 1 |
| smi_0705 | -1 | -1 | -1 | 1 | -1 | -1 | 1 | -1 | 1 | 1 | -1 |
| smi_0706 | 1 | 1 | -1 | 1 | 1 | 1 | 1 | 1 | 1 | 1 | -1 |
| smi_0707 | 1 | 1 | 1 | 1 | 1 | 1 | 1 | 1 | 1 | 1 | 1 |
| smi_0708 | 1 | 1 | 1 | 1 | 1 | 1 | 1 | 1 | 1 | 1 | 1 |
| smi_0709 | 1 | 1 | 1 | 1 | 1 | 1 | 1 | 1 | 1 | 1 | 1 |
| smi_0710 | -1 | 1 | 1 | 1 | 1 | 1 | 1 | 1 | 1 | 1 | 1 |
| smi_0711 | 1 | 1 | 1 | 1 | 1 | 1 | 1 | 1 | 1 | 1 | 1 |
| smi_0712 | 1 | -1 | -1 | 1 | 0 | -1 | -1 | 0 | -1 | -1 | 0 |
| smi_0713 | 1 | 1 | 1 | 1 | 1 | 1 | 1 | 1 | 1 | 1 | 1 |
| smi_0714 | 1 | 1 | 1 | 1 | 1 | 1 | 1 | 1 | 1 | 1 | 1 |
| smi_0715 | 1 | 1 | 1 | 1 | 1 | 1 | 1 | 1 | 1 | 1 | 1 |
| smi_0716 | 1 | 1 | 1 | 1 | 1 | 1 | 1 | 1 | 1 | 1 | 1 |
| smi_0717 | 1 | 1 | 1 | 1 | 1 | 1 | 1 | 1 | 1 | 1 | 1 |
| smi_0718 | 1 | 1 | 1 | 1 | 1 | 1 | 1 | 1 | 1 | 1 | 1 |
| smi_0719 | 1 | 1 | 1 | 1 | 1 | 1 | 1 | 1 | 1 | 1 | 1 |
| smi_0720 | 1 | 1 | 1 | 1 | 1 | 1 | 1 | 1 | 1 | 1 | 1 |
| smi_0721 | 1 | 1 | 1 | 1 | 1 | 1 | 1 | 1 | 1 | 1 | 1 |
| smi_0722 | 1 | 1 | 1 | 1 | 1 | 1 | 1 | 1 | 1 | 1 | 1 |
| smi_0723 | 1 | 1 | 1 | 1 | 1 | 1 | 1 | 1 | 1 | 1 | 1 |
| smi_0724 | 1 | -1 | -1 | -1 | -1 | -1 | -1 | -1 | -1 | -1 | -1 |
| smi_0725 | 1 | -1 | -1 | -1 | -1 | -1 | -1 | -1 | -1 | -1 | -1 |
| smi_0726 | 1 | -1 | -1 | -1 | -1 | -1 | -1 | -1 | -1 | -1 | -1 |
| smi_0727 | 1 | -1 | -1 | -1 | -1 | -1 | -1 | -1 | -1 | -1 | -1 |
| smi_0728 | 1 | 1 | 1 | 1 | 1 | 1 | 1 | 1 | 1 | 1 | 1 |
| smi_0729 | 1 | 1 | 1 | 1 | 1 | 1 | 1 | 1 | 1 | 1 | 1 |
| smi_0730 | 1 | 1 | 1 | 1 | 1 | 1 | 1 | 1 | 1 | 1 | 1 |
| smi_0731 | 1 | 1 | 1 | 1 | 1 | 1 | 1 | 1 | 1 | 1 | 1 |
| smi_0732 | 1 | 1 | 1 | 1 | 1 | 1 | 1 | 1 | 1 | 1 | -1 |
| smi_0733 | 1 | 1 | 1 | 1 | 1 | 1 | 1 | 1 | 1 | 1 | -1 |
| smi_0734 | 1 | 1 | 1 | 1 | 1 | 1 | 1 | 1 | 1 | 1 | 1 |
| smi_0735 | 1 | 1 | 1 | 1 | 1 | 1 | 1 | 1 | 1 | 1 | 0 |
| smi_0736 | 1 | 1 | 1 | 1 | 1 | 1 | 1 | 1 | 1 | 1 | 0 |
| smi_0737 | 1 | 1 | 1 | 1 | 1 | 1 | 1 | 1 | 1 | 1 | -1 |
| smi_0738 | 1 | 1 | 1 | 1 | 1 | 1 | 1 | 1 | 1 | 1 | 1 |
| smi_0739 | 1 | 1 | 1 | 1 | 1 | 1 | 1 | 1 | 1 | 1 | 1 |
| smi_0740 | 1 | 1 | 1 | 1 | 1 | 1 | 1 | 1 | 1 | 1 | 1 |
| smi_0741 | 1 | 1 | 1 | 1 | 1 | 1 | 1 | 1 | 1 | 1 | 1 |
| smi_0742 | 1 | 1 | 1 | 1 | 1 | 1 | 1 | 1 | 1 | 1 | 1 |
| smi_0743 | 1 | 1 | 1 | 1 | 1 | 1 | 1 | 1 | 1 | 1 | 1 |
| smi_0744 | 1 | 1 | 1 | 1 | 1 | 1 | 1 | 1 | 1 | 1 | 1 |
| smi_0745 | 1 | 1 | 1 | 1 | 1 | 1 | 1 | 1 | 1 | 1 | 1 |
| smi_0746 | 1 | 0 | 1 | 1 | 1 | 1 | 1 | 1 | 1 | 1 | 1 |
| smi_0747 | -1 | -1 | -1 | -1 | -1 | -1 | -1 | -1 | -1 | -1 | -1 |
| smi_0748 | 1 | 1 | 1 | 1 | 1 | 1 | 1 | 1 | 1 | 1 | 1 |
| smi_0749 | 1 | 1 | 1 | 1 | 1 | 1 | 1 | 1 | 1 | 1 | 1 |
| smi_0750 | 1 | 1 | 1 | 1 | 1 | 0 | 1 | 1 | 1 | 1 | 1 |
| smi_0751 | 1 | 1 | 1 | 1 | 1 | 1 | 1 | 1 | 1 | 1 | 1 |
| smi_0752 | 1 | 1 | 1 | 1 | 1 | 1 | 1 | 1 | 1 | 1 | 1 |
| smi_0753 | 1 | 1 | -1 | 1 | 1 | 1 | 1 | 1 | -1 | -1 | -1 |
| smi_0754 | 1 | 0 | 1 | 1 | 1 | 1 | 1 | 1 | 1 | 1 | 1 |
| smi_0755 | 1 | 1 | 1 | 1 | 1 | 1 | 1 | 1 | 1 | 1 | 1 |
| smi_0756 | 1 | 1 | 1 | 1 | 1 | 1 | 1 | 1 | 1 | 1 | 1 |
| smi_0757 | 1 | 1 | 1 | 1 | 1 | 1 | 1 | 1 | 1 | 1 | 1 |
| smi_0758 | 1 | 1 | 1 | 1 | 1 | 1 | 1 | 1 | 1 | 1 | 1 |
| smi_0759 | 1 | 1 | 1 | 1 | 1 | 1 | 1 | 1 | 1 | 1 | 1 |
| smi_0760 | 1 | 1 | 1 | 1 | 1 | 1 | 1 | 1 | 1 | 1 | 1 |
| smi_0761 | 1 | 1 | -1 | -1 | -1 | 1 | 1 | -1 | -1 | -1 | -1 |
| smi_0762 | 1 | 1 | -1 | -1 | -1 | 1 | 1 | -1 | -1 | -1 | -1 |
| smi_0763 | 1 | 1 | 1 | 1 | 1 | 1 | 1 | 1 | 1 | 1 | 1 |
| smi_0764 | 1 | 1 | 1 | 1 | 1 | 1 | 1 | 1 | 1 | 1 | 1 |
| smi_0765 | 1 | 1 | 1 | 1 | 1 | 1 | 1 | 1 | 1 | 1 | 1 |
| smi_0766 | 1 | -1 | -1 | 1 | -1 | -1 | -1 | -1 | -1 | -1 | -1 |
| smi_0767 | 1 | 1 | 1 | 1 | 1 | 1 | 1 | 1 | 1 | 1 | 1 |
| smi_0768 | 1 | 1 | 1 | 1 | 1 | 1 | 1 | -1 | -1 | -1 | 1 |
| smi_0769 | 1 | 1 | 1 | 1 | 1 | 1 | 1 | -1 | -1 | -1 | 1 |
| smi_0770 | 1 | 1 | 1 | 1 | 1 | 1 | 1 | -1 | -1 | -1 | 1 |
| smi_0771 | 1 | 1 | 1 | 1 | 1 | 1 | 1 | -1 | -1 | -1 | 1 |
| smi_0772 | 1 | 1 | 1 | 1 | 1 | 1 | 1 | 1 | 1 | 1 | 1 |
| smi_0773 | 1 | 1 | 1 | 1 | 1 | 1 | 1 | 1 | 1 | 1 | 1 |
| smi_0774 | 1 | 1 | 1 | 1 | 1 | 1 | 1 | 1 | 1 | 1 | 1 |
| smi_0775 | 1 | 1 | 1 | 1 | 1 | 1 | 1 | 1 | 1 | 1 | 1 |
| smi_0776 | 1 | 1 | 1 | 1 | 1 | 1 | 1 | 1 | 1 | 1 | 0 |
| smi_0777 | 1 | 1 | 1 | 1 | 1 | 1 | 1 | 1 | 1 | 1 | 1 |
| smi_0778 | 1 | 1 | 1 | 1 | 1 | 1 | 1 | 1 | 1 | 1 | -1 |
| smi_0783 | 1 | 1 | 1 | 1 | 1 | 1 | 1 | 1 | 1 | 1 | 1 |
| smi_0784 | 1 | 1 | 1 | 1 | 1 | 1 | 1 | 1 | 1 | 1 | 1 |
| smi_0785 | -1 | -1 | 1 | -1 | -1 | -1 | -1 | -1 | 1 | -1 | 1 |
| smi_0786 | -1 | -1 | 1 | -1 | -1 | -1 | -1 | -1 | 1 | -1 | -1 |
| smi_0787 | -1 | -1 | 1 | -1 | -1 | -1 | -1 | -1 | 1 | -1 | -1 |
| smi_0788 | -1 | 0 | 1 | -1 | -1 | -1 | -1 | -1 | 1 | -1 | 1 |
| smi_0789 | 0 | -1 | 1 | -1 | -1 | -1 | -1 | -1 | 1 | -1 | 1 |
| smi_0790 | -1 | -1 | 1 | -1 | -1 | -1 | -1 | -1 | 1 | -1 | 1 |
| smi_0791 | -1 | -1 | 1 | -1 | -1 | -1 | -1 | -1 | 1 | -1 | 1 |
| smi_0792 | -1 | -1 | -1 | -1 | -1 | -1 | -1 | -1 | -1 | -1 | -1 |
| smi_0793 | -1 | -1 | -1 | -1 | -1 | -1 | -1 | -1 | -1 | -1 | -1 |
| smi_0794 | 1 | -1 | -1 | 1 | -1 | -1 | -1 | -1 | -1 | -1 | -1 |
| smi_0795 | -1 | -1 | -1 | -1 | -1 | -1 | -1 | -1 | -1 | -1 | -1 |
| smi_0796 | -1 | -1 | -1 | -1 | -1 | -1 | -1 | -1 | -1 | -1 | -1 |
| smi_0797 | 1 | -1 | 0 | -1 | -1 | -1 | -1 | -1 | -1 | -1 | -1 |
| smi_0798 | -1 | -1 | -1 | -1 | -1 | -1 | -1 | -1 | -1 | -1 | -1 |
| smi_0799 | -1 | -1 | -1 | -1 | -1 | -1 | -1 | -1 | -1 | -1 | -1 |
| smi_0800 | -1 | -1 | -1 | -1 | -1 | -1 | -1 | -1 | -1 | -1 | -1 |
| smi_0801 | -1 | -1 | -1 | -1 | -1 | -1 | -1 | -1 | -1 | -1 | -1 |
| smi_0802 | -1 | 0 | -1 | -1 | -1 | -1 | -1 | -1 | -1 | -1 | -1 |
| smi_0803 | -1 | -1 | -1 | -1 | -1 | -1 | -1 | -1 | -1 | -1 | -1 |
| smi_0804 | -1 | -1 | -1 | -1 | -1 | -1 | -1 | -1 | -1 | -1 | -1 |
| smi_0805 | -1 | -1 | -1 | -1 | -1 | -1 | -1 | -1 | -1 | -1 | -1 |
| smi_0806 | -1 | -1 | -1 | -1 | -1 | -1 | -1 | -1 | -1 | -1 | -1 |
| smi_0807 | -1 | -1 | -1 | -1 | -1 | -1 | -1 | -1 | -1 | -1 | -1 |
| smi_0809 | -1 | -1 | 1 | -1 | -1 | -1 | 0 | 0 | -1 | -1 | 0 |
| smi_0810 | -1 | -1 | -1 | 0 | -1 | -1 | -1 | -1 | -1 | -1 | -1 |
| smi_0811 | -1 | -1 | 1 | -1 | -1 | -1 | 0 | 1 | -1 | -1 | 1 |
| smi_0813 | -1 | -1 | 1 | -1 | -1 | -1 | 0 | 0 | -1 | -1 | 0 |
| smi_0814 | 1 | 1 | 1 | 1 | 1 | 1 | 1 | 1 | 1 | 1 | 1 |
| smi_0815 | 1 | 1 | 1 | 1 | 1 | 1 | 1 | 1 | 1 | 1 | 1 |
| smi_0816 | 1 | 1 | 1 | 1 | 1 | 1 | 1 | 1 | 1 | 1 | 0 |
| smi_0817 | 1 | 1 | 1 | 1 | 1 | 1 | 1 | 1 | 1 | 1 | 1 |
| smi_0818 | 1 | 1 | 1 | 1 | 1 | 1 | 1 | 1 | 1 | 1 | 1 |
| smi_0819 | 1 | 1 | 1 | 1 | 1 | 1 | 1 | 1 | 1 | 1 | 1 |
| smi_0820 | 1 | 1 | 1 | 1 | 1 | 1 | 1 | 0 | 1 | 1 | 1 |
| smi_0821 | 1 | 1 | -1 | 1 | 1 | 1 | 1 | 1 | 1 | 1 | -1 |
| smi_0822 | 1 | -1 | 1 | 1 | 1 | 1 | 1 | 1 | 1 | 1 | 1 |
| smi_0823 | 1 | -1 | -1 | 1 | 1 | -1 | -1 | -1 | 1 | 1 | 0 |
| smi_0824 | 1 | 1 | 1 | 1 | -1 | -1 | 1 | -1 | -1 | -1 | -1 |
| smi_0825 | 1 | 1 | 1 | 1 | 1 | 1 | 1 | 1 | 1 | 1 | 1 |
| smi_0826 | 1 | 1 | 1 | 1 | 1 | 1 | 1 | 1 | 1 | 1 | 1 |
| smi_0827 | -1 | 1 | -1 | 1 | 1 | -1 | 1 | 1 | 1 | 1 | -1 |
| smi_0828 | -1 | 1 | 1 | 1 | -1 | 1 | -1 | 1 | 1 | 1 | 1 |
| smi_0829 | 1 | 1 | 0 | 1 | 1 | 1 | 1 | 1 | 1 | 1 | 1 |
| smi_0830 | 1 | 1 | 1 | 1 | 1 | 1 | 1 | 1 | 1 | 1 | 1 |
| smi_0831 | 1 | 1 | 1 | 1 | 1 | 1 | 1 | 1 | 1 | 1 | 1 |
| smi_0832 | 1 | 1 | 1 | 1 | 1 | 1 | 1 | 1 | 1 | 1 | 1 |
| smi_0833 | 1 | 1 | 1 | 1 | 1 | 1 | 1 | 1 | 1 | 1 | 1 |
| smi_0834 | 1 | 1 | 1 | 1 | 1 | 1 | 1 | 1 | 1 | 1 | 1 |
| smi_0835 | -1 | 1 | -1 | 1 | 1 | -1 | -1 | -1 | 1 | -1 | -1 |
| smi_0836 | -1 | 1 | -1 | 1 | 1 | -1 | -1 | -1 | 1 | -1 | -1 |
| smi_0837 | -1 | 1 | -1 | 1 | 1 | -1 | -1 | -1 | 1 | -1 | -1 |
| smi_0838 | 1 | 1 | 1 | 1 | 1 | 1 | 1 | 1 | 1 | 1 | 1 |
| smi_0839 | 1 | 1 | 0 | 1 | 1 | 1 | 1 | 1 | 1 | 1 | -1 |
| smi_0840 | 1 | 1 | 1 | 1 | 1 | 1 | 1 | 1 | 1 | 1 | 1 |
| smi_0841 | 1 | 1 | 1 | 1 | 1 | 1 | 1 | 1 | -1 | 0 | 1 |
| smi_0842 | 1 | 1 | 1 | 1 | 1 | 1 | 1 | 1 | 1 | 1 | 1 |
| smi_0843 | 1 | 1 | 1 | -1 | 1 | 1 | 1 | 1 | 1 | -1 | 1 |
| smi_0844 | 1 | 1 | 1 | 0 | 1 | 1 | 1 | 1 | 1 | 1 | 1 |
| smi_0845 | 1 | 1 | 1 | 1 | 1 | 1 | 1 | 1 | 1 | 1 | 1 |
| smi_0846 | 1 | 1 | 1 | 1 | 0 | 0 | 0 | 1 | -1 | 0 | 1 |
| smi_0847 | 1 | -1 | -1 | -1 | 1 | -1 | -1 | 1 | -1 | -1 | 1 |
| smi_0848 | 1 | -1 | -1 | 0 | -1 | -1 | -1 | 0 | -1 | -1 | -1 |
| smi_0849 | 1 | 1 | 1 | 1 | 1 | 1 | 1 | 1 | 1 | 1 | 1 |
| smi_0850 | 1 | 1 | 1 | 1 | 1 | 1 | 1 | 1 | 1 | 1 | 1 |
| smi_0851 | 1 | 1 | 1 | 1 | 1 | 1 | 1 | 1 | 1 | 1 | 1 |
| smi_0852 | 1 | 1 | 1 | 1 | 1 | 1 | 1 | 1 | 1 | 1 | 1 |
| smi_0853 | 1 | 1 | 1 | 1 | -1 | 1 | 1 | 0 | 1 | 1 | 1 |
| smi_0854 | 0 | 1 | 1 | 1 | 1 | 0 | 1 | 1 | 1 | 1 | 1 |
| smi_0855 | 1 | 1 | 1 | 0 | -1 | 1 | -1 | 1 | -1 | -1 | 0 |
| smi_0856 | 1 | 1 | 1 | 1 | 1 | 1 | 1 | 1 | 1 | 1 | 1 |
| smi_0857 | 1 | 1 | 1 | 1 | 1 | 1 | 1 | 1 | 1 | 1 | 1 |
| smi_0858 | -1 | -1 | -1 | -1 | -1 | -1 | -1 | -1 | -1 | -1 | -1 |
| smi_0859 | 1 | 1 | 1 | 1 | 1 | 1 | 1 | 1 | 1 | 1 | 1 |
| smi_0860 | 1 | 1 | 1 | 1 | 1 | 1 | 1 | 1 | 1 | 1 | 1 |
| smi_0861 | 1 | 1 | 1 | 1 | -1 | 1 | 1 | 1 | 1 | 1 | 0 |
| smi_0862 | 1 | 1 | -1 | -1 | -1 | 1 | 1 | -1 | 1 | 1 | -1 |
| smi_0863 | 1 | 1 | 1 | 1 | 1 | 1 | 1 | 1 | 1 | 1 | 1 |
| smi_0864 | 1 | 1 | 1 | 1 | 1 | 1 | 1 | 1 | 1 | 1 | 1 |
| smi_0865 | 1 | 1 | 1 | 1 | 1 | 1 | 1 | 1 | 1 | 1 | 1 |
| smi_0866 | 1 | 1 | 1 | 1 | 1 | 1 | 1 | 1 | 1 | 1 | 1 |
| smi_0867 | 1 | 1 | 1 | 1 | 1 | -1 | 1 | 1 | 1 | -1 | -1 |
| smi_0868 | 1 | 1 | 1 | 1 | 1 | 1 | 1 | 1 | 1 | 1 | 1 |
| smi_0869 | 1 | 0 | 1 | 1 | 1 | 1 | 1 | 1 | 1 | 1 | 1 |
| smi_0870 | 0 | 1 | 1 | -1 | 1 | -1 | -1 | -1 | -1 | -1 | -1 |
| smi_0871 | 1 | 1 | 1 | 1 | 1 | 1 | 1 | 1 | 1 | 1 | 1 |
| smi_0872 | 1 | 1 | 1 | 1 | 0 | 1 | 1 | -1 | 1 | 1 | -1 |
| smi_0873 | 1 | 1 | 1 | 1 | 1 | 1 | 1 | 1 | 1 | 1 | 1 |
| smi_0874 | 1 | 1 | 1 | 1 | 1 | 1 | 1 | 1 | 1 | 1 | 1 |
| smi_0875 | 1 | 1 | 1 | 1 | 1 | 1 | 1 | 1 | 0 | 1 | 1 |
| smi_0876 | 1 | 1 | 1 | 1 | 1 | 1 | 1 | 1 | 1 | 1 | 1 |
| smi_0877 | 1 | 1 | -1 | 1 | 1 | 1 | 1 | 1 | 1 | 1 | -1 |
| smi_0878 | 1 | 1 | -1 | -1 | -1 | -1 | -1 | -1 | -1 | -1 | -1 |
| smi_0879 | 1 | 1 | 1 | 1 | 1 | 1 | 1 | 1 | 1 | 1 | 1 |
| smi_0880 | 1 | 1 | 1 | -1 | -1 | -1 | -1 | -1 | -1 | -1 | -1 |
| smi_0881 | 1 | 1 | 1 | 1 | 1 | 1 | 1 | 1 | 1 | 1 | 1 |
| smi_0882 | 1 | 1 | 1 | 1 | 1 | 1 | 1 | 1 | 1 | 1 | 1 |
| smi_0883 | 1 | 1 | 1 | 1 | 1 | 1 | 1 | 1 | 1 | 1 | 1 |
| smi_0884 | 1 | 1 | 1 | 1 | 1 | 1 | -1 | 1 | -1 | -1 | 1 |
| smi_0885 | -1 | -1 | -1 | -1 | -1 | 1 | -1 | -1 | -1 | -1 | -1 |
| smi_0886 | -1 | -1 | -1 | -1 | -1 | -1 | -1 | -1 | -1 | -1 | -1 |
| smi_0887 | 0 | -1 | -1 | -1 | -1 | 0 | -1 | -1 | -1 | -1 | -1 |
| smi_0888 | 1 | 0 | 1 | 1 | -1 | -1 | 1 | 1 | -1 | 1 | 1 |
| smi_0889 | 1 | 1 | 1 | 1 | 1 | 1 | 1 | 1 | 1 | 1 | 1 |
| smi_0890 | 1 | 1 | 1 | 1 | 1 | 1 | 1 | 1 | 1 | 1 | 1 |
| smi_0891 | 1 | 1 | 1 | 1 | 1 | 1 | 1 | 1 | 1 | 1 | 0 |
| smi_0892 | 1 | 1 | 1 | 1 | 0 | 1 | 1 | 0 | 1 | 1 | 0 |
| smi_0893 | 1 | 1 | 1 | 1 | 1 | 1 | 1 | 1 | 1 | 1 | 1 |
| smi_0894 | 1 | 1 | 1 | 1 | 1 | 1 | 1 | 1 | 1 | 1 | 1 |
| smi_0895 | 1 | 1 | 1 | 1 | 1 | 1 | 1 | 1 | 1 | 1 | 1 |
| smi_0896 | 1 | 1 | 1 | 1 | 1 | 1 | 1 | 1 | 1 | 1 | 1 |
| smi_0897 | 1 | 1 | 1 | 1 | 1 | 1 | 1 | 1 | 1 | 1 | 1 |
| smi_0898 | -1 | -1 | -1 | -1 | -1 | -1 | -1 | -1 | -1 | -1 | -1 |
| smi_0899 | 0 | 0 | 0 | -1 | 1 | 0 | 0 | 0 | -1 | 0 | 0 |
| smi_0900 | -1 | -1 | -1 | -1 | -1 | -1 | -1 | -1 | -1 | -1 | -1 |
| smi_0901 | 1 | -1 | -1 | -1 | -1 | -1 | -1 | -1 | -1 | -1 | -1 |
| smi_0902 | 1 | -1 | -1 | -1 | -1 | -1 | -1 | -1 | -1 | -1 | -1 |
| smi_0903 | 1 | -1 | -1 | -1 | -1 | -1 | -1 | -1 | -1 | -1 | -1 |
| smi_0904 | 1 | -1 | -1 | 1 | 1 | 1 | -1 | 0 | 1 | 1 | -1 |
| smi_0905 | 1 | 1 | 1 | 1 | 1 | 1 | 1 | 1 | 1 | 1 | 1 |
| smi_0906 | 1 | 1 | 1 | 1 | 1 | 1 | 1 | 1 | 1 | 1 | 1 |
| smi_0907 | 0 | 1 | 1 | 1 | 1 | 1 | 1 | 1 | 1 | 1 | 1 |
| smi_0908 | 1 | 1 | 1 | 1 | 1 | 1 | 1 | 1 | 1 | 1 | 1 |
| smi_0909 | 1 | 1 | 1 | 1 | 1 | 1 | 1 | 1 | 1 | 1 | 1 |
| smi_0911 | 1 | 1 | 1 | 1 | 1 | 1 | 1 | 1 | 1 | 1 | 1 |
| smi_0912 | 1 | 1 | 1 | 1 | 1 | 1 | 1 | 1 | 1 | 1 | 1 |
| smi_0913 | 1 | 1 | 1 | 1 | 1 | 1 | 1 | 1 | 1 | 1 | -1 |
| smi_0914 | 1 | 1 | 1 | 1 | 1 | 1 | 1 | 1 | 1 | 1 | 1 |
| smi_0915 | 1 | 1 | 1 | 1 | 1 | 1 | 1 | 1 | 1 | 1 | 1 |
| smi_0916 | 1 | 1 | 1 | 1 | 1 | 1 | 1 | 1 | 1 | 1 | 1 |
| smi_0917 | 1 | 1 | 1 | 1 | 1 | 1 | 1 | 1 | 1 | 1 | 1 |
| smi_0918 | 1 | 1 | 1 | 1 | 1 | 1 | 1 | 1 | 1 | 1 | 1 |
| smi_0919 | 1 | 1 | 1 | 1 | 1 | 1 | 1 | 1 | 1 | 1 | 1 |
| smi_0920 | 1 | 1 | 1 | 1 | 1 | 0 | 1 | 1 | 1 | 1 | 1 |
| smi_0921 | 1 | 1 | 1 | 1 | 1 | 1 | 1 | 0 | 1 | 1 | -1 |
| smi_0922 | 1 | 1 | 1 | 1 | 1 | 1 | 1 | 1 | 1 | 1 | 1 |
| smi_0923 | 1 | 1 | 1 | 1 | 1 | 1 | 1 | 1 | 1 | 1 | 1 |
| smi_0924 | 1 | 1 | 1 | 1 | 1 | 1 | 1 | 1 | 1 | 1 | 1 |
| smi_0930 | 1 | 1 | 1 | 1 | 1 | 1 | 1 | 1 | 1 | 1 | 1 |
| smi_0931 | 1 | 1 | 1 | 1 | 1 | 1 | 1 | 1 | 1 | 1 | 1 |
| smi_0932 | 1 | 1 | 1 | 1 | 1 | 1 | 1 | 1 | 1 | 1 | 1 |
| smi_0933 | -1 | -1 | -1 | -1 | 1 | -1 | -1 | -1 | -1 | -1 | -1 |
| smi_0934 | 1 | 1 | 1 | -1 | 1 | 1 | -1 | 1 | -1 | -1 | 1 |
| smi_0935 | 1 | 1 | 1 | 1 | 1 | 1 | 1 | 1 | 1 | 1 | 1 |
| smi_0936 | 1 | 1 | 1 | 1 | 1 | 1 | 1 | 1 | 1 | 1 | 1 |
| smi_0937 | 1 | 1 | 0 | 1 | 1 | 1 | 1 | 1 | 1 | 1 | 1 |
| smi_0938 | 1 | 1 | 1 | 1 | 1 | 1 | 1 | 1 | 1 | 1 | 1 |
| smi_0939 | 1 | 1 | 1 | 1 | 1 | 1 | 1 | 1 | 1 | 1 | 1 |
| smi_0940 | 1 | 1 | 1 | 1 | 1 | 1 | 1 | 1 | 1 | 1 | 1 |
| smi_0941 | 1 | 0 | 0 | 1 | 0 | 0 | 0 | 0 | 1 | 0 | -1 |
| smi_0942 | -1 | -1 | -1 | -1 | -1 | -1 | -1 | -1 | -1 | -1 | -1 |
| smi_0943 | -1 | -1 | -1 | -1 | -1 | -1 | -1 | -1 | -1 | -1 | -1 |
| smi_0944 | -1 | -1 | -1 | -1 | -1 | -1 | -1 | -1 | -1 | -1 | -1 |
| smi_0945 | -1 | -1 | -1 | -1 | -1 | -1 | -1 | -1 | -1 | -1 | -1 |
| smi_0946 | -1 | -1 | -1 | -1 | -1 | -1 | -1 | -1 | -1 | -1 | -1 |
| smi_0947 | -1 | -1 | -1 | -1 | -1 | -1 | -1 | -1 | -1 | -1 | -1 |
| smi_0948 | 1 | 1 | 1 | 1 | 1 | 1 | 1 | 1 | 1 | 1 | 1 |
| smi_0949 | 1 | 1 | 1 | 1 | 1 | 1 | 1 | 1 | 1 | 1 | 1 |
| smi_0950 | 1 | 1 | 1 | 1 | 1 | 1 | 1 | 1 | 1 | 1 | 1 |
| smi_0951 | 1 | 1 | 1 | 1 | 1 | 1 | 1 | 1 | 1 | 1 | 1 |
| smi_0952 | 1 | 1 | 1 | 1 | 1 | 1 | 1 | 1 | 1 | 1 | 1 |
| smi_0953 | 1 | 1 | 1 | 1 | 1 | 1 | 1 | 1 | 1 | 1 | 1 |
| smi_0954 | 1 | 1 | 1 | 1 | 1 | -1 | 1 | 1 | 1 | 1 | 1 |
| smi_0955 | 1 | 1 | 1 | 1 | 1 | 0 | 1 | 1 | 1 | 1 | 1 |
| smi_0956 | 1 | 1 | 1 | 1 | 1 | 1 | 1 | 1 | 1 | 1 | 1 |
| smi_0957 | -1 | 1 | 1 | 1 | -1 | 1 | -1 | -1 | 1 | 1 | 0 |
| smi_0958 | -1 | 1 | 1 | 1 | -1 | 1 | -1 | -1 | 1 | 1 | 1 |
| smi_0959 | 1 | 1 | 1 | 1 | 1 | 1 | 1 | 1 | 1 | 1 | 1 |
| smi_0960 | 1 | 1 | 1 | 1 | 1 | 1 | 1 | 1 | 1 | 1 | 1 |
| smi_0961 | 1 | 1 | 1 | 1 | 1 | 1 | 1 | 1 | 1 | 1 | 1 |
| smi_0962 | 1 | 1 | 1 | 1 | 1 | 1 | 1 | 1 | 1 | 1 | 1 |
| smi_0964 | 1 | -1 | 1 | 1 | 1 | 1 | 1 | 1 | 0 | 1 | 1 |
| smi_0965 | 1 | 1 | 1 | 1 | 1 | 1 | 1 | 1 | 1 | 1 | 1 |
| smi_0966 | 1 | 1 | 1 | -1 | 1 | 1 | -1 | 1 | -1 | -1 | 1 |
| smi_0967 | 1 | 1 | 1 | 1 | 1 | 1 | 1 | 1 | 1 | 1 | 1 |
| smi_0968 | 1 | 1 | 1 | 1 | 1 | 1 | 1 | 1 | 1 | 1 | 1 |
| smi_0969 | 1 | 1 | 1 | 1 | 1 | 1 | 1 | 1 | 1 | 1 | 1 |
| smi_0970 | 1 | 1 | 1 | 1 | 1 | 1 | 1 | 1 | 1 | 1 | 1 |
| smi_0971 | 1 | 1 | 1 | 1 | 1 | -1 | 1 | 1 | 1 | 1 | 1 |
| smi_0972 | 1 | 1 | 1 | 1 | 0 | 1 | 0 | 0 | 1 | 0 | 1 |
| smi_0973 | 1 | 1 | 1 | 1 | 1 | 1 | 1 | 1 | 1 | 1 | 1 |
| smi_0974 | 1 | 1 | 1 | 1 | 1 | 1 | 1 | 1 | 1 | 1 | 1 |
| smi_0975 | 1 | 1 | 1 | 1 | 1 | 1 | 1 | 1 | 1 | 1 | 1 |
| smi_0976 | 1 | 1 | 1 | 1 | 1 | 1 | 1 | 1 | 1 | 1 | 1 |
| smi_0977 | 1 | 1 | 1 | 1 | 1 | 1 | 1 | 1 | 1 | 1 | 1 |
| smi_0978 | 1 | -1 | 1 | 1 | 1 | 1 | 1 | 1 | 1 | 1 | 1 |
| smi_0979 | -1 | -1 | -1 | 1 | -1 | -1 | -1 | -1 | -1 | -1 | -1 |
| smi_0980 | 1 | 1 | 1 | 1 | 1 | 1 | 1 | 1 | 1 | 1 | 1 |
| smi_0981 | 1 | 1 | 1 | 1 | 1 | 1 | 1 | 1 | 1 | 1 | 1 |
| smi_0982 | 1 | 1 | 1 | 1 | 1 | 1 | 1 | 1 | 1 | 1 | 1 |
| smi_0983 | 1 | 1 | 1 | 1 | 1 | 1 | 1 | 1 | 1 | 0 | 0 |
| smi_0984 | 1 | 1 | 1 | 1 | -1 | 1 | 1 | -1 | 1 | 1 | -1 |
| smi_0985 | 1 | 1 | 1 | 1 | 1 | 1 | 1 | 1 | 1 | 1 | 1 |
| smi_0986 | -1 | -1 | -1 | -1 | -1 | -1 | -1 | -1 | -1 | -1 | -1 |
| smi_0987 | -1 | 0 | -1 | -1 | -1 | -1 | -1 | -1 | -1 | -1 | -1 |
| smi_0988 | 1 | 1 | 1 | 1 | 1 | 1 | 1 | 1 | 1 | 1 | 0 |
| smi_0989 | 1 | 1 | 1 | 1 | 1 | 1 | 1 | 1 | 1 | 1 | 1 |
| smi_0990 | 1 | 1 | 1 | 1 | 1 | 1 | 1 | 1 | 1 | 1 | 1 |
| smi_0991 | 1 | 1 | 1 | 1 | 1 | 1 | 1 | 1 | 1 | 1 | 1 |
| smi_0992 | 1 | 1 | 1 | 1 | 1 | 1 | 1 | 1 | 1 | 1 | 1 |
| smi_0993 | 1 | 1 | 1 | 1 | 1 | 1 | 1 | 1 | 1 | 1 | 1 |
| smi_0994 | 1 | 1 | 1 | 1 | 1 | 1 | 1 | 1 | 1 | 1 | 1 |
| smi_0995 | 1 | 1 | 1 | 1 | 1 | 1 | 1 | 1 | 1 | 1 | 1 |
| smi_0996 | 1 | 1 | 1 | 1 | 0 | 1 | 1 | 1 | 1 | 1 | 1 |
| smi_0997 | 1 | 1 | 1 | 1 | 1 | 1 | 1 | 1 | 1 | 1 | 1 |
| smi_0998 | 1 | 1 | 1 | 1 | 1 | 1 | 1 | 1 | 1 | 1 | 1 |
| smi_0999 | 1 | 1 | 1 | 1 | 1 | 1 | 1 | 1 | 1 | 1 | 1 |
| smi_1000 | 1 | 1 | 1 | 1 | 1 | 1 | 1 | 1 | 1 | 1 | 1 |
| smi_1001 | 1 | 1 | 1 | 1 | 1 | 1 | 1 | 1 | 1 | 1 | 1 |
| smi_1002 | 1 | 0 | 0 | -1 | -1 | 1 | -1 | -1 | -1 | -1 | -1 |
| smi_1003 | 1 | 1 | 1 | 1 | 1 | 1 | 1 | 1 | -1 | 1 | 0 |
| smi_1004 | 1 | 1 | 1 | 1 | 1 | 1 | 1 | 1 | 1 | 1 | 1 |
| smi_1005 | 1 | 1 | 1 | 1 | 1 | 1 | 1 | 1 | 1 | 1 | 1 |
| smi_1006 | 1 | 1 | 1 | 1 | 1 | 1 | 1 | 1 | 1 | 1 | 1 |
| smi_1007 | 1 | 1 | 1 | 1 | 1 | 1 | 1 | 1 | 1 | 1 | 1 |
| smi_1008 | 1 | 1 | 1 | 1 | 1 | 1 | -1 | 1 | -1 | -1 | 1 |
| smi_1009 | 1 | 1 | 1 | 1 | 1 | 1 | 1 | 1 | 1 | 1 | 1 |
| smi_1010 | 1 | 1 | 1 | 1 | 1 | 1 | 1 | 1 | 1 | 1 | 1 |
| smi_1011 | 1 | 1 | 1 | 1 | 1 | 1 | 1 | 1 | 1 | 1 | 1 |
| smi_1012 | 1 | 1 | 1 | 1 | 1 | 1 | 1 | 1 | 1 | 1 | 1 |
| smi_1013 | 1 | 1 | 1 | 1 | 1 | 1 | 1 | 1 | 1 | 1 | 1 |
| smi_1014 | 1 | 1 | 1 | 1 | 1 | 1 | 1 | 1 | 1 | 1 | 1 |
| smi_1015 | 1 | 1 | 1 | 1 | 1 | 1 | 1 | 1 | 1 | 1 | 1 |
| smi_1016 | 1 | 1 | 1 | 1 | 1 | 1 | 1 | 1 | 1 | 1 | 1 |
| smi_1017 | 1 | 1 | 1 | 1 | 1 | 1 | 1 | 1 | 1 | 1 | 1 |
| smi_1018 | 1 | 1 | 1 | 1 | 1 | 1 | 1 | 1 | 1 | 1 | 1 |
| smi_1019 | 1 | 1 | 1 | 1 | 1 | 1 | 1 | 1 | 1 | 1 | 1 |
| smi_1020 | 1 | 1 | 1 | 1 | 1 | 1 | 1 | 1 | 1 | 1 | 1 |
| smi_1021 | 1 | 1 | 1 | 1 | 1 | 1 | 1 | 1 | 1 | 1 | 1 |
| smi_1022 | 1 | 1 | 1 | 1 | 1 | 1 | 1 | 1 | 1 | 1 | 1 |
| smi_1023 | 1 | 1 | 1 | 1 | 1 | 0 | 1 | 1 | 1 | 1 | 1 |
| smi_1024 | 1 | 1 | 1 | 1 | 1 | 1 | 1 | 1 | 1 | 1 | 1 |
| smi_1025 | 1 | 1 | 1 | 1 | 1 | 1 | 1 | 1 | 1 | 1 | -1 |
| smi_1026 | 1 | 1 | 1 | 1 | 1 | 1 | 1 | 1 | 1 | 1 | 1 |
| smi_1027 | 1 | 1 | 1 | 1 | 1 | 1 | 1 | 1 | 1 | 1 | 1 |
| smi_1028 | 1 | 1 | 1 | 1 | 1 | 1 | 1 | 1 | 1 | 1 | 1 |
| smi_1029 | 1 | 1 | 1 | 1 | 1 | 1 | 1 | 1 | 1 | 1 | 1 |
| smi_1030 | 1 | 1 | 1 | -1 | -1 | -1 | -1 | -1 | -1 | -1 | -1 |
| smi_1031 | 1 | 1 | 1 | -1 | -1 | -1 | -1 | -1 | -1 | -1 | -1 |
| smi_1032 | 1 | 1 | 1 | -1 | -1 | -1 | -1 | -1 | -1 | -1 | -1 |
| smi_1033 | 1 | 1 | 1 | -1 | -1 | -1 | -1 | -1 | -1 | -1 | -1 |
| smi_1034 | -1 | 1 | 1 | -1 | -1 | -1 | -1 | -1 | -1 | -1 | -1 |
| smi_1035 | 1 | 1 | 1 | -1 | -1 | -1 | -1 | -1 | -1 | -1 | -1 |
| smi_1036 | -1 | 1 | 1 | 1 | 1 | 1 | -1 | 1 | 1 | 1 | 1 |
| smi_1037 | 1 | 1 | 1 | 1 | 1 | 1 | -1 | 1 | 1 | 0 | 1 |
| smi_1038 | 1 | 1 | 1 | 1 | 1 | 1 | -1 | 1 | 1 | 1 | 1 |
| smi_1039 | 1 | 1 | 1 | 1 | 1 | 1 | -1 | 1 | 1 | 1 | 1 |
| smi_1040 | 1 | 1 | 1 | 1 | 1 | 1 | -1 | 0 | 0 | 1 | -1 |
| smi_1041 | 1 | 1 | 1 | 1 | -1 | 1 | 1 | -1 | 1 | 1 | -1 |
| smi_1042 | 1 | 1 | 1 | 1 | 1 | 1 | 1 | 1 | 1 | 1 | 1 |
| smi_1043 | 1 | 1 | 1 | 1 | 1 | 1 | 1 | 1 | 1 | 1 | 1 |
| smi_1044 | 1 | 1 | 1 | 1 | 1 | 1 | 1 | 1 | 1 | 1 | 1 |
| smi_1045 | 1 | 1 | 1 | 1 | 1 | 1 | 1 | 1 | 1 | 1 | 1 |
| smi_1046 | 1 | 1 | 1 | 1 | 1 | 1 | 1 | 1 | 1 | 1 | 1 |
| smi_1047 | 1 | 1 | 1 | 1 | 1 | 1 | 1 | 1 | 1 | 1 | -1 |
| smi_1048 | 1 | 1 | 1 | 1 | 1 | -1 | 1 | 1 | 1 | 1 | -1 |
| smi_1049 | 1 | 1 | -1 | 1 | 0 | 1 | -1 | 1 | -1 | -1 | -1 |
| smi_1050 | 1 | 1 | -1 | 1 | 1 | 1 | -1 | 1 | -1 | -1 | -1 |
| smi_1051 | 1 | 1 | -1 | 1 | 1 | 1 | -1 | 1 | -1 | -1 | -1 |
| smi_1052 | 1 | 1 | -1 | 1 | 1 | 1 | -1 | 1 | -1 | -1 | -1 |
| smi_1053 | 1 | 1 | -1 | 1 | 1 | 1 | -1 | 1 | -1 | -1 | 1 |
| smi_1054 | 1 | 1 | 1 | 1 | 1 | 1 | 1 | 1 | 1 | 1 | 1 |
| smi_1055 | 1 | 1 | 1 | 1 | 1 | 1 | 1 | 1 | 1 | 1 | 1 |
| smi_1056 | 1 | 1 | 1 | 1 | 1 | 1 | 1 | 1 | 1 | 1 | 1 |
| smi_1057 | 1 | 1 | 1 | 1 | 1 | 1 | 1 | 1 | 1 | 1 | 1 |
| smi_1058 | 1 | 1 | 1 | 1 | 1 | 1 | 1 | 1 | 1 | 1 | 1 |
| smi_1059 | 1 | 1 | 1 | 1 | 1 | 1 | 1 | 1 | 1 | 1 | 1 |
| smi_1060 | -1 | -1 | 1 | -1 | 1 | -1 | -1 | -1 | -1 | -1 | -1 |
| smi_1061 | 1 | 1 | 1 | 1 | 1 | 1 | 1 | 1 | 1 | 1 | -1 |
| smi_1062 | 1 | 1 | 1 | 1 | 1 | 1 | 1 | 1 | 1 | 1 | 1 |
| smi_1063 | 1 | 1 | 1 | 1 | 1 | 1 | 1 | 1 | 1 | 1 | 1 |
| smi_1064 | 0 | 1 | -1 | -1 | 1 | -1 | -1 | -1 | -1 | -1 | -1 |
| smi_1065 | 1 | 1 | 1 | 1 | 1 | 1 | 1 | -1 | 1 | 1 | 1 |
| smi_1066 | 1 | 1 | 1 | -1 | 0 | 0 | 1 | 1 | 1 | 1 | 0 |
| smi_1067 | 1 | 0 | 1 | 1 | 1 | 1 | 1 | 1 | 1 | 1 | 1 |
| smi_1069 | 1 | 1 | 1 | 1 | 1 | 1 | 1 | 1 | 1 | 1 | 1 |
| smi_1070 | 1 | 1 | 1 | 1 | 1 | 1 | 1 | 1 | 1 | 1 | -1 |
| smi_1071 | 1 | 1 | 1 | 1 | 1 | 1 | 1 | 1 | 1 | 1 | -1 |
| smi_1072 | 1 | 1 | 1 | 1 | 1 | 1 | 1 | 1 | 1 | 1 | -1 |
| smi_1073 | 1 | 1 | 1 | 1 | 1 | 1 | 1 | 1 | 1 | 1 | -1 |
| smi_1074 | 1 | 1 | 1 | 1 | 1 | 1 | 1 | 1 | 1 | 1 | 1 |
| smi_1075 | 1 | 1 | 1 | 1 | 1 | 1 | 1 | 1 | 1 | 1 | 1 |
| smi_1076 | 1 | 1 | 1 | 1 | 1 | 1 | 1 | 1 | 1 | 1 | 1 |
| smi_1077 | -1 | -1 | -1 | -1 | 1 | -1 | 1 | -1 | -1 | -1 | -1 |
| smi_1078 | -1 | -1 | -1 | -1 | 1 | -1 | 1 | -1 | -1 | -1 | -1 |
| smi_1079 | 1 | 1 | 1 | 1 | 1 | 1 | 1 | 1 | 1 | 1 | 1 |
| smi_1080 | 1 | 1 | 1 | 1 | 1 | 1 | 1 | 1 | 1 | 1 | 1 |
| smi_1081 | 1 | 1 | 1 | 1 | 1 | 1 | 1 | 1 | 1 | 1 | 1 |
| smi_1082 | 1 | 1 | -1 | -1 | 1 | 1 | -1 | 1 | 1 | 1 | -1 |
| smi_1083 | 1 | 1 | 1 | 1 | 1 | 1 | 1 | 1 | 1 | 1 | 1 |
| smi_1084 | 1 | 1 | 1 | 1 | 0 | 0 | 1 | 1 | 1 | 1 | 1 |
| smi_1085 | 1 | 1 | 1 | 1 | 1 | 1 | 1 | 1 | 1 | 1 | 1 |
| smi_1086 | -1 | 1 | -1 | -1 | -1 | -1 | -1 | 1 | -1 | 1 | -1 |
| smi_1087 | -1 | -1 | -1 | -1 | -1 | -1 | -1 | -1 | -1 | -1 | -1 |
| smi_1088 | 1 | 1 | 1 | 1 | 1 | 1 | 1 | -1 | -1 | 1 | 1 |
| smi_1089 | 1 | 1 | 1 | 1 | 1 | 1 | 1 | 1 | 1 | 1 | 1 |
| smi_1090 | -1 | 1 | -1 | -1 | -1 | -1 | -1 | -1 | -1 | -1 | -1 |
| smi_1091 | -1 | 1 | -1 | -1 | -1 | -1 | -1 | -1 | -1 | -1 | -1 |
| smi_1092 | -1 | 1 | -1 | -1 | -1 | -1 | -1 | -1 | -1 | -1 | -1 |
| smi_1093 | -1 | 0 | -1 | -1 | -1 | -1 | -1 | -1 | -1 | -1 | -1 |
| smi_1094 | 0 | 1 | -1 | -1 | -1 | -1 | -1 | -1 | -1 | -1 | -1 |
| smi_1095 | -1 | 1 | -1 | -1 | -1 | -1 | -1 | -1 | -1 | -1 | -1 |
| smi_1096 | -1 | 1 | -1 | -1 | -1 | -1 | -1 | -1 | -1 | -1 | -1 |
| smi_1097 | -1 | 1 | -1 | -1 | -1 | -1 | -1 | -1 | -1 | -1 | -1 |
| smi_1098 | -1 | 1 | -1 | -1 | -1 | -1 | -1 | -1 | -1 | -1 | -1 |
| smi_1099 | -1 | 1 | -1 | -1 | -1 | -1 | -1 | -1 | -1 | -1 | -1 |
| smi_1100 | -1 | -1 | -1 | -1 | -1 | -1 | -1 | -1 | -1 | -1 | -1 |
| smi_1101 | -1 | 1 | 1 | 1 | -1 | -1 | -1 | -1 | 1 | -1 | 1 |
| smi_1102 | 0 | 1 | 1 | 1 | -1 | -1 | -1 | -1 | 1 | -1 | 1 |
| smi_1103 | -1 | 1 | -1 | -1 | -1 | -1 | -1 | -1 | -1 | -1 | 1 |
| smi_1104 | 1 | 1 | 1 | 1 | 1 | 1 | 1 | 1 | 1 | 1 | -1 |
| smi_1105 | 1 | 1 | 1 | 1 | 1 | 1 | 1 | 1 | 1 | 1 | 1 |
| smi_1106 | 1 | -1 | 1 | 1 | 1 | 1 | -1 | 1 | -1 | -1 | 1 |
| smi_1107 | 1 | 1 | 1 | -1 | -1 | 1 | -1 | 1 | -1 | -1 | -1 |
| smi_1108 | -1 | -1 | -1 | -1 | -1 | -1 | -1 | 1 | -1 | -1 | -1 |
| smi_1109 | -1 | -1 | -1 | -1 | -1 | -1 | -1 | -1 | -1 | -1 | -1 |
| smi_1110 | 1 | 1 | -1 | -1 | -1 | -1 | -1 | -1 | -1 | -1 | -1 |
| smi_1111 | 1 | 1 | -1 | -1 | -1 | -1 | -1 | -1 | -1 | -1 | -1 |
| smi_1112 | -1 | -1 | -1 | -1 | -1 | -1 | -1 | -1 | -1 | -1 | -1 |
| smi_1113 | -1 | -1 | -1 | -1 | -1 | -1 | -1 | -1 | -1 | -1 | -1 |
| smi_1114 | -1 | -1 | -1 | -1 | -1 | 1 | -1 | 1 | -1 | -1 | -1 |
| smi_1115 | -1 | -1 | -1 | -1 | -1 | 1 | -1 | 1 | -1 | -1 | -1 |
| smi_1116 | -1 | -1 | -1 | -1 | 1 | 1 | -1 | 1 | -1 | -1 | 1 |
| smi_1117 | -1 | -1 | -1 | -1 | 1 | 1 | -1 | 1 | -1 | -1 | 1 |
| smi_1118 | -1 | -1 | -1 | -1 | -1 | -1 | -1 | -1 | -1 | -1 | -1 |
| smi_1119 | 1 | 1 | 1 | 1 | 1 | 1 | 1 | 1 | 1 | 1 | 1 |
| smi_1120 | 1 | 1 | 1 | 1 | 1 | 1 | 1 | 1 | 1 | 1 | 1 |
| smi_1121 | 1 | 1 | 1 | 1 | 1 | 1 | 1 | 1 | 1 | 1 | 1 |
| smi_1122 | 1 | 1 | 1 | 1 | 1 | 1 | 1 | 1 | 1 | 1 | 1 |
| smi_1123 | 1 | 1 | 1 | 1 | 1 | 1 | 1 | 1 | 1 | 1 | 1 |
| smi_1124 | 1 | 1 | 1 | 1 | 1 | 1 | 1 | 1 | 1 | 1 | 1 |
| smi_1125 | 1 | 1 | 1 | 1 | 1 | 1 | 1 | 1 | 1 | 1 | 1 |
| smi_1126 | 1 | 1 | 1 | 1 | 1 | 1 | 1 | 1 | 1 | 1 | 1 |
| smi_1127 | 1 | 1 | 1 | 1 | 1 | 1 | 1 | 1 | 1 | 1 | 1 |
| smi_1128 | 1 | -1 | 1 | 1 | -1 | -1 | -1 | -1 | -1 | 1 | 1 |
| smi_1129 | 1 | 1 | 1 | 1 | 1 | 1 | 1 | 1 | 1 | 1 | 1 |
| smi_1130 | 1 | 1 | 1 | 1 | 1 | 1 | 1 | 1 | 1 | 1 | 1 |
| smi_1131 | 1 | 1 | 1 | 1 | 1 | 1 | 1 | 1 | 1 | 1 | 1 |
| smi_1132 | 1 | 1 | 1 | 1 | 1 | 1 | 1 | 1 | 1 | 1 | 1 |
| smi_1133 | 1 | 1 | 1 | 1 | 1 | 1 | 1 | 1 | 1 | 1 | 1 |
| smi_1134 | 1 | 1 | 1 | 1 | 1 | 1 | 1 | 1 | 1 | 1 | 1 |
| smi_1135 | 1 | 1 | 1 | 1 | 1 | 1 | 1 | 1 | 1 | 1 | 1 |
| smi_1136 | 1 | 1 | 1 | -1 | -1 | 1 | 1 | -1 | 1 | 1 | -1 |
| smi_1137 | 1 | 1 | 1 | 1 | 1 | 1 | 1 | -1 | 1 | 1 | -1 |
| smi_1138 | 1 | 1 | 1 | 1 | 1 | 1 | 1 | 1 | 1 | 1 | 1 |
| smi_1139 | 1 | 1 | 1 | 1 | 1 | 1 | 1 | -1 | 1 | 1 | 1 |
| smi_1140 | -1 | -1 | -1 | 1 | -1 | -1 | -1 | 1 | -1 | -1 | 1 |
| smi_1141 | 1 | -1 | -1 | 1 | -1 | 1 | -1 | 1 | 1 | 1 | 1 |
| smi_1142 | 1 | 1 | 1 | -1 | 1 | -1 | -1 | -1 | -1 | -1 | -1 |
| smi_1143 | 1 | 1 | 1 | 1 | 1 | 1 | 1 | 1 | 1 | 1 | 1 |
| smi_1144 | 1 | 1 | 1 | 1 | 1 | 1 | 1 | 1 | -1 | 0 | 1 |
| smi_1145 | 1 | 1 | 1 | 1 | 1 | 1 | 1 | 1 | 1 | 1 | 1 |
| smi_1146 | 1 | 1 | 1 | 1 | 1 | 1 | 1 | 1 | 1 | 1 | 1 |
| smi_1147 | -1 | 1 | 1 | -1 | -1 | -1 | -1 | 1 | -1 | -1 | 1 |
| smi_1149 | 1 | 1 | 1 | 1 | -1 | 1 | 1 | 1 | 1 | 1 | -1 |
| smi_1150 | 1 | 1 | 1 | 1 | 1 | 1 | 1 | 1 | 1 | 1 | 1 |
| smi_1151 | 1 | 1 | 1 | 1 | 1 | 1 | 1 | 1 | 1 | 1 | 1 |
| smi_1152 | 1 | 1 | 1 | 1 | 1 | 1 | 1 | 1 | 1 | 1 | 1 |
| smi_1153 | 1 | 1 | 1 | 1 | 1 | 1 | -1 | 1 | -1 | -1 | 1 |
| smi_1154 | 1 | 1 | 1 | 1 | 1 | 0 | 1 | 1 | 1 | 1 | 1 |
| smi_1155 | 1 | 1 | 1 | 1 | 1 | 1 | 1 | 1 | 1 | 1 | 1 |
| smi_1156 | 1 | 0 | 1 | 1 | -1 | 0 | 1 | 1 | 1 | 1 | 1 |
| smi_1157 | 1 | 1 | 1 | 1 | -1 | 1 | 1 | -1 | 1 | 1 | -1 |
| smi_1159 | 1 | 1 | 1 | 1 | 1 | 1 | 1 | 1 | 1 | 1 | 1 |
| smi_1160 | 1 | 1 | 1 | 1 | 1 | 1 | 1 | 1 | 1 | 1 | 1 |
| smi_1161 | 1 | 1 | 0 | 1 | -1 | 1 | -1 | -1 | -1 | 1 | 1 |
| smi_1162 | 1 | 1 | 1 | 1 | 1 | 1 | 1 | 1 | 1 | 1 | 1 |
| smi_1163 | 1 | 1 | 1 | 1 | 1 | 1 | 1 | 1 | 1 | 1 | 1 |
| smi_1164 | 1 | 1 | 1 | 1 | 1 | 1 | 1 | 1 | 1 | 1 | 1 |
| smi_1165 | 1 | 1 | 1 | 1 | 1 | 1 | 1 | 1 | 1 | 1 | 1 |
| smi_1166 | 1 | 1 | 1 | 0 | 1 | 1 | 1 | 1 | 1 | 1 | 0 |
| smi_1168 | 1 | 1 | 1 | 1 | 1 | 1 | 1 | 1 | 1 | 1 | 1 |
| smi_1169 | 1 | 1 | 1 | 1 | 1 | 1 | 1 | 1 | 1 | 1 | 1 |
| smi_1170 | 1 | 1 | 1 | 1 | -1 | -1 | -1 | 0 | -1 | -1 | 0 |
| smi_1171 | 1 | 1 | 1 | 1 | 1 | -1 | -1 | 1 | 1 | -1 | 1 |
| smi_1172 | 1 | 1 | 1 | 1 | 1 | 1 | 1 | 1 | 1 | 1 | 1 |
| smi_1173 | 1 | 1 | 1 | 1 | 1 | 1 | 1 | 1 | 1 | 1 | 1 |
| smi_1174 | 1 | 1 | 1 | 1 | 1 | 1 | 1 | 1 | 1 | 1 | 1 |
| smi_1175 | 1 | 1 | 1 | 1 | 1 | 1 | 1 | 1 | 1 | 1 | 1 |
| smi_1176 | 1 | 1 | 1 | 1 | 1 | 1 | 1 | 1 | 1 | 1 | 1 |
| smi_1177 | 1 | 1 | 1 | 1 | 1 | 1 | 1 | 1 | 1 | 1 | 1 |
| smi_1178 | 1 | 1 | 1 | 1 | 1 | 1 | 1 | 1 | 1 | 1 | 1 |
| smi_1179 | 1 | 1 | 1 | 1 | 1 | 1 | 1 | 1 | 1 | 1 | 1 |
| smi_1180 | 1 | 1 | 1 | 1 | 1 | 1 | 1 | 1 | 1 | 1 | 1 |
| smi_1181 | 1 | 1 | 1 | 1 | -1 | 1 | 1 | 1 | 1 | 1 | 1 |
| smi_1182 | 1 | 1 | 1 | 1 | 1 | 1 | 1 | 1 | 1 | 1 | 1 |
| smi_1183 | 1 | 1 | 1 | 1 | 1 | 0 | 1 | 1 | 1 | 1 | 1 |
| smi_1184 | 1 | 1 | 1 | 1 | 1 | -1 | 1 | -1 | 1 | 1 | -1 |
| smi_1185 | 1 | 1 | 1 | 1 | 1 | 1 | 1 | 1 | 1 | 1 | 1 |
| smi_1186 | 1 | 1 | 1 | 1 | 1 | 1 | 1 | 1 | 1 | 1 | 1 |
| smi_1187 | 1 | 1 | 1 | 1 | 1 | 1 | 1 | 1 | 1 | 1 | 0 |
| smi_1188 | 1 | 1 | 1 | 1 | 1 | 1 | 1 | 1 | 1 | 1 | 1 |
| smi_1189 | 1 | 1 | 1 | 1 | 1 | 1 | 1 | 1 | 1 | 1 | 1 |
| smi_1190 | 1 | 1 | 1 | 1 | 1 | 1 | 1 | 1 | 1 | 1 | 1 |
| smi_1191 | 1 | 1 | 1 | 1 | 1 | 1 | 1 | 1 | 1 | 1 | 1 |
| smi_1192 | 1 | 1 | 1 | 1 | 1 | 1 | 1 | 1 | 1 | 1 | 1 |
| smi_1193 | 1 | 1 | 1 | 1 | 1 | 0 | 1 | 1 | 1 | 1 | -1 |
| smi_1194 | 1 | 1 | 1 | 1 | 1 | 1 | 1 | 1 | 1 | 1 | 1 |
| smi_1195 | 1 | 1 | 1 | 1 | 1 | 0 | 1 | 1 | 1 | 1 | 1 |
| smi_1196 | 1 | 1 | 1 | 1 | 1 | 1 | 1 | 1 | 1 | 1 | 1 |
| smi_1197 | 1 | 1 | 1 | 1 | 1 | 1 | 1 | 1 | 1 | 1 | 1 |
| smi_1198 | 1 | 1 | 1 | 1 | 1 | 1 | 1 | 1 | 1 | 1 | 1 |
| smi_1199 | 1 | 1 | 1 | -1 | 1 | -1 | 1 | 1 | -1 | -1 | 1 |
| smi_1200 | -1 | 1 | -1 | -1 | -1 | -1 | -1 | -1 | -1 | -1 | 1 |
| smi_1201 | 1 | 1 | 1 | 1 | 1 | 1 | 1 | 1 | 1 | 1 | 1 |
| smi_1202 | 1 | 1 | 1 | 1 | 1 | 1 | 1 | 1 | 1 | 1 | 1 |
| smi_1203 | 1 | 1 | 1 | 1 | 1 | 1 | 1 | 1 | 1 | 1 | 1 |
| smi_1204 | 1 | 1 | 1 | 1 | 1 | 1 | 1 | 1 | 1 | 1 | 1 |
| smi_1205 | 1 | 1 | 1 | 1 | 1 | 1 | 1 | 1 | 1 | 1 | 1 |
| smi_1206 | 1 | 1 | 1 | 1 | -1 | 1 | 1 | 1 | 1 | 1 | 1 |
| smi_1207 | 1 | 1 | 1 | 1 | 1 | 0 | 1 | 1 | 1 | 1 | 1 |
| smi_1208 | 1 | 1 | -1 | 1 | 1 | 1 | 1 | 1 | 1 | 0 | 1 |
| smi_1209 | 1 | 1 | 1 | 1 | 1 | 1 | 1 | 1 | 1 | 1 | 1 |
| smi_1210 | 1 | 1 | 1 | 1 | 1 | 1 | 1 | 1 | 1 | 1 | 1 |
| smi_1211 | 1 | 1 | 1 | 1 | 1 | 1 | 1 | 1 | 1 | 1 | 1 |
| smi_1212 | 1 | 1 | 1 | 1 | 1 | 1 | 1 | 1 | 1 | 1 | 1 |
| smi_1213 | 1 | 1 | 1 | 1 | 1 | 0 | 1 | 1 | 1 | 1 | 1 |
| smi_1214 | 1 | 1 | 1 | 1 | 1 | 1 | 1 | 1 | 1 | 1 | 1 |
| smi_1215 | 1 | 1 | 1 | 1 | 1 | 1 | 1 | 1 | 1 | 1 | -1 |
| smi_1216 | 1 | 1 | 1 | 1 | 1 | 1 | 1 | 1 | 1 | 1 | 1 |
| smi_1217 | 1 | 1 | 1 | 1 | 1 | 1 | 1 | 1 | 1 | 1 | 1 |
| smi_1218 | 1 | 1 | 1 | 1 | 1 | 1 | 1 | -1 | 1 | 1 | 1 |
| smi_1219 | 1 | 1 | 1 | 1 | 1 | 1 | 1 | 1 | 1 | 1 | 1 |
| smi_1220 | 1 | 1 | 1 | 1 | 1 | 1 | 1 | 1 | 1 | 1 | 1 |
| smi_1221 | 1 | 1 | 1 | 1 | 1 | 1 | 1 | 1 | 1 | 1 | 1 |
| smi_1222 | 1 | 1 | 1 | 1 | 1 | 1 | 1 | 1 | 1 | 1 | 1 |
| smi_1223 | 1 | 1 | 1 | 1 | 1 | 1 | 1 | 1 | 1 | 1 | 1 |
| smi_1224 | 1 | 1 | 1 | 1 | 1 | 1 | 1 | 1 | 1 | 1 | 0 |
| smi_1225 | 1 | 1 | 1 | 1 | 1 | 1 | 1 | 1 | 1 | 1 | 1 |
| smi_1226 | 1 | 1 | 1 | 1 | 1 | 1 | 1 | 1 | 1 | 1 | 1 |
| smi_1227 | 1 | 1 | 1 | 1 | 1 | 1 | 1 | 1 | 1 | 1 | 1 |
| smi_1228 | 1 | 1 | 1 | 1 | 1 | 1 | 1 | 1 | 1 | 1 | 1 |
| smi_1229 | 1 | 1 | 1 | 1 | 1 | 1 | 1 | -1 | -1 | -1 | 1 |
| smi_1230 | 1 | 1 | 1 | 1 | 1 | 1 | 1 | -1 | -1 | -1 | 1 |
| smi_1231 | 1 | 1 | 1 | 1 | 1 | 1 | 1 | -1 | -1 | -1 | 1 |
| smi_1232 | 1 | -1 | -1 | -1 | -1 | -1 | -1 | -1 | -1 | -1 | -1 |
| smi_1233 | 1 | 1 | 1 | 1 | 1 | 1 | 1 | 1 | 1 | 1 | 1 |
| smi_1234 | 1 | 1 | 1 | 1 | 1 | 1 | 1 | 1 | 1 | 1 | 1 |
| smi_1235 | 1 | 1 | 1 | 1 | 1 | 1 | 1 | 1 | 1 | 1 | 1 |
| smi_1236 | 1 | 1 | 1 | 1 | 1 | 1 | 1 | 1 | 1 | 1 | 1 |
| smi_1237 | 1 | 1 | 1 | 1 | 1 | 1 | 1 | 1 | 1 | 1 | 1 |
| smi_1238 | 1 | 1 | 1 | 1 | 1 | 1 | 1 | 1 | 1 | 1 | 1 |
| smi_1239 | 1 | 0 | -1 | -1 | 1 | 1 | 1 | 1 | 1 | 1 | 1 |
| smi_1240 | 1 | 1 | 1 | 1 | 1 | 1 | 1 | 1 | 1 | 1 | 1 |
| smi_1241 | 1 | 1 | -1 | 1 | 1 | 1 | 1 | 1 | 1 | 1 | 0 |
| smi_1242 | 1 | 1 | 1 | 1 | 1 | 1 | 1 | 1 | 1 | 1 | 1 |
| smi_1243 | 1 | 1 | 1 | 1 | 1 | 1 | 1 | 1 | 1 | 1 | 1 |
| smi_1244 | 1 | 1 | 1 | 1 | 1 | 1 | 1 | 1 | 1 | 1 | 1 |
| smi_1245 | -1 | -1 | -1 | -1 | -1 | -1 | -1 | -1 | -1 | -1 | -1 |
| smi_1246 | -1 | -1 | -1 | -1 | -1 | -1 | -1 | -1 | -1 | -1 | -1 |
| smi_1247 | -1 | -1 | -1 | -1 | -1 | -1 | -1 | -1 | -1 | -1 | -1 |
| smi_1248 | -1 | -1 | -1 | -1 | -1 | -1 | -1 | -1 | -1 | -1 | -1 |
| smi_1249 | -1 | -1 | -1 | -1 | -1 | -1 | -1 | -1 | -1 | -1 | -1 |
| smi_1250 | -1 | -1 | -1 | -1 | -1 | -1 | -1 | -1 | -1 | -1 | -1 |
| smi_1251 | -1 | -1 | -1 | -1 | -1 | -1 | -1 | -1 | -1 | -1 | -1 |
| smi_1252 | 1 | 1 | 1 | 1 | 1 | 1 | 1 | 1 | 1 | 1 | 1 |
| smi_1253 | 1 | 1 | 1 | 1 | 1 | 1 | 1 | 1 | 1 | 1 | 1 |
| smi_1254 | 1 | 1 | 1 | 1 | 1 | 1 | 1 | 1 | 1 | 1 | 1 |
| smi_1255 | 1 | 1 | 1 | 1 | 1 | 1 | 1 | 1 | 1 | 1 | 1 |
| smi_1256 | 1 | 1 | 1 | 1 | 1 | 1 | 1 | 1 | 1 | 1 | 1 |
| smi_1257 | -1 | 1 | -1 | 1 | 1 | -1 | -1 | 1 | -1 | -1 | -1 |
| smi_1258 | 1 | 1 | -1 | 1 | 1 | -1 | -1 | 1 | -1 | -1 | -1 |
| smi_1259 | -1 | 1 | -1 | 1 | 1 | -1 | -1 | 1 | -1 | -1 | -1 |
| smi_1260 | -1 | -1 | -1 | -1 | 1 | -1 | -1 | -1 | -1 | -1 | -1 |
| smi_1261 | -1 | -1 | -1 | -1 | 1 | -1 | 1 | 0 | -1 | -1 | -1 |
| smi_1262 | -1 | -1 | -1 | -1 | 1 | -1 | 1 | -1 | -1 | -1 | -1 |
| smi_1263 | -1 | -1 | -1 | -1 | 1 | -1 | -1 | -1 | -1 | -1 | -1 |
| smi_1264 | 1 | 1 | 1 | 1 | 1 | 1 | 1 | 1 | 1 | 1 | 1 |
| smi_1265 | 1 | 1 | 1 | 1 | 1 | 1 | 1 | 1 | 1 | 1 | 1 |
| smi_1266 | 1 | 1 | 1 | 1 | 1 | 1 | 1 | -1 | 1 | 1 | 1 |
| smi_1267 | 1 | 1 | 1 | 1 | 1 | 1 | 1 | -1 | 1 | 1 | 1 |
| smi_1268 | 1 | 1 | 1 | 1 | 1 | 1 | 1 | -1 | 1 | 1 | 1 |
| smi_1269 | 1 | 1 | 1 | 1 | 1 | 1 | 1 | 1 | 1 | 1 | 1 |
| smi_1270 | 1 | 1 | 1 | 1 | 1 | 1 | 1 | 1 | 1 | 1 | 1 |
| smi_1271 | 1 | 1 | 1 | 1 | 1 | 1 | 1 | 1 | 1 | 1 | 1 |
| smi_1272 | -1 | -1 | -1 | 1 | -1 | -1 | -1 | 1 | -1 | -1 | -1 |
| smi_1273 | -1 | -1 | -1 | 1 | -1 | -1 | -1 | 1 | -1 | -1 | -1 |
| smi_1274 | 1 | 1 | -1 | -1 | 1 | -1 | 1 | 1 | 1 | 1 | -1 |
| smi_1275 | 1 | 1 | -1 | -1 | 1 | -1 | 1 | 1 | 1 | 1 | -1 |
| smi_1276 | 1 | 1 | -1 | -1 | 1 | -1 | 1 | 1 | 1 | 1 | -1 |
| smi_1277 | 1 | 1 | -1 | -1 | 1 | -1 | 1 | 1 | 1 | 1 | 1 |
| smi_1278 | 1 | 1 | -1 | -1 | 1 | -1 | 1 | 1 | 1 | 1 | -1 |
| smi_1279 | 1 | 1 | -1 | -1 | 1 | -1 | -1 | 1 | -1 | -1 | -1 |
| smi_1280 | 1 | 1 | -1 | -1 | 1 | -1 | -1 | -1 | -1 | -1 | -1 |
| smi_1281 | 1 | 1 | -1 | -1 | 1 | -1 | -1 | -1 | -1 | -1 | -1 |
| smi_1282 | 1 | 1 | -1 | -1 | 1 | -1 | -1 | 1 | -1 | -1 | -1 |
| smi_1283 | 1 | 1 | 1 | 1 | 1 | 1 | 1 | 1 | 1 | 1 | 1 |
| smi_1284 | 1 | 1 | 1 | 1 | 1 | 1 | 1 | 1 | 1 | 1 | -1 |
| smi_1285 | 1 | 1 | 1 | 1 | 1 | 1 | 1 | 1 | 1 | 1 | 1 |
| smi_1286 | 1 | 1 | 1 | 1 | 1 | 1 | 1 | 1 | 1 | 0 | 1 |
| smi_1287 | 1 | 1 | 1 | 1 | 1 | 1 | 1 | 1 | 1 | 1 | 1 |
| smi_1288 | 1 | 1 | 1 | 1 | 1 | 1 | 1 | 1 | 1 | 1 | 1 |
| smi_1289 | 1 | 1 | 1 | 1 | 1 | 1 | 1 | 1 | 1 | 1 | 1 |
| smi_1290 | 1 | 1 | 1 | 1 | 1 | 1 | 1 | 1 | 1 | 1 | 1 |
| smi_1291 | 1 | 1 | 1 | 1 | 1 | 1 | 1 | 1 | 1 | 1 | 1 |
| smi_1292 | 1 | 1 | 1 | 1 | 0 | 1 | 1 | 0 | 1 | 1 | 0 |
| smi_1293 | 1 | 1 | 1 | 1 | 1 | 1 | 1 | 1 | 1 | 1 | 1 |
| smi_1294 | 1 | 1 | 1 | 1 | 1 | -1 | 1 | 1 | 1 | 1 | 1 |
| smi_1295 | 1 | 1 | 1 | 1 | 1 | -1 | 1 | 1 | 1 | 1 | 1 |
| smi_1296 | 1 | 1 | 1 | 1 | 1 | 1 | 1 | 1 | 1 | 1 | 1 |
| smi_1297 | 1 | 1 | 1 | 1 | 1 | 1 | 1 | -1 | 1 | 1 | -1 |
| smi_1298 | 1 | 1 | 1 | 1 | 1 | 1 | 1 | 1 | 1 | 1 | 1 |
| smi_1299 | 1 | 1 | 1 | 1 | 1 | 1 | 1 | 1 | 1 | 1 | 1 |
| smi_1300 | 1 | 1 | 1 | 1 | 1 | 1 | 1 | 1 | 1 | 1 | 1 |
| smi_1301 | 1 | 1 | 1 | 1 | 1 | 1 | 1 | 1 | 1 | 1 | 1 |
| smi_1302 | 1 | 1 | 1 | 1 | -1 | -1 | -1 | 1 | -1 | -1 | -1 |
| smi_1303 | -1 | -1 | -1 | -1 | -1 | -1 | -1 | -1 | -1 | -1 | -1 |
| smi_1304 | 1 | 1 | 1 | -1 | 1 | 1 | 1 | 1 | 1 | 1 | 1 |
| smi_1305 | -1 | 0 | 1 | 1 | 1 | 1 | 1 | -1 | 1 | 1 | 1 |
| smi_1306 | -1 | -1 | 1 | 0 | -1 | -1 | -1 | -1 | -1 | -1 | -1 |
| smi_1307 | -1 | -1 | -1 | -1 | -1 | -1 | -1 | 1 | -1 | -1 | -1 |
| smi_1308 | 1 | 1 | 1 | 1 | 1 | 1 | 1 | 1 | 1 | 1 | 1 |
| smi_1309 | 1 | 1 | 1 | 1 | 1 | 1 | 1 | 1 | 1 | 1 | 1 |
| smi_1310 | 1 | 1 | 0 | 1 | 1 | 1 | 1 | 1 | 1 | 0 | 1 |
| smi_1311 | 1 | 0 | 1 | 1 | 1 | 1 | 1 | 1 | 1 | 1 | 1 |
| smi_1312 | 1 | 0 | 1 | 1 | 1 | 1 | 1 | 1 | 1 | 1 | 1 |
| smi_1313 | 1 | 1 | 1 | 1 | 1 | 1 | 1 | 1 | 1 | 1 | 1 |
| smi_1314 | 1 | 1 | 1 | 1 | 1 | -1 | 0 | 1 | -1 | -1 | -1 |
| smi_1315 | 1 | 1 | 1 | 1 | 1 | 1 | 1 | 1 | 1 | 1 | 1 |
| smi_1316 | 1 | 1 | 1 | 0 | 1 | 1 | 1 | 1 | 1 | 1 | 1 |
| smi_1317 | -1 | 1 | -1 | -1 | -1 | 1 | -1 | -1 | -1 | -1 | -1 |
| smi_1318 | -1 | -1 | -1 | -1 | -1 | -1 | -1 | -1 | -1 | -1 | -1 |
| smi_1319 | -1 | -1 | -1 | -1 | -1 | -1 | -1 | -1 | -1 | -1 | -1 |
| smi_1320 | 1 | -1 | -1 | -1 | -1 | -1 | -1 | -1 | -1 | -1 | -1 |
| smi_1321 | 1 | -1 | -1 | -1 | -1 | -1 | -1 | -1 | -1 | -1 | -1 |
| smi_1322 | 1 | -1 | -1 | -1 | -1 | -1 | -1 | -1 | -1 | -1 | -1 |
| smi_1323 | 1 | -1 | -1 | -1 | -1 | -1 | -1 | -1 | -1 | -1 | -1 |
| smi_1324 | 1 | -1 | 1 | 1 | -1 | -1 | 1 | -1 | -1 | -1 | -1 |
| smi_1325 | 1 | -1 | -1 | -1 | -1 | -1 | -1 | -1 | -1 | -1 | -1 |
| smi_1326 | 1 | -1 | -1 | -1 | -1 | -1 | -1 | -1 | -1 | -1 | -1 |
| smi_1327 | 1 | -1 | -1 | -1 | -1 | -1 | -1 | -1 | -1 | -1 | -1 |
| smi_1328 | 1 | 0 | -1 | 0 | -1 | -1 | -1 | 1 | -1 | -1 | 1 |
| smi_1329 | 1 | -1 | -1 | -1 | -1 | -1 | -1 | -1 | -1 | -1 | -1 |
| smi_1330 | 1 | -1 | 1 | 1 | 1 | -1 | 1 | -1 | 1 | -1 | -1 |
| smi_1331 | 1 | -1 | -1 | -1 | -1 | -1 | -1 | -1 | -1 | -1 | -1 |
| smi_1332 | 1 | -1 | -1 | -1 | -1 | -1 | -1 | -1 | -1 | -1 | -1 |
| smi_1333 | 1 | -1 | -1 | -1 | 0 | 0 | -1 | -1 | -1 | -1 | -1 |
| smi_1334 | 1 | 0 | 1 | 1 | 1 | -1 | 1 | -1 | 1 | -1 | -1 |
| smi_1335 | -1 | -1 | -1 | -1 | -1 | -1 | -1 | -1 | -1 | -1 | -1 |
| smi_1336 | 1 | -1 | -1 | -1 | -1 | -1 | -1 | -1 | -1 | -1 | -1 |
| smi_1337 | 1 | -1 | -1 | -1 | -1 | -1 | -1 | -1 | -1 | -1 | -1 |
| smi_1338 | 1 | -1 | 1 | 1 | -1 | -1 | 1 | -1 | -1 | -1 | -1 |
| smi_1339 | 1 | -1 | -1 | -1 | -1 | -1 | -1 | -1 | -1 | -1 | -1 |
| smi_1340 | 1 | -1 | -1 | -1 | -1 | -1 | -1 | -1 | -1 | -1 | -1 |
| smi_1341 | 1 | -1 | -1 | -1 | -1 | -1 | -1 | -1 | -1 | -1 | -1 |
| smi_1342 | 1 | 1 | 1 | 1 | 1 | 1 | 1 | 1 | 1 | 1 | 1 |
| smi_1343 | 1 | 1 | 1 | 1 | 1 | 1 | 1 | 0 | 1 | 1 | 1 |
| smi_1344 | 1 | 1 | 1 | 1 | 1 | 1 | 1 | 1 | 1 | 1 | 1 |
| smi_1345 | 1 | 1 | 1 | 1 | 1 | 1 | 1 | 1 | 1 | 1 | 1 |
| smi_1346 | 1 | 1 | 1 | 1 | 1 | 1 | 1 | 1 | 1 | 1 | 1 |
| smi_1347 | 1 | 1 | 1 | 1 | 1 | 1 | 1 | 1 | 1 | 1 | 1 |
| smi_1348 | 1 | 1 | -1 | 1 | -1 | -1 | -1 | -1 | 1 | 1 | 1 |
| smi_1349 | 1 | 1 | -1 | 1 | 1 | 1 | -1 | 1 | 1 | -1 | 1 |
| smi_1350 | 1 | 1 | 1 | 1 | 1 | 1 | 1 | 1 | 1 | 1 | 1 |
| smi_1351 | 1 | 1 | 1 | 1 | 1 | 1 | 1 | 1 | 1 | 1 | 1 |
| smi_1352 | 1 | 1 | 1 | 1 | 1 | 1 | 1 | 1 | 1 | 1 | 1 |
| smi_1353 | 1 | 0 | 1 | 1 | 1 | 1 | 1 | 1 | 1 | 1 | 1 |
| smi_1354 | 1 | 1 | 1 | 1 | 1 | 1 | 1 | 1 | 1 | 1 | 1 |
| smi_1355 | 1 | 1 | 1 | 1 | 1 | 1 | 1 | 1 | 1 | 1 | 1 |
| smi_1356 | 1 | 1 | 1 | 1 | 1 | 1 | 1 | 1 | 1 | 1 | 1 |
| smi_1357 | 1 | 1 | 1 | 1 | 1 | 1 | 1 | 1 | 1 | 1 | 1 |
| smi_1358 | 1 | 1 | 1 | 1 | 1 | 1 | 1 | 1 | 1 | 1 | 1 |
| smi_1359 | 1 | 1 | 1 | 1 | 1 | 1 | 1 | 1 | 1 | 1 | 1 |
| smi_1360 | 1 | 1 | 1 | 1 | 1 | 1 | 1 | 1 | 1 | 1 | 1 |
| smi_1361 | 1 | 1 | 1 | -1 | -1 | -1 | -1 | -1 | -1 | -1 | 1 |
| smi_1362 | 1 | 1 | -1 | -1 | -1 | -1 | -1 | -1 | -1 | -1 | -1 |
| smi_1363 | 0 | 0 | 0 | 0 | 0 | 0 | 0 | 0 | 0 | 0 | 0 |
| smi_1364 | 1 | -1 | -1 | -1 | 1 | 0 | 1 | -1 | 0 | 1 | 1 |
| smi_1365 | 1 | -1 | 1 | 1 | -1 | 1 | -1 | -1 | 1 | -1 | -1 |
| smi_1366 | 1 | 1 | -1 | -1 | -1 | -1 | -1 | -1 | -1 | -1 | -1 |
| smi_1367 | 1 | 1 | 1 | -1 | -1 | -1 | -1 | -1 | -1 | -1 | 0 |
| smi_1368 | 1 | 1 | -1 | -1 | 1 | -1 | -1 | -1 | -1 | -1 | -1 |
| smi_1369 | 1 | 1 | 1 | -1 | 1 | -1 | -1 | -1 | -1 | -1 | -1 |
| smi_1370 | 1 | 1 | 1 | -1 | 1 | -1 | -1 | -1 | -1 | -1 | -1 |
| smi_1371 | 1 | 1 | 1 | -1 | -1 | -1 | -1 | -1 | -1 | -1 | -1 |
| smi_1372 | 1 | 1 | -1 | -1 | -1 | -1 | -1 | -1 | -1 | -1 | -1 |
| smi_1374 | 1 | 1 | 1 | 1 | 1 | 1 | 1 | 1 | 1 | 1 | 1 |
| smi_1375 | 1 | 1 | 1 | 1 | 1 | 1 | 1 | 1 | 1 | 1 | 1 |
| smi_1376 | 1 | 1 | 1 | 1 | 1 | 1 | 1 | 1 | 1 | 1 | 1 |
| smi_1377 | 1 | 1 | 1 | 1 | 1 | 1 | 1 | 1 | 1 | 1 | 1 |
| smi_1378 | 1 | 1 | -1 | 1 | 1 | 1 | 1 | 1 | 1 | 1 | -1 |
| smi_1379 | 1 | 1 | 1 | 1 | 1 | 1 | 1 | 1 | 1 | 1 | 1 |
| smi_1380 | 1 | 1 | 1 | 1 | 1 | 1 | 1 | 1 | 1 | 1 | 1 |
| smi_1381 | 1 | 1 | 1 | 1 | 1 | 1 | 1 | 1 | 1 | 1 | 1 |
| smi_1382 | 1 | 1 | 1 | 1 | 1 | 1 | 1 | 1 | 1 | 1 | 1 |
| smi_1383 | 1 | 1 | 1 | 1 | 1 | 1 | 1 | 1 | 1 | 1 | 1 |
| smi_1384 | 1 | 1 | 1 | 1 | 1 | 1 | 1 | 1 | 1 | 1 | 1 |
| smi_1385 | 1 | 1 | 1 | 1 | 1 | 1 | 1 | 1 | 1 | 1 | 1 |
| smi_1386 | 1 | 1 | 1 | 1 | 1 | 1 | 1 | 1 | 1 | 1 | 1 |
| smi_1387 | 1 | 1 | 1 | 1 | 1 | 1 | 1 | 1 | 1 | 1 | 1 |
| smi_1388 | 1 | 1 | 1 | 1 | 1 | 1 | 1 | 1 | 1 | 1 | 1 |
| smi_1389 | 1 | 1 | 1 | 1 | 1 | 1 | 1 | 1 | 1 | 1 | 1 |
| smi_1390 | 1 | 1 | 1 | 1 | 1 | 1 | 1 | 1 | 1 | 1 | 1 |
| smi_1391 | 1 | 1 | 1 | 1 | 1 | 1 | 1 | 1 | 1 | 1 | 1 |
| smi_1392 | 1 | 1 | 1 | 1 | 1 | 1 | 1 | 1 | 1 | 1 | 1 |
| smi_1393 | -1 | -1 | -1 | -1 | -1 | -1 | -1 | -1 | -1 | -1 | -1 |
| smi_1394 | 1 | 1 | 1 | 1 | 1 | 1 | 1 | 1 | 1 | 1 | 1 |
| smi_1395 | 1 | 1 | 1 | 1 | 1 | 1 | 1 | 1 | 1 | 1 | 1 |
| smi_1396 | 1 | 1 | 1 | 1 | 1 | 1 | 1 | 1 | 1 | 1 | 1 |
| smi_1397 | -1 | -1 | -1 | -1 | -1 | -1 | -1 | -1 | -1 | -1 | -1 |
| smi_1398 | -1 | -1 | -1 | -1 | -1 | -1 | -1 | -1 | 1 | 1 | -1 |
| smi_1399 | 1 | 1 | 1 | 1 | 1 | 1 | 1 | 1 | 1 | 1 | 0 |
| smi_1400 | 1 | 1 | 1 | 1 | 1 | 1 | 1 | 1 | 1 | 1 | 1 |
| smi_1401 | 0 | 1 | -1 | -1 | -1 | -1 | 1 | 1 | 0 | 0 | -1 |
| smi_1402 | 1 | 1 | 1 | 1 | 1 | 1 | 1 | 1 | 1 | 1 | 1 |
| smi_1403 | 1 | 1 | 1 | 1 | 1 | 1 | 1 | 1 | 1 | 1 | 1 |
| smi_1404 | 1 | 1 | 1 | 1 | 1 | 1 | 1 | 1 | 1 | 1 | 1 |
| smi_1405 | -1 | -1 | -1 | -1 | -1 | -1 | -1 | -1 | -1 | -1 | -1 |
| smi_1406 | 1 | 1 | 1 | -1 | -1 | 1 | 1 | -1 | 1 | 1 | -1 |
| smi_1407 | 1 | 1 | 1 | 1 | 1 | 1 | 1 | 1 | 1 | 1 | 1 |
| smi_1408 | 1 | 1 | 1 | 1 | 1 | 1 | 1 | 1 | 1 | 1 | 0 |
| smi_1410 | 1 | 1 | 1 | 1 | 1 | 1 | 1 | 1 | 1 | 1 | 1 |
| smi_1411 | 1 | 1 | 1 | 1 | 1 | 1 | 1 | 1 | 1 | 1 | 1 |
| smi_1412 | 1 | 1 | 1 | 1 | 1 | 1 | 1 | 1 | 1 | 1 | 1 |
| smi_1413 | 1 | 1 | 1 | 1 | 1 | 1 | 1 | 1 | 1 | 1 | 1 |
| smi_1414 | 1 | 1 | 1 | 1 | 1 | 1 | 1 | 1 | 1 | 1 | 1 |
| smi_1415 | 1 | 1 | 1 | 1 | 1 | 1 | 1 | 1 | 1 | 1 | 1 |
| smi_1416 | 1 | 1 | 1 | 1 | 1 | 1 | 1 | 1 | 1 | 1 | 1 |
| smi_1417 | 1 | 1 | 1 | 1 | 1 | 1 | 1 | 1 | 1 | 1 | 1 |
| smi_1418 | 1 | 1 | 1 | 1 | 1 | 1 | 1 | 1 | 1 | 1 | 1 |
| smi_1420 | 1 | 1 | 1 | 1 | 1 | 1 | 1 | 1 | 1 | 1 | 1 |
| smi_1421 | 1 | 1 | 1 | 1 | 1 | 1 | 1 | 1 | 1 | 1 | 1 |
| smi_1422 | 1 | 1 | 1 | 1 | 1 | 1 | 1 | 1 | 1 | 1 | 1 |
| smi_1423 | 1 | 1 | 1 | 1 | 1 | 1 | 1 | 1 | 1 | 1 | 1 |
| smi_1424 | 1 | 1 | 1 | 1 | 1 | 1 | 1 | 1 | 1 | 1 | -1 |
| smi_1425 | 1 | 1 | 1 | 1 | 1 | 1 | 1 | 1 | 1 | 1 | 1 |
| smi_1426 | 1 | 1 | 1 | 1 | 1 | 1 | 1 | 1 | 1 | 1 | -1 |
| smi_1427 | 1 | 1 | 1 | 1 | 1 | 1 | 1 | 1 | 1 | 1 | 1 |
| smi_1428 | 1 | 1 | -1 | -1 | -1 | 1 | -1 | 1 | -1 | 1 | -1 |
| smi_1429 | 1 | 1 | -1 | -1 | -1 | -1 | -1 | -1 | -1 | -1 | -1 |
| smi_1430 | 1 | 1 | 1 | 1 | 1 | 1 | 1 | 1 | 1 | 1 | 1 |
| smi_1431 | 1 | 1 | 1 | 1 | 1 | 1 | 1 | 1 | 1 | 1 | 1 |
| smi_1432 | 1 | 1 | 1 | 1 | 1 | 1 | 1 | 1 | 1 | 1 | 1 |
| smi_1433 | 1 | 1 | 1 | 1 | 1 | 1 | 1 | 1 | 1 | 1 | 1 |
| smi_1434 | 1 | 1 | 1 | 1 | 1 | 1 | 1 | 1 | 1 | 1 | 1 |
| smi_1435 | 1 | 1 | 1 | 1 | 1 | 1 | 1 | 1 | 1 | 1 | 1 |
| smi_1436 | 1 | 1 | 1 | 1 | 1 | -1 | 1 | 1 | 1 | 1 | 1 |
| smi_1437 | 1 | 1 | 1 | 1 | 1 | -1 | 1 | 1 | 1 | 1 | 1 |
| smi_1438 | 1 | 1 | 1 | 1 | 1 | -1 | 1 | 1 | 1 | 1 | 1 |
| smi_1439 | 1 | 1 | 1 | 1 | 1 | -1 | 1 | 1 | 1 | 1 | 1 |
| smi_1440 | -1 | -1 | -1 | -1 | -1 | -1 | -1 | -1 | -1 | -1 | 1 |
| smi_1441 | -1 | -1 | -1 | -1 | -1 | -1 | -1 | -1 | -1 | -1 | 1 |
| smi_1442 | 0 | -1 | -1 | -1 | -1 | -1 | -1 | -1 | -1 | -1 | 1 |
| smi_1443 | -1 | -1 | -1 | -1 | -1 | -1 | -1 | -1 | -1 | -1 | 1 |
| smi_1444 | -1 | -1 | -1 | -1 | -1 | -1 | -1 | -1 | -1 | -1 | 1 |
| smi_1445 | 1 | 1 | 1 | 1 | 1 | 1 | 1 | 1 | 1 | 1 | 1 |
| smi_1446 | 1 | 1 | 1 | 1 | 1 | 1 | 1 | 1 | 1 | 1 | 1 |
| smi_1447 | 1 | 1 | 1 | 1 | 1 | 1 | 1 | 1 | 1 | 1 | 1 |
| smi_1448 | 1 | 1 | 1 | 1 | 1 | 1 | 1 | 1 | 1 | 1 | 1 |
| smi_1449 | 1 | 1 | 1 | 1 | 1 | 1 | 1 | 1 | 1 | 1 | 1 |
| smi_1450 | 1 | 1 | 1 | -1 | 1 | 1 | -1 | -1 | -1 | -1 | 1 |
| smi_1451 | 1 | 1 | -1 | -1 | 1 | 1 | -1 | -1 | -1 | -1 | 1 |
| smi_1453 | 1 | 1 | 1 | 1 | 1 | 1 | 1 | 1 | 1 | 1 | 1 |
| smi_1454 | 1 | 1 | 1 | 1 | 1 | 1 | 1 | 1 | 1 | 1 | 1 |
| smi_1455 | 1 | 1 | 1 | 1 | 1 | 1 | 1 | 1 | 1 | 1 | 1 |
| smi_1456 | 1 | 1 | 1 | 1 | 1 | 1 | 1 | 1 | 1 | 1 | 1 |
| smi_1457 | 1 | 1 | -1 | -1 | 1 | 1 | -1 | 1 | -1 | -1 | -1 |
| smi_1458 | 1 | 1 | -1 | -1 | 1 | 1 | -1 | 1 | -1 | -1 | -1 |
| smi_1459 | 1 | 1 | -1 | -1 | 1 | 1 | -1 | 1 | -1 | -1 | -1 |
| smi_1460 | 1 | 1 | -1 | -1 | 1 | 1 | -1 | 1 | -1 | -1 | -1 |
| smi_1461 | 1 | 1 | 1 | 1 | 1 | 1 | 1 | 1 | 1 | 1 | 1 |
| smi_1462 | 0 | -1 | -1 | -1 | -1 | 1 | -1 | -1 | -1 | -1 | -1 |
| smi_1463 | -1 | 1 | -1 | -1 | -1 | -1 | -1 | -1 | -1 | -1 | -1 |
| smi_1464 | -1 | 1 | -1 | -1 | -1 | -1 | -1 | -1 | -1 | -1 | -1 |
| smi_1465 | 1 | 1 | -1 | -1 | -1 | 1 | -1 | -1 | -1 | -1 | -1 |
| smi_1466 | 1 | 1 | -1 | -1 | -1 | 1 | -1 | -1 | -1 | -1 | -1 |
| smi_1467 | 1 | 1 | -1 | -1 | -1 | 1 | -1 | -1 | -1 | -1 | -1 |
| smi_1468 | 1 | 1 | -1 | -1 | -1 | -1 | -1 | -1 | -1 | -1 | -1 |
| smi_1469 | 1 | 1 | -1 | -1 | -1 | -1 | -1 | -1 | -1 | -1 | -1 |
| smi_1470 | 1 | 1 | -1 | -1 | -1 | 1 | -1 | -1 | -1 | -1 | -1 |
| smi_1471 | 1 | 1 | 1 | 1 | 1 | 0 | 0 | 0 | 1 | 1 | 1 |
| smi_1472 | 1 | 1 | 1 | 1 | 1 | 1 | 1 | 1 | 1 | 1 | 1 |
| smi_1473 | 1 | 1 | 0 | 1 | 1 | 1 | 1 | -1 | 1 | 0 | 0 |
| smi_1474 | 1 | 1 | 1 | 1 | -1 | 1 | 0 | 1 | 1 | 1 | 1 |
| smi_1475 | 1 | 1 | 1 | 1 | 1 | 1 | 1 | 1 | 1 | 1 | 1 |
| smi_1476 | 1 | 1 | 1 | 1 | 1 | 1 | 1 | 1 | 1 | 1 | 1 |
| smi_1477 | 1 | 1 | 1 | 1 | 1 | 1 | 1 | 1 | 1 | 1 | 1 |
| smi_1478 | 1 | 1 | 1 | 1 | 1 | 1 | 1 | 1 | 1 | 1 | 1 |
| smi_1479 | 1 | 1 | 1 | 1 | 1 | 1 | 1 | 1 | -1 | 1 | 1 |
| smi_1480 | -1 | -1 | 1 | 1 | 1 | 1 | 1 | 1 | -1 | -1 | -1 |
| smi_1481 | 1 | -1 | -1 | 1 | 1 | -1 | -1 | 1 | -1 | -1 | 1 |
| smi_1482 | 1 | 1 | -1 | 1 | -1 | 1 | -1 | -1 | -1 | -1 | -1 |
| smi_1483 | 1 | 1 | 1 | 1 | 1 | 1 | 1 | 1 | 1 | 1 | 1 |
| smi_1484 | 1 | 1 | 1 | 1 | 1 | 1 | 1 | 1 | 1 | 1 | 1 |
| smi_1485 | 1 | 1 | 1 | 1 | 1 | 1 | 1 | 1 | 1 | 1 | 0 |
| smi_1486 | 1 | 1 | 1 | 1 | 1 | 1 | 1 | 1 | 1 | 1 | 1 |
| smi_1487 | 1 | 1 | 1 | 1 | 1 | 1 | 1 | 1 | 1 | 1 | 1 |
| smi_1488 | 1 | 1 | 1 | 1 | 1 | 1 | 1 | 1 | 1 | 1 | 1 |
| smi_1489 | 1 | 1 | 1 | 1 | 1 | 1 | -1 | 1 | -1 | -1 | 1 |
| smi_1490 | 1 | 1 | 1 | 1 | 0 | 1 | 1 | 1 | 1 | -1 | 1 |
| smi_1491 | 1 | 1 | 1 | 1 | 1 | 1 | 1 | 1 | 1 | 1 | 1 |
| smi_1492 | 1 | 1 | 1 | 1 | 1 | 1 | 1 | 1 | 1 | 1 | 1 |
| smi_1493 | -1 | 1 | 1 | -1 | 1 | 1 | -1 | 1 | 1 | 1 | 1 |
| smi_1494 | -1 | 1 | 1 | -1 | 1 | 1 | -1 | 1 | 1 | 1 | 1 |
| smi_1495 | 1 | 1 | 1 | 1 | 1 | 0 | 1 | -1 | 1 | 1 | 1 |
| smi_1496 | 1 | 1 | 1 | 1 | 1 | 1 | 1 | -1 | 1 | 1 | 1 |
| smi_1497 | 1 | 1 | 1 | 1 | 1 | 1 | 1 | 1 | 1 | 1 | -1 |
| smi_1498 | 1 | 1 | 1 | 1 | 1 | 1 | 1 | 1 | 1 | 1 | 1 |
| smi_1499 | 1 | 1 | 1 | -1 | -1 | 1 | 1 | -1 | 1 | 1 | -1 |
| smi_1500 | 1 | 1 | 1 | 1 | 1 | 1 | 1 | 1 | 1 | 1 | 1 |
| smi_1501 | 1 | 1 | 1 | 1 | 1 | 1 | 1 | 1 | 1 | 1 | 1 |
| smi_1502 | 1 | 1 | 1 | 1 | 1 | 1 | 1 | 1 | 1 | 1 | 1 |
| smi_1503 | 1 | 1 | 1 | -1 | -1 | 1 | 1 | -1 | 1 | 1 | -1 |
| smi_1504 | 1 | 1 | 1 | 1 | 1 | 1 | 1 | 1 | 1 | 1 | 1 |
| smi_1505 | 1 | -1 | -1 | -1 | -1 | -1 | -1 | -1 | -1 | -1 | -1 |
| smi_1506 | 1 | -1 | -1 | -1 | -1 | -1 | -1 | -1 | -1 | -1 | -1 |
| smi_1507 | 1 | 1 | 1 | 1 | 1 | 1 | 1 | 1 | 1 | 1 | 1 |
| smi_1508 | 1 | 1 | 1 | 1 | 1 | 1 | 1 | 1 | 1 | 1 | 1 |
| smi_1509 | 1 | 1 | 1 | 1 | 1 | 1 | 1 | 1 | -1 | -1 | -1 |
| smi_1510 | 1 | 0 | 1 | 1 | -1 | -1 | 1 | 1 | -1 | -1 | -1 |
| smi_1511 | 1 | 1 | 1 | 1 | 1 | 1 | -1 | 1 | -1 | -1 | 1 |
| smi_1512 | 1 | 1 | 1 | 1 | 1 | 1 | 1 | 1 | 1 | 1 | 1 |
| smi_1513 | 1 | 1 | 1 | 1 | 1 | 1 | 1 | 1 | 1 | 1 | 1 |
| smi_1514 | 1 | 1 | 1 | 1 | 1 | 1 | 1 | 1 | 1 | 1 | 1 |
| smi_1515 | 1 | 1 | 1 | 1 | 1 | 1 | 1 | 1 | 1 | 1 | 0 |
| smi_1516 | 1 | 1 | 1 | 1 | 1 | 1 | 1 | 1 | 1 | 1 | 1 |
| smi_1517 | 1 | 1 | 1 | 1 | 1 | 1 | 1 | 1 | 1 | 1 | 1 |
| smi_1518 | 1 | 1 | 1 | 1 | 1 | 1 | 1 | 1 | 1 | 1 | 1 |
| smi_1519 | 1 | 1 | 1 | 1 | 1 | 1 | 1 | 1 | 1 | 1 | 1 |
| smi_1520 | 1 | 1 | 1 | 1 | 1 | 1 | 1 | 1 | 1 | 1 | 1 |
| smi_1521 | 1 | 1 | 1 | 1 | 1 | 1 | 1 | 1 | 1 | 1 | 1 |
| smi_1522 | 1 | 1 | 1 | 1 | 1 | 1 | 1 | 1 | 1 | 1 | 1 |
| smi_1523 | 1 | 1 | 1 | 1 | 1 | 1 | 1 | 1 | 1 | 1 | 1 |
| smi_1525 | 1 | 1 | 1 | 1 | 1 | 1 | 1 | 1 | 1 | 1 | 1 |
| smi_1526 | 1 | 1 | -1 | 1 | 1 | -1 | -1 | -1 | -1 | -1 | 1 |
| smi_1527 | 1 | 1 | 0 | 1 | 1 | 1 | 1 | 1 | 1 | 1 | 1 |
| smi_1528 | 1 | 1 | 1 | 1 | 1 | 1 | 1 | 1 | 1 | 1 | 1 |
| smi_1529 | 1 | 1 | 1 | 1 | 1 | 1 | -1 | 1 | 1 | 1 | -1 |
| smi_1530 | 1 | 1 | 1 | 1 | 1 | 1 | 1 | 1 | 1 | 1 | 1 |
| smi_1531 | 1 | 1 | 1 | 1 | -1 | -1 | -1 | -1 | 1 | 1 | 1 |
| smi_1532 | 1 | 1 | 0 | 1 | 0 | 1 | 1 | -1 | 1 | 1 | 1 |
| smi_1533 | 1 | 1 | 1 | 1 | 1 | 1 | 1 | 1 | 1 | 1 | 1 |
| smi_1534 | -1 | -1 | 0 | -1 | 0 | -1 | -1 | -1 | -1 | 0 | -1 |
| smi_1535 | -1 | 1 | 1 | 1 | 1 | 1 | 1 | -1 | 1 | 1 | 1 |
| smi_1536 | -1 | -1 | -1 | -1 | -1 | -1 | -1 | -1 | -1 | -1 | -1 |
| smi_1537 | -1 | -1 | -1 | -1 | -1 | -1 | -1 | -1 | -1 | -1 | -1 |
| smi_1538 | -1 | -1 | -1 | -1 | -1 | -1 | -1 | -1 | -1 | -1 | -1 |
| smi_1539 | 1 | 1 | 1 | 1 | 1 | 1 | 1 | 1 | 1 | 1 | 1 |
| smi_1540 | -1 | 1 | 1 | 1 | 1 | 1 | -1 | 1 | 1 | -1 | 1 |
| smi_1541 | 1 | 1 | 1 | 1 | 1 | 1 | 1 | 1 | 1 | 1 | 1 |
| smi_1542 | 1 | 1 | 1 | 1 | 1 | 1 | 1 | 1 | 1 | 1 | 1 |
| smi_1543 | 1 | 1 | 1 | 1 | 1 | 1 | 1 | 1 | 1 | 1 | 1 |
| smi_1544 | 1 | 1 | 1 | 1 | 1 | 1 | 1 | 1 | 1 | 1 | 1 |
| smi_1545 | 1 | 1 | 1 | 1 | 1 | 1 | 1 | 1 | 1 | 1 | 1 |
| smi_1546 | 1 | 1 | 1 | 1 | 1 | 1 | 1 | 1 | 1 | 1 | 1 |
| smi_1547 | 1 | 1 | 1 | 1 | 1 | 1 | 1 | 1 | 1 | 1 | 1 |
| smi_1548 | 1 | 1 | 1 | 1 | 1 | 1 | 1 | 1 | 1 | 1 | 1 |
| smi_1549 | 1 | 1 | 1 | 1 | 1 | 1 | 1 | 1 | 1 | 1 | 1 |
| smi_1550 | 1 | 1 | 1 | 1 | 1 | 1 | 1 | 1 | 1 | 1 | 1 |
| smi_1551 | 1 | 1 | 1 | 1 | 0 | 0 | 1 | 1 | 1 | 1 | 1 |
| smi_1552 | 1 | 1 | 1 | 1 | 1 | 1 | 1 | 1 | 1 | 1 | 1 |
| smi_1553 | 1 | 1 | 1 | 1 | 1 | 1 | 1 | 1 | 1 | 1 | 1 |
| smi_1554 | 1 | 1 | 1 | 1 | 1 | 1 | 1 | 1 | 1 | 1 | 1 |
| smi_1555 | 1 | 1 | 1 | 1 | 1 | 1 | 1 | 1 | 1 | 1 | 1 |
| smi_1556 | 1 | 1 | 1 | 1 | 1 | 1 | 1 | 1 | 1 | 1 | 1 |
| smi_1557 | 1 | 1 | 1 | 1 | 1 | 1 | 1 | 1 | 1 | 1 | 1 |
| smi_1558 | 1 | 1 | 1 | 1 | 1 | 1 | 1 | 1 | 1 | 1 | 1 |
| smi_1559 | 1 | 1 | 1 | 1 | 1 | 1 | 1 | 1 | 1 | 1 | 1 |
| smi_1560 | 1 | 1 | 1 | 1 | 1 | 1 | 1 | 1 | 1 | 1 | 1 |
| smi_1561 | 1 | 1 | 1 | 1 | 1 | 0 | 1 | 1 | 1 | 1 | 1 |
| smi_1562 | 0 | 1 | 1 | 0 | 1 | 1 | 1 | 1 | 0 | 1 | 0 |
| smi_1563 | 1 | 1 | 1 | 1 | -1 | 1 | -1 | -1 | -1 | -1 | -1 |
| smi_1564 | 1 | 1 | 1 | 1 | 1 | 1 | 1 | 1 | 1 | 1 | 1 |
| smi_1565 | 1 | 1 | 1 | 1 | 1 | 1 | 1 | 1 | 1 | 1 | 1 |
| smi_1566 | 1 | 1 | 1 | 1 | 1 | 1 | 1 | 1 | 1 | 1 | 1 |
| smi_1567 | 1 | 1 | 1 | 1 | 1 | 1 | 1 | 1 | 1 | 1 | 1 |
| smi_1568 | -1 | -1 | 1 | 1 | 1 | -1 | 1 | -1 | 1 | 1 | 1 |
| smi_1569 | 1 | 1 | 1 | 1 | 1 | 1 | 1 | 1 | 1 | 1 | 1 |
| smi_1570 | 1 | 1 | 1 | 1 | 1 | 1 | 1 | 1 | 1 | 1 | 1 |
| smi_1571 | 1 | 1 | 1 | 1 | 1 | 1 | 1 | 1 | 1 | 1 | 1 |
| smi_1572 | 1 | 1 | 1 | 1 | 1 | 1 | 1 | 0 | 1 | 1 | 1 |
| smi_1573 | 1 | 1 | 1 | 1 | 1 | 1 | 1 | 1 | 1 | 1 | 1 |
| smi_1574 | 1 | 1 | 1 | 1 | 1 | 1 | 1 | 1 | 1 | 1 | 1 |
| smi_1575 | 1 | 1 | 1 | 1 | 1 | 1 | 1 | 1 | -1 | 1 | 1 |
| smi_1576 | 1 | 1 | 1 | 1 | 1 | 1 | 1 | 1 | 1 | 1 | -1 |
| smi_1577 | 1 | 1 | 1 | 1 | 1 | 1 | 1 | 1 | 1 | 1 | 1 |
| smi_1578 | 1 | 1 | 1 | 1 | 1 | 1 | 1 | 1 | 1 | 1 | 1 |
| smi_1579 | 1 | 1 | 1 | 1 | -1 | 1 | -1 | 1 | -1 | -1 | 1 |
| smi_1580 | 1 | -1 | 1 | 1 | 1 | -1 | 1 | 1 | -1 | 1 | 1 |
| smi_1581 | 1 | 1 | 1 | 1 | 1 | 1 | 1 | 1 | 1 | 1 | 1 |
| smi_1582 | 1 | 1 | 1 | 1 | 1 | 1 | 1 | 1 | 1 | 1 | 1 |
| smi_1583 | 1 | 1 | 1 | 1 | 1 | 0 | -1 | 1 | -1 | -1 | 1 |
| smi_1584 | 1 | 1 | 1 | 1 | 1 | 1 | -1 | 0 | -1 | -1 | 1 |
| smi_1585 | 1 | 1 | 1 | 1 | 1 | 1 | 1 | 1 | 1 | 1 | 1 |
| smi_1586 | 1 | 1 | 1 | 1 | 1 | 1 | 1 | 1 | 1 | 1 | 1 |
| smi_1587 | 1 | 1 | 1 | 1 | 1 | 1 | 1 | 1 | 1 | 1 | 1 |
| smi_1588 | 1 | 1 | 1 | 1 | 1 | 1 | 1 | 1 | 1 | 1 | 1 |
| smi_1589 | 1 | 1 | 1 | 1 | 1 | 1 | 1 | 1 | 1 | 1 | 1 |
| smi_1590 | 1 | 1 | 1 | 1 | 1 | 1 | 1 | 0 | 1 | 1 | 1 |
| smi_1591 | 1 | 1 | 1 | 1 | 1 | 1 | 1 | 1 | 1 | 1 | 1 |
| smi_1592 | 1 | 1 | 1 | 1 | 1 | 1 | 1 | 1 | 1 | 1 | 1 |
| smi_1593 | 1 | 1 | 1 | 1 | 1 | 1 | 1 | 1 | 1 | 1 | 1 |
| smi_1594 | 1 | 1 | 1 | 1 | 1 | 1 | 1 | 1 | 1 | 1 | 1 |
| smi_1595 | 1 | 1 | 1 | 1 | 1 | 1 | 1 | 1 | 1 | 1 | 1 |
| smi_1596 | 1 | 1 | 1 | 1 | 1 | 1 | 1 | 1 | -1 | -1 | 0 |
| smi_1597 | 1 | 1 | 1 | 1 | 1 | 1 | 1 | 1 | 1 | 0 | 1 |
| smi_1598 | 1 | 1 | 1 | 1 | 1 | 1 | 1 | 1 | 1 | 1 | 1 |
| smi_1599 | 1 | 1 | 1 | 1 | 1 | 1 | 1 | 1 | 1 | 1 | 1 |
| smi_1600 | 1 | 1 | 1 | 1 | 1 | 1 | 1 | 1 | 1 | 1 | 1 |
| smi_1601 | 1 | 1 | 0 | -1 | -1 | -1 | 0 | 0 | -1 | -1 | -1 |
| smi_1602 | 1 | 1 | 1 | 1 | -1 | 1 | 1 | -1 | 1 | 1 | -1 |
| smi_1603 | -1 | 1 | 0 | -1 | -1 | 1 | 1 | -1 | 1 | 1 | -1 |
| smi_1604 | 1 | -1 | 1 | -1 | -1 | -1 | -1 | -1 | 1 | -1 | -1 |
| smi_1605 | 1 | -1 | 1 | -1 | -1 | -1 | -1 | -1 | 1 | -1 | -1 |
| smi_1606 | -1 | -1 | -1 | -1 | -1 | -1 | -1 | -1 | -1 | -1 | -1 |
| smi_1607 | 1 | -1 | 1 | -1 | -1 | -1 | 0 | -1 | 1 | 0 | 0 |
| smi_1608 | 1 | -1 | -1 | -1 | -1 | -1 | -1 | -1 | 1 | -1 | -1 |
| smi_1609 | 1 | -1 | 1 | -1 | -1 | -1 | -1 | -1 | 1 | -1 | -1 |
| smi_1610 | -1 | -1 | -1 | -1 | -1 | 1 | -1 | -1 | -1 | -1 | -1 |
| smi_1611 | -1 | -1 | -1 | -1 | -1 | 1 | -1 | -1 | -1 | -1 | -1 |
| smi_1612 | -1 | -1 | 1 | 1 | -1 | 1 | -1 | -1 | -1 | -1 | -1 |
| smi_1613 | 1 | 1 | 1 | 0 | 1 | 1 | 1 | 1 | 1 | 1 | 1 |
| smi_1614 | 1 | 1 | 1 | 1 | 1 | 1 | 1 | 1 | 1 | 1 | 1 |
| smi_1615 | 1 | 1 | 1 | 1 | 1 | 1 | 1 | 1 | 1 | 1 | 1 |
| smi_1616 | 1 | 1 | 1 | 1 | 1 | 1 | 1 | 1 | 1 | 1 | 1 |
| smi_1617 | 1 | 1 | 1 | 1 | 1 | 1 | 1 | 1 | 1 | 1 | 1 |
| smi_1618 | 1 | 1 | 1 | 1 | 1 | 1 | 1 | 1 | 1 | 1 | 1 |
| smi_1619 | 1 | 1 | 1 | 1 | 1 | 1 | 1 | 1 | 1 | 1 | 1 |
| smi_1620 | 1 | 1 | 1 | 1 | 1 | 1 | 1 | 1 | 1 | 1 | 1 |
| smi_1621 | 1 | 1 | 1 | 1 | 1 | 1 | 1 | 1 | 1 | 1 | 1 |
| smi_1622 | 1 | 1 | 1 | 1 | 1 | 1 | 1 | 1 | 1 | 1 | 1 |
| smi_1623 | 1 | 1 | 1 | 1 | 1 | 1 | 1 | 1 | 1 | 1 | 1 |
| smi_1624 | 1 | 1 | 1 | 1 | 1 | 0 | 1 | 1 | 1 | 1 | 1 |
| smi_1625 | 1 | 1 | 1 | 1 | 1 | 1 | 1 | 1 | 1 | 1 | 1 |
| smi_1626 | 1 | 1 | 1 | 1 | 1 | 0 | 1 | 1 | 1 | 1 | 1 |
| smi_1627 | 1 | 1 | 1 | 1 | 1 | 1 | 1 | 1 | 1 | 1 | 1 |
| smi_1628 | 1 | 1 | 1 | 1 | 1 | 1 | 1 | 1 | 1 | 1 | 1 |
| smi_1629 | 1 | 1 | -1 | -1 | -1 | -1 | -1 | -1 | -1 | -1 | -1 |
| smi_1630 | 1 | 1 | 1 | 1 | 1 | 1 | 1 | 1 | 1 | 1 | 1 |
| smi_1631 | -1 | -1 | -1 | -1 | -1 | -1 | -1 | -1 | -1 | 1 | -1 |
| smi_1632 | 1 | 1 | 1 | 1 | 1 | 1 | 1 | 1 | 1 | 1 | 1 |
| smi_1633 | 1 | 1 | 1 | 1 | -1 | 1 | 1 | 1 | 1 | 1 | 1 |
| smi_1634 | 1 | 1 | 1 | 1 | 1 | 1 | 1 | 1 | 1 | 1 | 1 |
| smi_1635 | 1 | 1 | -1 | 1 | 1 | -1 | -1 | 1 | 1 | -1 | 1 |
| smi_1636 | 1 | 1 | 1 | 1 | 1 | 0 | 1 | 1 | 1 | 1 | 1 |
| smi_1637 | 1 | 1 | 1 | 1 | 1 | 1 | 1 | 1 | 1 | 1 | 1 |
| smi_1638 | 1 | 1 | 1 | 1 | 1 | 1 | 1 | -1 | 1 | 1 | 1 |
| smi_1639 | 1 | 1 | 1 | 1 | 1 | 1 | 1 | 1 | 1 | 1 | 1 |
| smi_1640 | 1 | 1 | 1 | 1 | 1 | 1 | 1 | 1 | 1 | 1 | 1 |
| smi_1641 | 1 | 1 | -1 | 0 | 1 | 1 | 1 | 0 | -1 | 1 | -1 |
| smi_1642 | 1 | 1 | 1 | 1 | 1 | 1 | 1 | 1 | 1 | 1 | 1 |
| smi_1643 | 1 | 1 | 1 | 1 | 1 | 1 | 1 | 1 | 1 | 1 | 1 |
| smi_1644 | 1 | 1 | 1 | 1 | 1 | 1 | 1 | 0 | 1 | 1 | 0 |
| smi_1645 | 1 | 1 | 0 | 1 | 1 | 0 | -1 | 1 | 1 | 1 | -1 |
| smi_1646 | 1 | 1 | 1 | 1 | 1 | 0 | -1 | 0 | 1 | 1 | -1 |
| smi_1647 | 1 | 1 | 1 | 1 | 1 | -1 | -1 | 1 | 1 | 1 | -1 |
| smi_1648 | 1 | 1 | 1 | 1 | 1 | -1 | -1 | 1 | 1 | 1 | -1 |
| smi_1649 | 0 | 1 | -1 | 0 | 0 | -1 | -1 | -1 | 0 | 1 | 0 |
| smi_1650 | 1 | 1 | -1 | -1 | -1 | -1 | -1 | -1 | -1 | 1 | -1 |
| smi_1651 | 1 | 1 | -1 | -1 | 1 | -1 | -1 | -1 | 1 | 1 | -1 |
| smi_1652 | 0 | -1 | 1 | 0 | -1 | -1 | -1 | 1 | 1 | 0 | -1 |
| smi_1653 | 1 | 1 | -1 | -1 | -1 | -1 | -1 | -1 | 1 | 1 | -1 |
| smi_1654 | 1 | 1 | -1 | 1 | -1 | -1 | -1 | -1 | 1 | -1 | -1 |
| smi_1655 | 1 | -1 | -1 | -1 | -1 | -1 | -1 | -1 | -1 | -1 | -1 |
| smi_1656 | 1 | -1 | -1 | -1 | -1 | -1 | -1 | -1 | -1 | -1 | -1 |
| smi_1657 | 1 | -1 | -1 | -1 | -1 | -1 | -1 | -1 | 1 | -1 | -1 |
| smi_1658 | 1 | -1 | -1 | 1 | -1 | -1 | -1 | -1 | 1 | 0 | -1 |
| smi_1659 | 1 | 1 | 0 | 1 | 0 | 0 | 0 | 0 | 1 | 0 | -1 |
| smi_1660 | 1 | 1 | -1 | 1 | -1 | -1 | -1 | -1 | 1 | 1 | -1 |
| smi_1661 | 1 | 1 | -1 | 1 | -1 | -1 | -1 | -1 | 1 | -1 | -1 |
| smi_1662 | 1 | 1 | 0 | 1 | -1 | -1 | -1 | -1 | 1 | 1 | -1 |
| smi_1663 | 1 | 1 | 1 | 1 | 1 | 1 | 1 | 1 | 1 | 1 | 1 |
| smi_1664 | 1 | 1 | -1 | -1 | -1 | -1 | -1 | -1 | -1 | -1 | -1 |
| smi_1665 | 1 | 1 | 1 | 1 | 1 | 1 | 1 | 1 | 1 | 1 | 1 |
| smi_1666 | 1 | 1 | 1 | 1 | 1 | 1 | 1 | 1 | 1 | 1 | 1 |
| smi_1667 | 1 | 1 | 1 | 1 | 1 | 1 | 1 | 1 | 1 | 1 | 1 |
| smi_1668 | 1 | 1 | 1 | -1 | 1 | -1 | 1 | -1 | -1 | 1 | -1 |
| smi_1669 | 1 | 1 | 1 | 1 | 1 | 1 | 1 | 1 | 1 | 1 | 1 |
| smi_1670 | 1 | 1 | 1 | 1 | 1 | 1 | 1 | 1 | 1 | 1 | -1 |
| smi_1671 | 1 | 1 | 1 | 1 | 1 | 1 | 1 | 1 | 1 | 1 | 1 |
| smi_1672 | -1 | -1 | -1 | -1 | -1 | -1 | -1 | -1 | -1 | -1 | -1 |
| smi_1673 | -1 | -1 | -1 | -1 | -1 | -1 | -1 | -1 | -1 | -1 | -1 |
| smi_1674 | 1 | 1 | 1 | 1 | 1 | 1 | 1 | 1 | 1 | 1 | 1 |
| smi_1675 | 1 | 1 | 1 | 1 | 1 | 1 | 1 | 1 | 1 | 1 | 1 |
| smi_1676 | 1 | 1 | 1 | 1 | 1 | 1 | 1 | 1 | 1 | 1 | 1 |
| smi_1677 | 1 | 1 | 1 | 1 | 1 | 1 | 1 | 1 | 1 | 1 | 1 |
| smi_1678 | 1 | 1 | 1 | 1 | 1 | 1 | 1 | 1 | 1 | 1 | 1 |
| smi_1679 | 1 | 1 | 1 | 1 | 1 | 1 | 1 | 1 | 1 | 1 | 1 |
| smi_1680 | 1 | 1 | 1 | 1 | 1 | 1 | 1 | 1 | 1 | 1 | 1 |
| smi_1681 | 1 | 1 | 1 | 1 | 1 | 1 | 1 | 1 | 1 | 1 | 1 |
| smi_1682 | 1 | 1 | 1 | 1 | 1 | 1 | 1 | 1 | 1 | 1 | 1 |
| smi_1683 | 1 | 1 | 1 | 1 | 1 | 1 | 1 | 1 | 1 | 1 | 1 |
| smi_1684 | 1 | 1 | 1 | 1 | 1 | 1 | 1 | 1 | 1 | 1 | 1 |
| smi_1685 | 1 | 1 | 1 | 1 | 1 | 1 | 1 | 1 | 1 | 1 | 1 |
| smi_1686 | 1 | 1 | -1 | 0 | 1 | 1 | 1 | 1 | 1 | 1 | 1 |
| smi_1687 | 1 | 1 | 1 | 1 | 1 | 1 | 1 | 1 | 1 | 1 | 1 |
| smi_1688 | 1 | 1 | 1 | 1 | 1 | 1 | 1 | 1 | 1 | 1 | 1 |
| smi_1689 | 1 | 1 | 1 | 1 | 1 | 1 | 1 | 1 | 1 | 1 | 1 |
| smi_1690 | -1 | -1 | 1 | 1 | 1 | 1 | 1 | 0 | 1 | 1 | 1 |
| smi_1691 | -1 | 0 | -1 | 1 | -1 | -1 | -1 | -1 | -1 | -1 | -1 |
| smi_1692 | -1 | 1 | -1 | -1 | -1 | -1 | -1 | 1 | -1 | -1 | 1 |
| smi_1693 | -1 | 1 | 1 | -1 | -1 | -1 | -1 | 1 | -1 | -1 | 1 |
| smi_1694 | -1 | -1 | -1 | 0 | -1 | -1 | -1 | -1 | -1 | -1 | -1 |
| smi_1695 | -1 | -1 | -1 | -1 | -1 | -1 | -1 | -1 | -1 | -1 | -1 |
| smi_1696 | 0 | -1 | -1 | -1 | -1 | -1 | -1 | -1 | -1 | -1 | -1 |
| smi_1697 | -1 | -1 | -1 | -1 | -1 | -1 | -1 | -1 | -1 | -1 | -1 |
| smi_1698 | -1 | -1 | -1 | -1 | -1 | -1 | -1 | -1 | -1 | -1 | -1 |
| smi_1699 | -1 | -1 | -1 | -1 | -1 | -1 | -1 | -1 | -1 | -1 | -1 |
| smi_1700 | -1 | -1 | -1 | -1 | -1 | -1 | -1 | -1 | -1 | -1 | -1 |
| smi_1701 | -1 | -1 | -1 | -1 | -1 | -1 | -1 | -1 | -1 | -1 | -1 |
| smi_1702 | -1 | -1 | -1 | -1 | -1 | -1 | -1 | -1 | -1 | -1 | -1 |
| smi_1703 | -1 | -1 | -1 | -1 | -1 | -1 | -1 | -1 | -1 | -1 | -1 |
| smi_1704 | -1 | -1 | -1 | -1 | -1 | -1 | -1 | -1 | -1 | -1 | -1 |
| smi_1705 | -1 | -1 | -1 | -1 | -1 | -1 | -1 | -1 | -1 | -1 | -1 |
| smi_1706 | -1 | -1 | -1 | -1 | -1 | -1 | -1 | -1 | -1 | -1 | -1 |
| smi_1707 | -1 | -1 | -1 | -1 | -1 | -1 | -1 | -1 | -1 | -1 | -1 |
| smi_1708 | -1 | -1 | -1 | -1 | -1 | -1 | -1 | -1 | -1 | 0 | -1 |
| smi_1709 | -1 | -1 | -1 | -1 | -1 | -1 | -1 | -1 | -1 | -1 | -1 |
| smi_1710 | -1 | -1 | -1 | -1 | -1 | -1 | -1 | -1 | -1 | -1 | -1 |
| smi_1711 | -1 | -1 | -1 | -1 | -1 | -1 | -1 | -1 | -1 | -1 | -1 |
| smi_1712 | -1 | -1 | -1 | -1 | -1 | -1 | -1 | -1 | -1 | -1 | -1 |
| smi_1713 | 1 | 1 | -1 | -1 | -1 | -1 | -1 | -1 | -1 | -1 | -1 |
| smi_1714 | 1 | -1 | -1 | -1 | -1 | -1 | -1 | -1 | -1 | -1 | 0 |
| smi_1715 | 1 | -1 | -1 | -1 | -1 | -1 | -1 | -1 | -1 | -1 | -1 |
| smi_1717 | -1 | -1 | -1 | -1 | -1 | -1 | -1 | -1 | -1 | -1 | -1 |
| smi_1718 | 0 | -1 | -1 | 1 | -1 | -1 | -1 | -1 | -1 | -1 | -1 |
| smi_1719 | -1 | -1 | -1 | 1 | -1 | -1 | -1 | -1 | -1 | -1 | -1 |
| smi_1720 | 1 | 1 | 1 | 1 | 1 | 1 | 1 | 1 | 1 | 1 | 1 |
| smi_1721 | 1 | 1 | 1 | 1 | 1 | 1 | 1 | 1 | 1 | 1 | 1 |
| smi_1722 | 0 | 0 | 1 | 1 | 1 | 1 | 1 | 1 | 1 | 1 | 1 |
| smi_1723 | -1 | -1 | 1 | -1 | -1 | 1 | -1 | -1 | -1 | -1 | -1 |
| smi_1724 | 1 | 1 | 1 | -1 | -1 | 1 | -1 | -1 | -1 | 1 | -1 |
| smi_1725 | 1 | 1 | 1 | -1 | -1 | 1 | -1 | -1 | -1 | 1 | -1 |
| smi_1726 | 1 | 1 | 1 | -1 | -1 | 1 | -1 | -1 | -1 | -1 | -1 |
| smi_1727 | 1 | 1 | 1 | -1 | -1 | 1 | -1 | -1 | -1 | -1 | -1 |
| smi_1728 | 1 | 1 | 1 | -1 | -1 | 1 | -1 | -1 | -1 | -1 | -1 |
| smi_1729 | 1 | 1 | 1 | -1 | -1 | 1 | -1 | -1 | -1 | -1 | -1 |
| smi_1730 | 1 | 1 | 1 | -1 | -1 | 1 | -1 | -1 | -1 | -1 | -1 |
| smi_1731 | 1 | 1 | 1 | -1 | -1 | 1 | -1 | -1 | -1 | -1 | -1 |
| smi_1732 | 1 | 1 | 1 | -1 | -1 | 1 | -1 | -1 | -1 | -1 | -1 |
| smi_1733 | 1 | 1 | 1 | 1 | 1 | 1 | 1 | 1 | 1 | 1 | 1 |
| smi_1734 | 1 | 1 | 1 | 1 | 1 | 1 | 1 | 1 | 1 | 1 | 1 |
| smi_1735 | 1 | 1 | 1 | 1 | 1 | 1 | 1 | 1 | 1 | 1 | 1 |
| smi_1736 | 1 | 1 | 1 | 1 | 1 | 1 | 1 | 1 | 1 | 1 | 1 |
| smi_1737 | 1 | 1 | 1 | 1 | 1 | 1 | 1 | 1 | 1 | 1 | 1 |
| smi_1738 | 1 | 1 | 1 | 1 | 1 | 1 | 1 | 1 | 1 | 1 | 1 |
| smi_1739 | 1 | 1 | 1 | 1 | 1 | 1 | 1 | 1 | 1 | 1 | 1 |
| smi_1740 | -1 | -1 | -1 | 1 | -1 | 1 | 1 | -1 | 1 | -1 | 1 |
| smi_1741 | 1 | 1 | 0 | 1 | 1 | 1 | 1 | 1 | 1 | 1 | 1 |
| smi_1742 | 1 | 1 | 1 | 1 | 1 | 1 | 1 | 1 | 1 | 1 | 1 |
| smi_1743 | 1 | 1 | 1 | 1 | 1 | 1 | 1 | 1 | 1 | 1 | 1 |
| smi_1744 | 1 | 1 | 1 | 1 | 1 | 0 | 1 | 1 | 1 | 1 | 1 |
| smi_1745 | 1 | 1 | 1 | 1 | 1 | 1 | 1 | 1 | 1 | 1 | 1 |
| smi_1746 | 1 | 1 | 1 | 1 | 1 | 1 | 1 | 1 | 1 | 1 | 1 |
| smi_1747 | 1 | 1 | 1 | 1 | 1 | 1 | 1 | 1 | 1 | 1 | 1 |
| smi_1748 | 1 | 1 | 1 | 0 | 0 | 1 | -1 | -1 | 1 | 1 | -1 |
| smi_1749 | 1 | 1 | 1 | 1 | 1 | 1 | 1 | 1 | 1 | 1 | -1 |
| smi_1750 | 1 | 1 | 1 | 1 | 1 | 1 | 1 | 1 | 1 | 1 | 1 |
| smi_1751 | 1 | 1 | 1 | 1 | -1 | 1 | 1 | 0 | 1 | 1 | 1 |
| smi_1752 | 1 | 1 | 1 | 1 | 1 | 1 | 1 | 1 | 1 | 1 | 1 |
| smi_1754 | -1 | -1 | -1 | -1 | 1 | -1 | -1 | 1 | 0 | -1 | -1 |
| smi_1755 | 1 | 1 | 1 | 1 | -1 | 1 | 1 | 1 | 1 | 1 | -1 |
| smi_1756 | 0 | -1 | 1 | 1 | 1 | -1 | -1 | 1 | -1 | -1 | 1 |
| smi_1757 | 1 | 1 | 1 | -1 | 1 | 0 | -1 | 1 | -1 | -1 | -1 |
| smi_1758 | 1 | 1 | 1 | -1 | -1 | 0 | 1 | 1 | 1 | 1 | -1 |
| smi_1759 | 1 | 1 | -1 | -1 | 1 | -1 | 1 | 1 | 1 | 1 | 0 |
| smi_1760 | 1 | 1 | -1 | 1 | 1 | 1 | 1 | 1 | 1 | 1 | -1 |
| smi_1761 | 1 | 1 | 1 | 1 | 1 | 1 | 1 | 1 | 1 | 1 | 1 |
| smi_1762 | 1 | 1 | 1 | 0 | 1 | 1 | 1 | 1 | 1 | 1 | 1 |
| smi_1763 | 1 | 1 | 1 | 1 | 1 | 1 | 1 | 1 | 1 | 1 | 1 |
| smi_1764 | -1 | -1 | -1 | 0 | -1 | -1 | -1 | 1 | -1 | -1 | 1 |
| smi_1765 | 1 | 1 | 1 | 1 | 1 | 1 | 1 | 1 | 1 | 1 | 1 |
| smi_1766 | 1 | 1 | 1 | 1 | 1 | 1 | 1 | 1 | 1 | 1 | 1 |
| smi_1767 | 1 | -1 | -1 | -1 | 1 | -1 | 1 | 1 | 1 | 1 | -1 |
| smi_1768 | 1 | 1 | 1 | 1 | -1 | 1 | 1 | -1 | 1 | 1 | -1 |
| smi_1769 | 1 | 1 | 1 | 1 | 1 | 1 | 1 | 1 | 1 | 1 | 1 |
| smi_1770 | 1 | 1 | 1 | 1 | 1 | 1 | 1 | 1 | 1 | 1 | 1 |
| smi_1771 | -1 | -1 | -1 | -1 | -1 | -1 | -1 | -1 | -1 | -1 | -1 |
| smi_1772 | -1 | -1 | -1 | -1 | -1 | -1 | -1 | -1 | -1 | -1 | -1 |
| smi_1773 | 1 | 1 | 1 | 1 | 1 | 1 | 0 | 1 | 1 | 1 | -1 |
| smi_1774 | 1 | 1 | 1 | 1 | 1 | 1 | 1 | 1 | 1 | 1 | 1 |
| smi_1775 | 1 | 1 | 1 | 1 | -1 | -1 | 1 | -1 | 1 | 1 | -1 |
| smi_1776 | 1 | 1 | 1 | 1 | -1 | 1 | 1 | -1 | 1 | 1 | -1 |
| smi_1777 | 1 | 1 | 1 | 1 | -1 | 1 | 1 | -1 | 1 | 1 | -1 |
| smi_1778 | 1 | 1 | 1 | 1 | -1 | 1 | 1 | -1 | 1 | 1 | -1 |
| smi_1779 | 1 | 1 | 1 | 1 | 1 | 1 | -1 | -1 | -1 | -1 | -1 |
| smi_1780 | 1 | 1 | -1 | 1 | 1 | -1 | -1 | 1 | -1 | -1 | -1 |
| smi_1781 | -1 | 1 | -1 | -1 | -1 | -1 | -1 | 1 | -1 | -1 | -1 |
| smi_1782 | -1 | 1 | -1 | -1 | -1 | -1 | -1 | -1 | -1 | -1 | -1 |
| smi_1783 | 1 | 1 | 1 | -1 | -1 | 0 | -1 | -1 | -1 | 0 | -1 |
| smi_1784 | 1 | 1 | 1 | -1 | -1 | 1 | -1 | -1 | -1 | -1 | -1 |
| smi_1785 | -1 | -1 | -1 | -1 | -1 | -1 | -1 | -1 | -1 | -1 | -1 |
| smi_1786 | 0 | -1 | -1 | -1 | -1 | -1 | -1 | -1 | -1 | -1 | -1 |
| smi_1787 | 0 | -1 | -1 | -1 | -1 | -1 | -1 | -1 | -1 | -1 | -1 |
| smi_1788 | 0 | -1 | -1 | -1 | -1 | -1 | -1 | -1 | -1 | -1 | -1 |
| smi_1789 | -1 | -1 | -1 | -1 | -1 | -1 | -1 | -1 | -1 | -1 | -1 |
| smi_1790 | 0 | -1 | -1 | -1 | -1 | -1 | -1 | -1 | -1 | -1 | -1 |
| smi_1791 | -1 | -1 | -1 | -1 | -1 | -1 | -1 | -1 | -1 | -1 | -1 |
| smi_1792 | -1 | -1 | -1 | -1 | -1 | -1 | -1 | -1 | -1 | -1 | -1 |
| smi_1793 | -1 | -1 | -1 | -1 | -1 | -1 | -1 | -1 | -1 | -1 | -1 |
| smi_1794 | -1 | -1 | -1 | -1 | -1 | -1 | -1 | -1 | -1 | -1 | -1 |
| smi_1795 | -1 | -1 | -1 | -1 | -1 | -1 | -1 | -1 | -1 | -1 | -1 |
| smi_1796 | 1 | 1 | 1 | 1 | 1 | 0 | 1 | 0 | 1 | 0 | 1 |
| smi_1797 | 1 | 1 | 1 | 1 | 1 | 1 | 1 | 1 | 1 | 1 | 1 |
| smi_1799 | 1 | 0 | 1 | 1 | 1 | 1 | 1 | 1 | 1 | 1 | 1 |
| smi_1800 | 1 | 1 | 1 | 1 | 1 | 1 | 1 | 1 | 1 | 1 | 1 |
| smi_1801 | 1 | 1 | 1 | 1 | 1 | 1 | 1 | 1 | 1 | 1 | 1 |
| smi_1802 | 1 | 1 | -1 | 1 | 1 | 1 | 1 | 1 | 1 | -1 | 1 |
| smi_1803 | 1 | 1 | 1 | 1 | 1 | 1 | 1 | 1 | 1 | 1 | 1 |
| smi_1804 | 1 | 1 | 1 | 1 | 1 | 1 | -1 | 1 | -1 | 1 | 1 |
| smi_1805 | 1 | 1 | 1 | 1 | 1 | 1 | -1 | 1 | 1 | 1 | 1 |
| smi_1806 | 1 | 1 | 1 | 1 | 1 | 1 | 1 | 1 | 1 | 1 | 1 |
| smi_1807 | 1 | 1 | 1 | 1 | 1 | 1 | 1 | 1 | 1 | 1 | 1 |
| smi_1808 | 1 | 1 | 1 | 1 | 1 | 1 | 1 | 1 | 1 | 1 | 1 |
| smi_1809 | 1 | 1 | 1 | 1 | 1 | 1 | 1 | 1 | 1 | 1 | 1 |
| smi_1810 | 1 | 1 | 1 | 1 | 1 | 1 | -1 | 1 | 1 | -1 | 1 |
| smi_1811 | 1 | 1 | 1 | 1 | 1 | 1 | -1 | 1 | 1 | -1 | 1 |
| smi_1812 | 1 | 1 | 1 | 1 | 1 | 1 | -1 | 1 | 1 | -1 | 1 |
| smi_1813 | 1 | 1 | 1 | 1 | 1 | 1 | 1 | 1 | 1 | 1 | 1 |
| smi_1814 | 1 | 1 | 1 | 1 | 0 | 1 | 1 | 1 | 1 | 1 | 1 |
| smi_1815 | 1 | 1 | 1 | 1 | 1 | 1 | 1 | 1 | 1 | 1 | 1 |
| smi_1816 | 1 | 1 | 1 | 1 | 1 | 1 | 1 | 1 | 1 | 1 | 1 |
| smi_1817 | 1 | 1 | 1 | 1 | 1 | 1 | 1 | 1 | 1 | 1 | 1 |
| smi_1818 | 1 | 1 | 1 | 1 | 1 | 1 | 1 | 1 | 1 | 1 | 1 |
| smi_1819 | 1 | 1 | 1 | 1 | 1 | 1 | 1 | 1 | 1 | 1 | 1 |
| smi_1820 | 1 | 1 | 1 | 1 | 1 | 1 | 1 | 1 | 1 | 1 | 1 |
| smi_1821 | 1 | 1 | 1 | 1 | 1 | 1 | 1 | 1 | 1 | 1 | 1 |
| smi_1822 | 1 | 1 | 1 | 1 | 1 | 1 | 1 | 1 | 1 | 1 | 1 |
| smi_1823 | 1 | 1 | 1 | 1 | 1 | 1 | 1 | 1 | 1 | 1 | 1 |
| smi_1824 | 1 | 1 | 1 | 1 | 1 | 1 | 1 | 1 | 1 | 1 | 1 |
| smi_1825 | 1 | 1 | 1 | 1 | 1 | 1 | 1 | 1 | 1 | 1 | 1 |
| smi_1826 | 1 | 1 | 1 | 1 | 1 | 1 | 1 | 1 | 1 | 0 | 1 |
| smi_1827 | 1 | 1 | 1 | 1 | 1 | 1 | 1 | 1 | 1 | 1 | 1 |
| smi_1828 | 1 | 1 | 1 | 1 | 1 | 1 | 1 | 1 | 1 | 1 | 1 |
| smi_1830 | -1 | -1 | -1 | -1 | -1 | -1 | -1 | -1 | -1 | -1 | -1 |
| smi_1831 | 1 | 1 | 1 | 1 | 1 | 1 | 1 | -1 | 1 | -1 | -1 |
| smi_1832 | 1 | 1 | 1 | 1 | 1 | 1 | 1 | 1 | 1 | 1 | 1 |
| smi_1833 | 1 | 1 | 1 | 1 | 1 | 1 | 1 | 1 | 1 | 1 | -1 |
| smi_1834 | 1 | 1 | 1 | 1 | 1 | 1 | 1 | 1 | 1 | 1 | -1 |
| smi_1835 | 1 | 1 | 1 | 1 | 1 | 1 | 1 | 1 | 1 | 1 | 1 |
| smi_1836 | 1 | 1 | 1 | 1 | 1 | 1 | 1 | 1 | 1 | 0 | 0 |
| smi_1837 | 1 | 1 | 1 | 1 | 1 | 1 | 1 | 1 | 1 | 1 | 1 |
| smi_1838 | 1 | 1 | 1 | 1 | 1 | 1 | 1 | 1 | 1 | 1 | 1 |
| smi_1839 | 1 | 1 | 1 | 1 | 1 | 1 | 1 | 1 | 1 | 1 | 1 |
| smi_1840 | 1 | 0 | 1 | 1 | 1 | 1 | 1 | 1 | 1 | 1 | 1 |
| smi_1841 | 1 | 1 | 1 | 1 | -1 | 1 | 1 | 0 | 1 | 1 | -1 |
| smi_1842 | 1 | 1 | 1 | 1 | 1 | 1 | 1 | -1 | 1 | 1 | -1 |
| smi_1843 | 1 | 1 | 1 | 1 | 1 | 1 | 1 | 1 | 1 | 1 | 1 |
| smi_1844 | 1 | 1 | 1 | -1 | -1 | 1 | 1 | -1 | 1 | 1 | -1 |
| smi_1845 | 1 | 1 | 1 | 1 | 1 | 1 | 1 | 1 | 1 | 1 | 1 |
| smi_1846 | 1 | 1 | 1 | 1 | 1 | 1 | 1 | 1 | 1 | 1 | 1 |
| smi_1847 | 1 | 1 | 1 | 1 | 1 | 1 | 1 | 1 | 1 | 1 | 1 |
| smi_1848 | 1 | 1 | 1 | 1 | 1 | 1 | 1 | -1 | 1 | 1 | 1 |
| smi_1849 | 1 | 1 | 1 | 1 | 1 | 1 | 1 | -1 | -1 | 1 | 1 |
| smi_1850 | 1 | 1 | 1 | 1 | 1 | 1 | 1 | 1 | 1 | 1 | 1 |
| smi_1851 | 1 | 1 | 1 | 1 | 1 | 1 | 1 | -1 | 1 | 1 | 1 |
| smi_1852 | 1 | 1 | 1 | 1 | 1 | 1 | 1 | 1 | 1 | 1 | 1 |
| smi_1853 | 1 | 1 | 1 | 1 | 1 | 1 | 1 | 1 | 1 | 1 | 1 |
| smi_1854 | 1 | 1 | 1 | 1 | 1 | 1 | 1 | 1 | 1 | 1 | 1 |
| smi_1855 | 1 | 1 | 0 | 1 | 1 | 1 | 0 | 1 | 1 | 1 | 1 |
| smi_1857 | -1 | -1 | 0 | -1 | -1 | -1 | 0 | 1 | -1 | -1 | -1 |
| smi_1859 | 1 | 1 | 1 | 1 | 1 | 1 | 1 | 1 | 1 | 1 | 1 |
| smi_1860 | 1 | 1 | 1 | 1 | 1 | 1 | 1 | 1 | 1 | 1 | 1 |
| smi_1861 | 1 | 1 | 1 | 1 | 1 | 1 | 1 | 1 | 1 | 1 | 1 |
| smi_1862 | 1 | 1 | 1 | 1 | 1 | 1 | 1 | 1 | 1 | 1 | 1 |
| smi_1863 | -1 | -1 | 0 | -1 | -1 | -1 | 0 | 1 | -1 | -1 | -1 |
| smi_1866 | 1 | 1 | 1 | 1 | 1 | 1 | 1 | 1 | 1 | 1 | 1 |
| smi_1867 | 1 | 1 | 1 | 1 | 0 | 1 | 1 | 1 | 1 | 1 | 1 |
| smi_1868 | -1 | -1 | 1 | -1 | 1 | -1 | 1 | -1 | -1 | -1 | -1 |
| smi_1869 | -1 | -1 | 1 | -1 | 1 | -1 | 1 | -1 | -1 | -1 | -1 |
| smi_1870 | 0 | -1 | 1 | -1 | 0 | -1 | 1 | -1 | -1 | -1 | -1 |
| smi_1871 | -1 | -1 | 1 | -1 | 1 | -1 | 1 | -1 | -1 | -1 | -1 |
| smi_1872 | 1 | 1 | 1 | 1 | 1 | 1 | 1 | 1 | 1 | 1 | 1 |
| smi_1873 | 1 | 1 | 1 | 1 | 1 | 1 | 1 | 1 | 1 | 1 | 1 |
| smi_1875 | -1 | -1 | -1 | -1 | -1 | -1 | -1 | -1 | -1 | -1 | -1 |
| smi_1876 | 1 | 1 | 1 | 1 | 1 | 1 | 1 | 1 | 1 | 1 | 1 |
| smi_1877 | 1 | 1 | 1 | 1 | 1 | 1 | 1 | 1 | 1 | 1 | 1 |
| smi_1878 | 1 | 1 | 1 | 1 | 1 | 1 | 1 | 1 | 1 | 1 | 1 |
| smi_1879 | 1 | 1 | 1 | 1 | 1 | 1 | 1 | 1 | 1 | 1 | 1 |
| smi_1880 | 1 | 1 | 1 | 1 | 1 | 1 | 1 | 1 | 1 | 1 | 1 |
| smi_1881 | 1 | 1 | 1 | 1 | 1 | 1 | 1 | 1 | 1 | 1 | 1 |
| smi_1882 | 1 | 1 | 1 | 1 | 1 | 1 | 1 | 1 | 1 | 1 | 1 |
| smi_1883 | 1 | 1 | 1 | 1 | 1 | 1 | 1 | 1 | 1 | 1 | 1 |
| smi_1884 | 1 | 1 | 1 | 1 | 1 | 1 | 1 | 1 | 1 | 1 | 1 |
| smi_1885 | 1 | 1 | 1 | 1 | 1 | 1 | 1 | 1 | 1 | 1 | 1 |
| smi_1886 | 1 | 1 | 1 | 1 | 1 | 1 | 1 | 1 | 1 | 1 | 1 |
| smi_1887 | 1 | 1 | 1 | 1 | 1 | 1 | 1 | 1 | 1 | 1 | 1 |
| smi_1888 | 1 | 1 | 1 | 1 | 1 | 1 | 1 | 1 | 1 | 1 | 1 |
| smi_1889 | 1 | 1 | 1 | 1 | 1 | 1 | 1 | 1 | 1 | 1 | 1 |
| smi_1890 | 1 | 1 | 1 | 1 | 1 | 1 | 1 | 1 | 1 | 1 | 1 |
| smi_1891 | 1 | 1 | 1 | 1 | 1 | 1 | 1 | 1 | 1 | 1 | 1 |
| smi_1892 | 1 | 1 | 1 | 1 | 1 | 1 | 1 | 1 | 1 | 1 | 0 |
| smi_1893 | 1 | 1 | 1 | 1 | 1 | 1 | 1 | 1 | 1 | 1 | 1 |
| smi_1894 | 1 | 1 | 1 | 1 | 1 | 1 | 1 | 1 | 1 | 1 | 1 |
| smi_1895 | 1 | 1 | 1 | 1 | 1 | 1 | 1 | 1 | 1 | 1 | 1 |
| smi_1896 | 1 | 1 | 1 | 1 | 1 | 1 | 1 | 1 | 1 | 1 | 1 |
| smi_1897 | 1 | 1 | 1 | 1 | 1 | 1 | 1 | 1 | 1 | 1 | 1 |
| smi_1898 | 1 | 1 | 1 | 1 | 1 | 1 | 1 | 1 | 1 | 1 | 1 |
| smi_1899 | 1 | 1 | 1 | 1 | 1 | 1 | 1 | 1 | 1 | 1 | 1 |
| smi_1900 | 1 | 1 | 1 | 1 | 1 | 1 | 1 | 1 | 1 | 1 | 1 |
| smi_1901 | 1 | 1 | 1 | 1 | 1 | 1 | 1 | 1 | 1 | 1 | 1 |
| smi_1902 | 1 | 1 | 1 | 1 | 1 | 1 | 1 | 1 | 1 | 1 | 1 |
| smi_1903 | 0 | 1 | 1 | 1 | 1 | 1 | 1 | 1 | 1 | 1 | 1 |
| smi_1904 | 1 | 1 | 1 | 1 | 1 | 1 | 1 | 1 | 1 | 1 | 1 |
| smi_1905 | 1 | 1 | 1 | 1 | 1 | 1 | 1 | 1 | 1 | 1 | 1 |
| smi_1906 | 1 | 1 | 1 | 1 | 1 | 1 | 1 | 1 | 1 | 1 | 1 |
| smi_1908 | 1 | 1 | 1 | -1 | -1 | -1 | -1 | 1 | -1 | -1 | 1 |
| smi_1909 | 1 | 1 | 1 | 1 | 1 | 1 | 1 | 1 | 1 | 1 | 1 |
| smi_1910 | 1 | 1 | 1 | 1 | 1 | 1 | 1 | 1 | 1 | 1 | -1 |
| smi_1911 | 1 | 1 | 1 | 1 | 1 | 1 | 1 | 1 | 1 | 1 | 1 |
| smi_1912 | 1 | 0 | 1 | 1 | 1 | 1 | 1 | 1 | 1 | 1 | 1 |
| smi_1913 | -1 | -1 | -1 | 1 | 1 | -1 | -1 | 1 | -1 | -1 | 1 |
| smi_1914 | 1 | 1 | 1 | 1 | 1 | 1 | 1 | 1 | 1 | 1 | 1 |
| smi_1915 | 1 | 1 | 1 | 1 | -1 | 1 | 1 | -1 | 1 | 1 | 1 |
| smi_1916 | 1 | 1 | 1 | 1 | 1 | 0 | 1 | 1 | 1 | 1 | 1 |
| smi_1917 | 1 | 1 | 1 | 1 | 1 | 1 | 1 | 1 | 1 | 1 | 1 |
| smi_1918 | 1 | 1 | 1 | 1 | 1 | 1 | 1 | 1 | 1 | 1 | 1 |
| smi_1919 | 1 | 1 | 1 | 1 | 1 | 1 | 1 | 1 | 1 | 1 | 1 |
| smi_1920 | 1 | 1 | 1 | 1 | 1 | 1 | 1 | 1 | 1 | 1 | 1 |
| smi_1921 | 1 | 1 | 1 | 1 | 1 | 1 | 1 | 1 | -1 | -1 | 1 |
| smi_1922 | 1 | 1 | 1 | 1 | 1 | 1 | 1 | 1 | 1 | 1 | 1 |
| smi_1923 | 1 | 1 | 1 | 1 | -1 | 1 | 1 | 1 | 1 | 1 | -1 |
| smi_1924 | 1 | 1 | 1 | 1 | 1 | 1 | 1 | 1 | 1 | 1 | 1 |
| smi_1925 | 1 | 1 | 1 | 1 | 1 | 1 | 1 | 1 | 1 | 1 | 1 |
| smi_1926 | 1 | 1 | 1 | 1 | 1 | 1 | 1 | 1 | 1 | 1 | 1 |
| smi_1927 | 1 | 1 | 1 | 1 | 1 | 1 | 1 | 1 | 1 | 1 | 1 |
| smi_1928 | 1 | 1 | 1 | 1 | 1 | 1 | 1 | 1 | 1 | 1 | 1 |
| smi_1929 | 1 | 1 | 1 | 1 | 1 | 1 | 1 | 1 | 1 | 1 | 1 |
| smi_1930 | 1 | 1 | 1 | 1 | 1 | 1 | 1 | 1 | 1 | 1 | 1 |
| smi_1931 | 1 | 1 | 1 | 1 | 0 | 1 | 1 | 1 | 1 | 1 | 1 |
| smi_1932 | 1 | 1 | 1 | 1 | 1 | 1 | 1 | 1 | 1 | 1 | 1 |
| smi_1933 | 1 | 1 | 1 | 1 | 1 | 1 | 1 | 1 | 0 | 1 | 1 |
| smi_1934 | 1 | 1 | 1 | 1 | 1 | 0 | 1 | 1 | 1 | 1 | 1 |
| smi_1935 | 1 | 1 | 1 | 1 | 1 | 1 | 1 | 1 | 1 | 1 | 1 |
| smi_1936 | 1 | 1 | -1 | 1 | 1 | 1 | -1 | 1 | -1 | -1 | 0 |
| smi_1937 | 1 | 1 | 1 | 1 | 1 | 1 | 1 | 1 | 1 | 1 | 1 |
| smi_1938 | -1 | -1 | 1 | 1 | -1 | 1 | 1 | -1 | 1 | 1 | 1 |
| smi_1939 | -1 | -1 | 1 | 1 | -1 | 1 | 1 | -1 | 1 | 1 | 1 |
| smi_1940 | 1 | 1 | 1 | 1 | 1 | 1 | 1 | 1 | 1 | 1 | 1 |
| smi_1941 | 1 | 1 | 1 | 1 | 1 | 1 | 1 | 1 | 1 | 1 | -1 |
| smi_1942 | 1 | 1 | 1 | 1 | 1 | 1 | 1 | 1 | 1 | 1 | 1 |
| smi_1943 | 1 | 0 | 1 | -1 | 1 | 1 | 1 | 1 | 1 | 1 | 1 |
| smi_1944 | 1 | 0 | 1 | 1 | 1 | 1 | 1 | 1 | 1 | 1 | 1 |
| smi_1945 | 1 | 1 | 1 | 1 | 1 | 0 | 1 | 1 | 1 | 1 | 0 |
| smi_1946 | 1 | 1 | 1 | 1 | 1 | 1 | 1 | 1 | 1 | 1 | 1 |
| smi_1947 | 1 | 1 | 1 | 1 | 1 | 1 | 1 | 1 | 1 | 1 | 1 |
| smi_1948 | 1 | 1 | 1 | 1 | 1 | 1 | 1 | 1 | 1 | 1 | -1 |
| smi_1949 | 1 | 1 | -1 | -1 | -1 | -1 | -1 | -1 | -1 | 1 | 1 |
| smi_1950 | 1 | 1 | -1 | -1 | -1 | -1 | -1 | 1 | -1 | 1 | 1 |
| smi_1951 | 1 | 1 | 1 | 1 | 1 | 1 | 1 | 1 | 1 | 1 | 1 |
| smi_1952 | 1 | 1 | 1 | 1 | 1 | 1 | 1 | 1 | 1 | 1 | 0 |
| smi_1953 | 1 | 1 | 1 | 1 | 1 | 1 | 1 | 1 | 1 | 1 | 1 |
| smi_1954 | -1 | -1 | -1 | -1 | -1 | -1 | -1 | -1 | -1 | -1 | -1 |
| smi_1955 | -1 | -1 | -1 | -1 | -1 | -1 | -1 | 1 | -1 | -1 | 1 |
| smi_1956 | 1 | -1 | -1 | -1 | -1 | -1 | -1 | 1 | -1 | -1 | 1 |
| smi_1958 | 1 | 1 | 1 | 1 | 1 | 1 | -1 | 1 | -1 | 1 | 1 |
| smi_1959 | 1 | 1 | 1 | 1 | 1 | 1 | 1 | 1 | 1 | 1 | 1 |
| smi_1960 | 1 | 1 | 1 | 1 | 1 | 1 | 1 | 1 | 1 | 1 | 1 |
| smi_1961 | 1 | 1 | 1 | 1 | 1 | 1 | 1 | 1 | 1 | 1 | 1 |
| smi_1962 | 1 | 1 | 1 | 1 | 1 | 1 | 1 | 1 | 1 | 1 | 1 |
| smi_1963 | 1 | 1 | 1 | 1 | 1 | 1 | 1 | 1 | 1 | 1 | 1 |
| smi_1964 | -1 | -1 | -1 | -1 | -1 | -1 | -1 | -1 | -1 | -1 | -1 |
| smi_1965 | 1 | -1 | -1 | -1 | -1 | 1 | -1 | -1 | 1 | -1 | -1 |
| smi_1966 | 1 | -1 | -1 | -1 | -1 | 1 | -1 | -1 | 0 | 0 | -1 |
| smi_1967 | 1 | 1 | -1 | 1 | 1 | -1 | -1 | 1 | -1 | -1 | 1 |
| smi_1968 | 1 | 1 | -1 | 1 | 1 | -1 | -1 | 1 | -1 | -1 | 1 |
| smi_1969 | 1 | 1 | -1 | 1 | 1 | -1 | -1 | 1 | -1 | -1 | 1 |
| smi_1970 | -1 | 1 | 1 | 1 | 1 | -1 | 1 | 1 | -1 | -1 | 1 |
| smi_1971 | -1 | 1 | 1 | 1 | 1 | -1 | 1 | 1 | -1 | -1 | 1 |
| smi_1972 | 1 | 1 | 1 | 1 | 1 | 1 | 1 | 1 | -1 | -1 | -1 |
| smi_1973 | -1 | -1 | -1 | -1 | -1 | -1 | -1 | -1 | -1 | -1 | -1 |
| smi_1974 | -1 | -1 | -1 | -1 | 0 | 0 | -1 | -1 | -1 | -1 | -1 |
| smi_1975 | -1 | -1 | -1 | -1 | -1 | -1 | -1 | -1 | -1 | -1 | -1 |
| smi_1976 | -1 | -1 | -1 | -1 | -1 | -1 | -1 | -1 | -1 | -1 | -1 |
| smi_1977 | 1 | 1 | 1 | 1 | -1 | 1 | 1 | 1 | -1 | -1 | 1 |
| smi_1978 | 1 | 1 | 1 | 1 | 1 | 1 | 1 | 1 | 1 | 1 | -1 |
| smi_1979 | 1 | 1 | 1 | 1 | 1 | 1 | 1 | 1 | 1 | 1 | 1 |
| smi_1980 | 1 | 1 | 1 | 1 | 1 | 1 | 1 | 1 | 1 | 1 | 1 |
| smi_1981 | 1 | 1 | 1 | 1 | 0 | 1 | 1 | 1 | 1 | 1 | 1 |
| smi_1982 | 1 | 1 | 1 | 1 | 1 | 1 | 1 | 1 | 1 | 1 | 1 |
| smi_1983 | 1 | 1 | 1 | -1 | 1 | 1 | -1 | 1 | 1 | 1 | -1 |
| smi_1984 | 1 | 1 | 1 | 1 | 1 | 1 | 1 | 1 | 1 | 1 | 1 |
| smi_1985 | 1 | 1 | 1 | 1 | 1 | 1 | 1 | 1 | 1 | 1 | 1 |
| smi_1986 | 1 | 1 | 1 | 1 | 1 | 1 | 1 | 1 | -1 | 1 | 1 |
| smi_1987 | 1 | 1 | 1 | 1 | 1 | 1 | 1 | 1 | -1 | 1 | 1 |
| smi_1988 | 1 | 1 | 1 | 1 | 1 | 1 | 1 | 1 | -1 | 1 | 1 |
| smi_1989 | 1 | 1 | 1 | 1 | 1 | 1 | 1 | 1 | 1 | 1 | 1 |
| smi_1990 | 1 | 1 | 1 | 1 | 1 | 1 | 1 | 1 | 1 | 1 | 1 |
| smi_1991 | -1 | 1 | 1 | 1 | 0 | 1 | 1 | -1 | 1 | 1 | -1 |
| smi_1992 | -1 | 1 | -1 | 1 | -1 | 1 | 1 | -1 | 1 | 0 | 1 |
| smi_1993 | -1 | 1 | 1 | 1 | -1 | 1 | 1 | 1 | 1 | 1 | 1 |
| smi_1994 | -1 | 1 | 1 | 1 | -1 | 1 | 1 | 1 | 1 | 1 | 1 |
| smi_1995 | -1 | 1 | 1 | 1 | -1 | 1 | 1 | 1 | 1 | 1 | 1 |
| smi_1996 | -1 | 1 | 1 | 1 | -1 | 1 | 1 | -1 | 1 | 1 | -1 |
| smi_1997 | -1 | 1 | 1 | 1 | -1 | 1 | 1 | -1 | 1 | 1 | 1 |
| smi_1998 | -1 | 1 | 1 | 1 | -1 | 1 | 1 | -1 | 1 | 1 | 1 |
| smi_1999 | -1 | 0 | 0 | 1 | -1 | 1 | 1 | -1 | 1 | 1 | 1 |
| smi_2000 | 1 | 1 | -1 | -1 | 0 | 1 | 1 | -1 | -1 | -1 | -1 |
| smi_2001 | 1 | 1 | -1 | -1 | -1 | -1 | 1 | -1 | -1 | 1 | -1 |
| smi_2002 | 1 | -1 | -1 | -1 | -1 | -1 | 1 | -1 | -1 | -1 | -1 |
| smi_2003 | 1 | -1 | -1 | -1 | -1 | -1 | 1 | -1 | -1 | -1 | -1 |
| smi_2004 | 1 | -1 | -1 | -1 | -1 | -1 | -1 | -1 | -1 | -1 | -1 |
| smi_2005 | 1 | -1 | -1 | -1 | -1 | -1 | -1 | -1 | -1 | -1 | -1 |
| smi_2006 | 1 | -1 | -1 | -1 | -1 | -1 | 1 | -1 | -1 | -1 | -1 |
| smi_2007 | 1 | -1 | -1 | -1 | -1 | -1 | 1 | -1 | -1 | -1 | -1 |
| smi_2008 | 1 | -1 | -1 | -1 | -1 | -1 | 1 | -1 | -1 | -1 | -1 |
| smi_2009 | -1 | -1 | -1 | -1 | -1 | -1 | 1 | -1 | -1 | -1 | -1 |
| smi_2010 | 1 | 1 | -1 | 1 | 1 | 1 | 1 | 1 | -1 | 1 | -1 |
| smi_2011 | 1 | -1 | -1 | -1 | -1 | 0 | 1 | -1 | -1 | 1 | -1 |
| smi_2012 | -1 | -1 | -1 | -1 | -1 | -1 | -1 | -1 | -1 | -1 | -1 |
| smi_2013 | 1 | -1 | -1 | -1 | -1 | -1 | 1 | -1 | -1 | -1 | -1 |
| smi_2014 | -1 | -1 | -1 | -1 | -1 | -1 | -1 | -1 | -1 | -1 | -1 |
| smi_2015 | -1 | 1 | -1 | -1 | -1 | -1 | -1 | 1 | -1 | -1 | -1 |
| smi_2016 | -1 | -1 | -1 | -1 | -1 | -1 | -1 | -1 | -1 | -1 | -1 |
| smi_2017 | 1 | -1 | -1 | -1 | -1 | -1 | 1 | -1 | -1 | -1 | -1 |
| smi_2018 | -1 | 1 | 1 | 1 | -1 | 1 | 1 | -1 | 1 | 1 | 1 |
| smi_2019 | -1 | 1 | 1 | 1 | 1 | 1 | -1 | -1 | -1 | -1 | 1 |
| smi_2020 | -1 | 1 | 1 | 1 | 1 | 1 | -1 | -1 | -1 | -1 | -1 |
| smi_2021 | -1 | 1 | 1 | 1 | 1 | 1 | -1 | -1 | -1 | -1 | -1 |
| smi_2022 | 1 | 1 | 1 | 1 | 1 | 1 | 0 | 0 | 1 | 1 | 0 |
| smi_2023 | 1 | 1 | 1 | 1 | -1 | -1 | -1 | -1 | -1 | -1 | -1 |
| smi_2024 | 1 | 1 | 1 | 1 | 1 | 1 | 1 | 1 | 1 | 1 | 1 |
| smi_2025 | 1 | 1 | 1 | 1 | 1 | 1 | 1 | 1 | 1 | 1 | 1 |
| smi_2026 | 1 | 1 | 1 | 1 | 1 | 1 | 1 | 1 | 1 | 1 | 1 |
| smi_2027 | 1 | 1 | 1 | 1 | 1 | 1 | 1 | 1 | 1 | 1 | 1 |
| smi_2028 | 1 | 1 | 1 | 1 | 1 | 1 | 1 | -1 | -1 | -1 | 0 |
| smi_2029 | 1 | 1 | 1 | 1 | 1 | 1 | 1 | 1 | 1 | 1 | 1 |
| smi_2030 | 1 | 1 | 1 | 1 | 1 | 1 | 1 | 1 | 1 | 1 | 1 |
| smi_2031 | 1 | 1 | 1 | 1 | 1 | 1 | 1 | 1 | 1 | 1 | 1 |
| smi_2032 | 1 | 1 | 1 | 1 | 1 | 1 | 1 | 1 | -1 | -1 | 1 |
| smi_2033 | 1 | 1 | 1 | 1 | 1 | 1 | 1 | 1 | 1 | 1 | 1 |
| smi_2034 | 1 | 1 | 1 | 1 | 1 | 1 | 1 | 1 | 1 | 1 | 0 |
| smi_2035 | 1 | 1 | 1 | 1 | 1 | 1 | 1 | 1 | 1 | 1 | 1 |
| smi_2036 | 1 | 1 | 1 | 1 | 1 | 1 | 1 | 1 | 1 | 1 | 1 |
| smi_2037 | 1 | 1 | 1 | 1 | 1 | 1 | 1 | 1 | 1 | 1 | 1 |
| smi_2038 | 1 | 1 | 1 | 1 | 1 | 1 | 1 | 1 | 1 | 1 | 1 |
| smi_2039 | -1 | -1 | -1 | -1 | -1 | -1 | -1 | -1 | -1 | -1 | -1 |
| smi_2040 | -1 | -1 | -1 | -1 | -1 | -1 | -1 | -1 | -1 | -1 | 0 |
| smi_2041 | 1 | 1 | 1 | 1 | 1 | 0 | 0 | -1 | 1 | -1 | -1 |
| smi_2042 | 1 | 1 | 1 | -1 | 1 | 1 | 1 | -1 | 1 | 1 | -1 |
| smi_2043 | 1 | 1 | 1 | -1 | 1 | 1 | 1 | -1 | 1 | 1 | -1 |
| smi_2044 | 1 | 1 | 1 | -1 | 1 | 1 | 1 | -1 | 1 | 1 | -1 |
| smi_2045 | 1 | 1 | 1 | 1 | 1 | 1 | 1 | 1 | 1 | 1 | 1 |
| smi_2046 | 1 | 1 | 1 | 1 | 1 | 1 | 1 | 1 | 1 | 1 | 1 |
| smi_2047 | 1 | 1 | 1 | 1 | 1 | 1 | 1 | 1 | 1 | 1 | 1 |
| smi_2048 | 1 | 1 | 1 | 1 | 1 | 1 | 1 | 1 | 1 | 1 | 1 |
| smi_2049 | 1 | 1 | 1 | 1 | 1 | 1 | 1 | 1 | 1 | 1 | 1 |
| smi_2050 | 1 | 1 | 1 | 1 | 1 | 1 | 1 | 1 | 1 | 1 | 1 |
| smi_2051 | 1 | 1 | 1 | 1 | 1 | 1 | 1 | 1 | 1 | 1 | 1 |
| smi_2052 | 1 | 1 | 1 | 1 | 1 | 1 | 1 | 1 | 1 | 1 | 1 |
| smi_2053 | 1 | 1 | 1 | 1 | 1 | 1 | 1 | 1 | 1 | 1 | 1 |
| smi_2054 | 1 | 1 | 1 | 1 | 1 | 1 | 1 | 1 | 1 | 1 | 1 |
| smi_2055 | 1 | 1 | 1 | 1 | 1 | 1 | 1 | 1 | 1 | 1 | 1 |
| smi_2056 | 1 | 1 | 1 | 1 | 1 | 1 | 1 | 1 | 1 | 1 | 1 |
| smi_2057 | 1 | -1 | 1 | 1 | 1 | 1 | 1 | 1 | 1 | 1 | 1 |
| smi_2058 | 1 | 1 | 1 | 1 | 1 | 1 | 1 | 1 | 1 | 1 | 1 |
| smi_2059 | 1 | 1 | 1 | 1 | 1 | 1 | 1 | 1 | 1 | 1 | 1 |
| smi_2060 | 1 | 1 | 1 | 1 | 1 | 1 | 1 | 1 | 1 | 1 | 1 |
| smi_2061 | 1 | 1 | 1 | 1 | 1 | 1 | 1 | 1 | 1 | 1 | 1 |
| smi_2062 | 1 | 1 | 1 | 1 | 1 | 1 | 1 | 1 | 1 | 1 | 1 |
| smi_2063 | 1 | 1 | 1 | 1 | 1 | 1 | -1 | 1 | -1 | -1 | 1 |
| smi_2064 | 1 | 1 | 1 | 1 | 1 | 1 | 1 | 1 | 1 | 1 | 1 |
| smi_2065 | 1 | 1 | 1 | 1 | 1 | 1 | 1 | 1 | 1 | 1 | 1 |
| smi_2066 | 1 | 1 | 1 | 1 | 1 | 1 | 1 | 1 | 1 | 1 | 1 |
| smi_2067 | 1 | 1 | 1 | 1 | 1 | 1 | 1 | 1 | 1 | 1 | 1 |
| smi_2068 | 1 | 1 | 1 | 1 | 1 | 1 | 1 | 1 | 1 | 1 | 1 |
| smi_2069 | 1 | 1 | 1 | 1 | 1 | 1 | 1 | 1 | 1 | 1 | 1 |
| smi_2070 | 1 | 1 | 1 | 1 | 1 | 1 | 1 | 1 | 1 | 1 | 0 |
| smi_2071 | 1 | 1 | 1 | 1 | 1 | 1 | 1 | 1 | 1 | 1 | 1 |
| smi_2072 | 1 | 1 | 0 | 1 | 1 | 0 | 1 | 1 | 1 | 1 | 1 |
| smi_2073 | 1 | 1 | 1 | 1 | 1 | 1 | 1 | 1 | 1 | 1 | 1 |
| smi_2074 | 1 | 1 | 1 | 1 | 1 | 1 | 1 | 1 | 1 | 1 | 1 |
| smi_2075 | 1 | 1 | 1 | 1 | 1 | 1 | 1 | 1 | 1 | 1 | 1 |
| smi_2076 | 1 | 1 | 1 | 1 | 1 | 1 | 1 | 1 | 1 | 1 | 1 |
| smi_2077 | 1 | 1 | 1 | 1 | 1 | 1 | 1 | 1 | 1 | 1 | 1 |
| smi_2078 | 1 | 1 | 1 | 1 | 1 | 1 | 1 | 1 | 1 | 1 | 1 |
| smi_2079 | 1 | 1 | 1 | 1 | 1 | 1 | 1 | 0 | 0 | 1 | 1 |
| smi_2080 | 1 | 1 | 1 | 1 | 1 | 1 | 1 | 1 | 1 | 1 | 1 |
| smi_2083 | 1 | 1 | 1 | 1 | 1 | 1 | 1 | 1 | 1 | 1 | 1 |
| smi_2084 | 1 | 1 | 1 | 0 | 1 | -1 | -1 | 1 | 1 | 1 | 1 |
| smi_2085 | 1 | 1 | 1 | 1 | 1 | 1 | 1 | 1 | 1 | 1 | 1 |
| smi_2086 | 1 | 1 | 1 | 1 | 1 | 1 | 1 | 1 | 1 | 1 | 1 |
| smi_2087 | 1 | 1 | 1 | 1 | 1 | 1 | 1 | 1 | 1 | 1 | -1 |
| smi_2088 | 1 | 1 | 1 | 1 | 1 | 1 | 1 | 1 | 1 | 1 | 1 |
| smi_2089 | 1 | 1 | 1 | 1 | 1 | 1 | 1 | 1 | 1 | 1 | 1 |

There are 2023 features (70mers) included in the analysis, and evaluated as described in the method section. Hybridization signals are indicated by +1 (positive), -1 (negative), or ambigious (0).
