# Supplementary material for: The Genome of Streptococcus mitis B6 - What Is a Commensal?
Source: PLoS One. 2010 Feb 25;5(2):e9426. doi: 10.1371/journal.pone.0009426 (PMC2828477; doi:10.1371/journal.pone.0009426)
Supplement: Table S4 — A. Hybridization of S. mitis with oligonucleotides corresponding to S. pneumoniae virulence factors. B. Oligonucleotides. Oligonucleotides specific for S. pneumoniae R6 (spr) or TIGR4 (SP) were used in comparative hybridization using S. mitis DNA. +1: positive signals; negative signals: −1; 0 corresponds to ambiguous signals. (0.05 MB DOC) [file pone.0009426.s005.doc]

Table S4.

A. Hybridization of *S. mitis* with oligonucleotides corresponding to *S. pneumoniae* virulence factors.

| reference gene | gene product |  |  |  | *S. mitis* |  |  |  |  |  |  |  |  |
| --- | --- | --- | --- | --- | --- | --- | --- | --- | --- | --- | --- | --- | --- |
|  |  |  | B5 | Huo8 | SV5 | 658 | Huo1 | 10712 | SV10 | RSA4 | 697 | M3 | B6 |
| SP0117 | *pspA* | pneumococcal surface protein A | -1 | -1 | -1 | -1 | -1 | -1 | -1 | -1 | -1 | -1 | -1 |
| spr0121 | *pspA* | surface protein pspA precursor | -1 | -1 | -1 | -1 | -1 | -1 | 0 | -1 | -1 | -1 | -1 |
| spr1945 | *pcpA* | choline-binding protein | -1 | -1 | -1 | -1 | -1 | -1 | -1 | -1 | -1 | -1 | -1 |
| spr1995 | *pspC* | choline binding protein A | -1 | 0 | -1 | -1 | -1 | -1 | -1 | -1 | -1 | -1 | -1 |
| spr0286 | *hysA* | hyaluronidase/hyase | -1 | -1 | -1 | -1 | -1 | -1 | -1 | -1 | -1 | -1 | -1 |
| spr1739 | *ply* | pneumolysin | 0 | 0 | 0 | 0 | +1 | -1 | -1 | +1 | 0 | -1 | -1 |
| spr1754 | *lytA* | autolysin (N-acetylmuramoyl-L-alanine amidase) | -1 | +1 | -1 | -1 | -1 | -1 | -1 | +1 | -1 | -1 | -1 |
| spr1042 | *iga* | Immunoglobulin A1 protease | -1 | 0 | -1 | 0 | -1 | -1 | +1 | 0 | +1 | +1 | 0 |

Oligonucleotides specific for *S. pneumoniae* R6 (spr) or TIGR4 (SP) were used in comparative hybridization using *S. mitis* DNA. +1: positive signals; negative signals: -1; 0 corresponds to ambiguous signals.

B. Oligonucleotides

| SP0117 | *pspA* | TTTAACAAGTCTAGCCAGCGTCGCTATCTTAGGGGCTGGTTTTGTTACGTCTCAGCCTACTTTTGTAAGA |
| --- | --- | --- |
| spr0121 | *pspA* | TGCGTCTCAGCCTACTGTTGTAAGAGCAGAAGAATCTCCCGTAGCCAGTCAGTCTAAAGCTGAGAAAGAC |
| spr1945 | *pcpA* | TAGAGAAACTAACATTACCAAAATCGGTTAAAACATTAGGAAGTAATCTATTTAGACTCACTACTAGCTT |
| spr1995 | *pspC* | GGTAAACCAAAGGGGCGGGCAAAACGAGGAGTTCCTGGAGAGCTAGCAACACCTGATAAAAAAGAAAATG |
| spr0286 | *hysA* | TTGAGGAAAGTGGTAAGGACAAGCGATTGTGGAATTCTGCAACGACGTCAGGAACAAAGGACTGGCAGAC |
| spr1739 | *ply* | AAGTAGCGATAGCTTTCTCCAAGTGGAAGACCCCAGCAATTCAAGTGTTCGCGGAGCGGTAAACGATTTG |
| spr1754 | *lytA* | AAAACGCACGAGTATTGCACGAATAACCAACCAAACAACCACTCAGACCACGTTGACCCTTATCCATATC |
| spr1042 | *iga* | TCTTGCCTCTCCTGACCTTGCCAGAGAGATCCGTATTTGTCATCTCGACCATGTCTAGTCTAGGATTTGG |
